# Dual-function floating-gate memory driver for energy-efficient integrated display–illumination system

**DOI:** 10.1038/s41467-026-76263-3

**Published:** 2026-08-03

**Authors:** Haifeng Wu, Yizhe Wang, Qijun Zong, Jiali Yi, Huawei Liu, Xingxia Sun, Qin Shuai, Jian Hu, Xiao Lin, Yazhou Wang, Penghui Guo, Xudong Yang, Tanghao Xie, Xiaoqin Liao, Ben Wang, Xiaoming Zhao, Xiaoli Zhu, Bobo Tian, Xiao Wang, Dong Li, Anlian Pan

**Affiliations:** 1 Key Laboratory for Micro-Nano Physics and Technology of Hunan Province, State Key Laboratory of Advanced Design and Manufacturing Technology for Vehicle, Hunan Institute of Optoelectronic Integration, College of Materials Science and Engineering, Hunan University, Changsha, China; 2 School of Physics and Electronics, Hunan Normal University, Changsha, China; 3 Innovision Technology (Suzhou) Co., Ltd, Suzhou, China; 4 East China Normal University, Shanghai, China; 5 FuRong Laboratory, Changsha, China

**Keywords:** Electronic devices, Electronic devices, Lasers, LEDs and light sources

## Abstract

The increasing demand for multifunctional micro-displays capable of both high-brightness illumination and low-power image display highlights a critical bottleneck in existing display technologies, which struggle to efficiently switching between large-area uniform light output and fine-grained pixel-level control. Here, we introduce the Integrated Nonvolatile Display–Illumination System (INDIS)—a monolithically integrated Micro-LED architecture based on transparent indium tin oxide (ITO) floating-gate memory (FGM) devices, achieving dual-mode operation. Pixel-level addressing is realized with only a single FGM per pixel and an independent gate controller, enabling the programmed image pattern to be retained without continuous refresh. To support lighting functionality, INDIS adopts a matrix circuit with shared drain electrodes acting as a common anode port, allowing the entire Micro-LED array to be simultaneously activated for large-area illumination. By unifying memory and emission functions within a compact architecture, INDIS achieves display–illumination fusion, offering a scalable and energy-efficient solution for next-generation multifunctional micro-display applications.

## Abstract (web summary)

Micro-display capable of both illumination and image is a critical bottleneck in display technologies. Wu et al. report an integrated nonvolatile display-illumination system, offering a scalable and energy efficient solution for the switching between lighting functionality and controllable display.

## Introduction

The rapid evolution of next-generation wearable, augmented reality, and projection systems is driving an urgent need for highly integrated micro-displays that simultaneously deliver high-resolution imaging and high-brightness illumination within compact, energy-efficient platforms^1–5^. Micro-light-emitting diodes (Micro-LEDs), after nearly a decade of development, have emerged as the most promising emissive technology for this purpose^6,7^, offering significant advantages in high-pixel-density displays with resolutions ranging from several thousand to over ten thousand pixels per inch (PPI)^8–16^. Furthermore, with the advancements in material growth and chip fabrication, Micro-LEDs have gradually overcome the brightness degradation associated with device miniaturization, now achieving ultrahigh-brightness emission levels exceeding 10^7 ^Cd·m^−2^—well-suited for illumination scene^17–20^. Yet, current Micro-LED display architectures still fall short in realizing true integration of display and illumination functions^21–23^. These functions are typically implemented in physically and electrically separate modules, leading to increased system volume, power consumption, and control complexity^24–26^. Moreover, achieving programmable, pixel-level control within Micro-LED arrays remains reliant on complex peripheral circuits or high-refresh-rate driving schemes, which fundamentally limits the ability to retain static images efficiently and hinders reconfigurable functionality under limited energy budgets^27,28^.

To address these limitations, significant efforts have been devoted toward the in-situ integration of Micro-LED arrays with thin-film transistors (TFTs)—including those based on traditional amorphous silicon, indium gallium zinc oxide (IGZO), organic materials, and emerging two-dimensional (2D) semiconductor materials, aiming to achieve compact, high-resolution pixel-level control^25,29^. For example, Hun et al. fabricated a monochrome Micro-LED display integrated with Si TFTs by directly utilizing a silicon substrate after mesa formation, achieving a simplified fabrication process and successful device operation^30^. Kim et al. developed a pixel circuit based on a heterostructure of IGZO/Te films deposited via RF sputter on GaN wafers, achieving pulse width modulation (PWM) for high-performance Micro-LED display^31^. Guo et al. reported a wafer-scale, monolithically integrated micro-display driven by organic TFT arrays fabricated using low- temperature solution processes, demonstrating excellent uniformity and reliability^32^. In addition, molybdenum disulfide (MoS_2_), a typical 2D material obtained through either chemical vapor deposition (CVD) growth or mechanical exfoliation, has demonstrated significant potential for monolithic Micro-LED integration due to its superior electrical characteristics^33–35^. These integrated solutions offer improved signal routing, reduced interconnect complexity, and real-time modulation of light output at the pixel level. However, such systems remain fundamentally volatile—requiring constant power to retain display states—and lack the capability to store and maintain patterns without refresh. As a result, they are poorly suited for applications demanding static imaging, low-power operation, or real-time reconfiguration^36,37^. Moreover, their circuit architectures are typically optimized for display only purposes, without support for large-area, uniform light emission, making it difficult to realize truly multifunctional platforms that unify illumination and imaging capabilities. This gap highlights an urgent need for a dual-functional circuit architecture that can support both persistent image retention and dynamic pattern switching, while also enabling chip-level display–illumination integration within a compact footprint and low-power envelope^38,39^.

To address these challenges, we develop the Integrated Nonvolatile Display–Illumination System (INDIS), a monolithically integrated Micro-LED display architecture that combines nonvolatile pixel control and chip-level display-lighting fusion. INDIS is based on transparent floating-gate memory (FGM) devices fabricated on 4-inch ITO wafers with high yield and device uniformity. Each integrated 1M1D enables static pattern retention without refresh and ultra-low-power reconfiguration at the pixel level, achieving only 11.71% voltage drop across the memory, a static power consumption of 1.4 pW in the off state, and a switching energy of merely 11.41 pJ per gate voltage pulse. Through a shared drain common anode matrix design, the system also supports simultaneous activation of the entire Micro-LED array for uniform large-area illumination. By unifying display and lighting functionalities within a compact and energy-efficient platform, INDIS provides a scalable solution for next-generation micro-display applications, including wearable electronics, compact projectors, and augmented reality systems.

## Results

### Fabrication and structural characterization

**Fig. 1:**
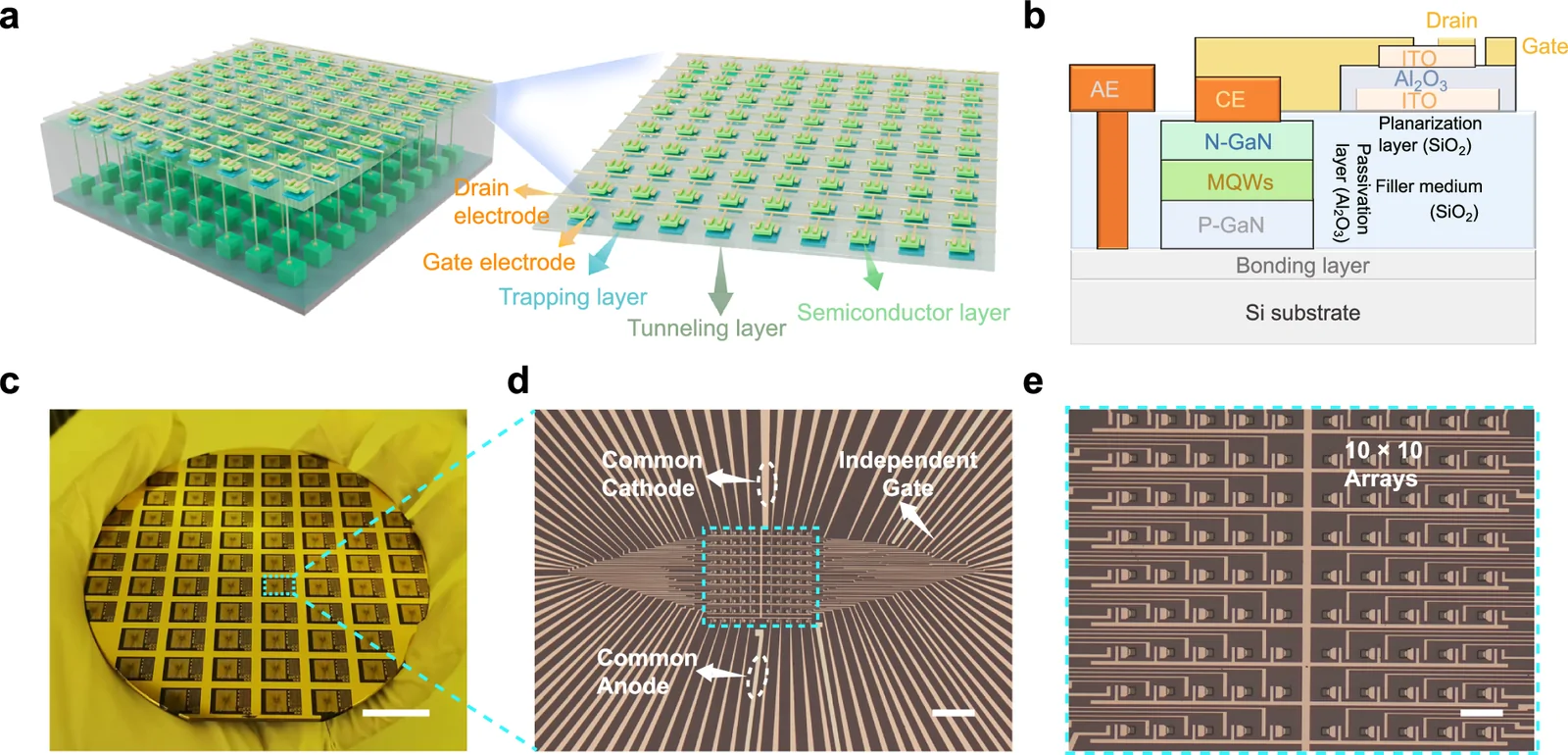
Schematic illustration of the integrated nonvolatile display-illumination system (INDIS). **a** Schematic illustration of the Micro-LED display integrated with FGMs devices, where the driving circuit amplification principle is shown on the right side. **b** Schematic illustration of the cross-section of the integrated chip. **c** Optical micrographs of the wafer-scale integration chips fabricated by standard lithography and etching process. Scale bar, 2 cm. Zoomed-in integration chips of the arrays. Scale bars, 200 μm and 100 μm for (**d**) and **e** respectively.

Figure 1a displays a schematic illustration of the display-illumination system, which comprises two functional layers. The bottom layer hosts Micro-LEDs fabricated through standard semiconductor processing based on high-quality GaN-based epitaxial structures grown on 4-inch Si (111) substrates, where transmission electron microscope (TEM) images indicate a sharp interface of InGaN/GaN in multiple quantum wells (Supplementary Fig. 1), confirming the excellent crystalline quality of the epitaxial layers^40,41^. Details of the material composition are provided in the Methods section. The top layer consists of a driving circuit based on floating-gate memory (FGM), with the source electrode of each FGM device directly connected to the anode of the underlying Micro-LED through a patterned metal interconnect. Details of the fabrication process are provided in the methods section, and Supplementary Fig. 2. Detailed device structure of the driving circuits is illustrated on the right side of Fig. 1a. Each FGM device is composed of multiple functional layers, including a charge trapping layer, a semiconductor layer and a tunneling dielectric layer. The drain and source electrodes are deposited on the semiconductor layer, with the drain nodes shared across the array to form a common anode. The gate electrode is located above the tunneling dielectric and aligned in the same plane as the semiconductor layer, ensuring strong capacitive coupling. Notably, all electrodes in the FGM stack are deposited in a single evaporation step, greatly simplifying circuit fabrication and system integration.

Figure 1b presents a cross-sectional schematic of a single pixel, illustrating the vertically stacked architecture between the GaN-based Micro-LED and the ITO-based FGM. The integrated device comprises ~6 nm transparent conductive ITO films as the charge trapping layers (Supplementary Fig. 2), and an ~18 nm Al_2_O_3_ as the tunneling dielectric layer, and ~6 nm transparent ITO films as the semiconductor, forming the layered structure for in-situ driver integration for Micro-LED displays^42^. Different drive modes can be realized by applying voltage pulses to either the common anode or gate electrode, effectively modulating carrier injection paths to achieve display and lighting functions. Detailed working mechanisms of different drive modes are provided in Supplementary Fig. 3. Figure 1c depicts an optical micrography of fabricated chips on a 4-inch wafer, demonstrating wafer-scale process compatibility, high uniformity, and excellent yield. A 10 × 10 pixel array is shown in Fig. 1d, with a magnified view of the central area provided in Fig. 1e, clearly illustrating the compact and precisely aligned integration of Micro-LEDs with the ITO-based FGMs.

### Electrical performance of floating-gate memories

**Fig. 2:**
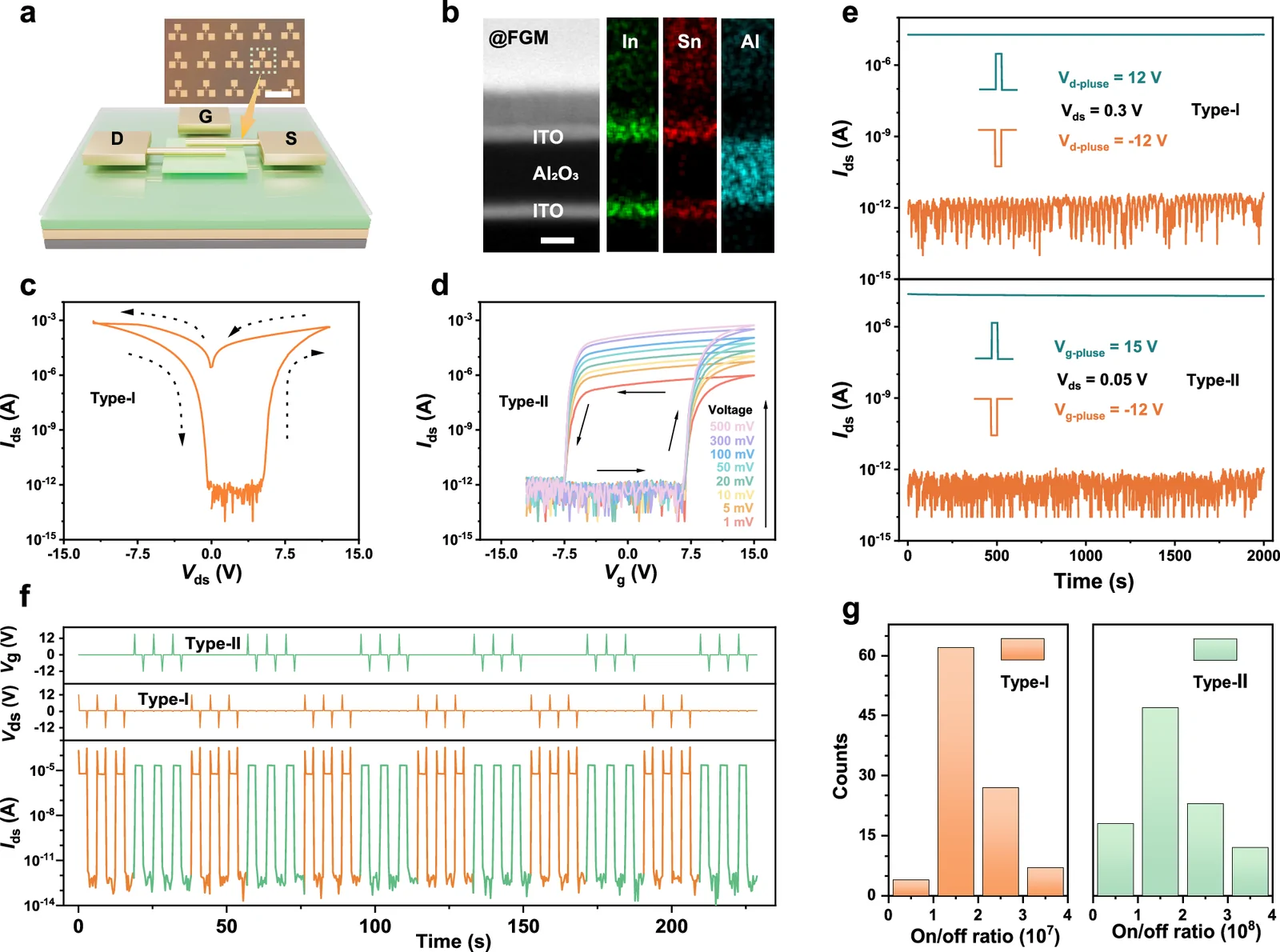
Electrical characterization of ITO-based floating-gate memory (FGM) devices. **a** Schematic diagram of the FGM device structure. The inset shows an optical image of a device array fabricated on GaN-based epitaxial wafers after mesa formation. Scale bar, 300 μm. **b** TEM images of the cross-section of functional layers in the FGM. Scale bars, 10 nm. Sweep transfer characteristics of the device controlled by drain pulse voltage from −12 V to +12 V in type-I mode (**c**), and gate pulse voltage from −12 V to +15 V in type-II mode (**d**). **e** Retention performance of the device measured at room temperature with different modes. Pulse width: 1 s. **f** The switching performance of the device between the type-I and type-II modulation modes. **g** Statistics of on/off ratio from 100 different devices.

The electrical performances of ITO-based FGMs were systematically evaluated. As illustrated in Fig. 2a, the device adopts a planar three-terminal architecture, in which the source, drain, and control gate are co-fabricated within the same device plane, and the channel width and length are 16 μm and 1 μm, respectively. Figure 2b shows a cross-sectional image of a single pixel ITO FGM acquired by TEM, revealing a highly planar morphology of each functional layer. This uniformity results from the chemical mechanical planarization (CMP) of the SiO_2_ isolation layer, which ensures high-quality ITO film deposition and improved device performance. Energy-dispersive spectroscopy (EDS) mapping further confirms the distinct boundaries between the layers within the integrated stack. This configuration supports dual-mode operation through distinct biasing schemes (Supplementary Fig. 3). In type-I mode, the program/erase function is realized by applying voltage to the drain electrode, enabling charge injection into the conductive ITO-based charge trapping layer (CTL) via Fowler–Nordheim tunneling through the Al_2_O_3_ dielectric. The charge tunneling and schematic band diagrams are shown in Supplementary Fig. 4. The I-V characteristics (Fig. 2c) reveal a clear memory window of 5.2 V under a sweep voltage from −12 to 12 V, with an on-current exceeding 10^‒5 ^A and an off-current as low as 5 × 10^‒13 ^A, yielding an on/off ratio exceeding 10^7^. Notably, the two-terminal configuration exhibits pronounced I-V hysteresis resembling that of memristive devices, providing inherent memory behavior without requiring a gate terminal^43,44^. Such characteristics are particularly beneficial for global control of large-area Micro-LED arrays, where uniform switching across all pixels is desired for illumination applications. By enabling collective programming and erasing of pixels through simplified biasing, this mode facilitates system-level light modulation with reduced circuit complexity and power overhead.

In type-II mode, the device functions as a three-terminal floating-gate transistor, where programming and erasing are performed via gate voltage pulses. The transfer characteristics (Fig. 2d) exhibit pronounced hysteresis, with a large memory window of 14.5 V with gate voltage sweeping from −12 to 15 V. In comparison, Supplementary Fig. 5 shows the transfer curves of an ITO-based TFT, which display hysteresis-free behavior with depletion-mode characteristics. This comparison suggests that the observed memory behavior can be reasonably attributed to charge trapping in the FG, rather than defect-related trapping mechanisms. The corresponding operating schematics under different pulse polarities are illustrated in Supplementary Fig. 6. The threshold voltage remains consistently around 6.7 V regardless of the drain bias, demonstrating enhancement-mode memory behavior that is essential for low-power operation. Moreover, the operation voltage and power consumption can be further optimized by employing a thinner Al_2_O_3_ tunneling layer. As shown in Supplementary Figs. 7 and 8, when the tunneling layer is reduced to 10 nm, the programing/erase voltages can be readily lowered to below 10 V. Additional electronic performances for the FGM devices with different channel lengths are provided in Supplementary Fig. 9. Unlike the globally addressable type-I mode, the type-II configuration enables independent, pixel-level modulation, allowing precise and reconfigurable control of static image content. This per-pixel programmability is particularly advantageous for high-resolution display applications, where images can be retained without constant refreshing, enabling ultra-low power consumption. The device also exhibits good stability, further enhancing the reliability of static displays (Supplementary Figs. 10 and 11).

Long-term retention performance is then evaluated under both operation modes (Fig. 2e and Supplementary Fig. 12). For type-I mode, programming and erasing are realized via drain voltage pulses of ±12 V, while the current is monitored under a small read bias of 0.3 V. In type-II mode, gate voltage pulses of –12 V and +15 V (pulse width~1 s) are employed for erasing and programming, with the readout performed at zero gate and drain bias of 0.1 V. In both cases, stable current levels were maintained with minimal degradation, indicating reliable charge retention. This dual-mode programmability supports both static image holding and dynamic light modulation, offering reconfigurable functionality essential for next-generation display-illumination platforms. The switching dynamics under hybrid modulation and standalone mode are shown in Fig. 2f and Supplementary Fig. 13, respectively, with both modes maintaining a high on/off ratio, especially exceeding 10^9^ In type-II mode at *V*_ds_ = 0.5 V. Notably, initialization (resetting the device’s conductivity state) is not strictly required for every mode transition, and there is negligible interference between Type-I and Type-II modes. Moreover, readout currents could be switched rapidly between on and off states by applying 50 μs voltage pulses to the gate terminals, corresponding to a refresh frequency in the order of 5 kHz, substantially higher than that of conventional advanced dynamic displays^45^, indicating that the device can also support both static image retention and high-speed dynamic operation (Supplementary Figs. 13 and 14). To assess the integration potential of the proposed architecture, a statistical analysis was conducted on 100 FGM devices (Fig. 2g), revealing average on/off ratios of 1.83 × 10^7^ and 1.75 × 10^8^ in type-I and type-II modes, respectively. And chip-to-chip statistical analyses on a 4-inch wafer are provided in Supplementary Figs. 15 and 16. The performance values were tightly clustered around the mean, indicating excellent device uniformity. A fabrication yield exceeding 98% further highlights the robustness and scalability of the FGM array for large-area integration. Supplementary Table S1 provides a systematic comparison of device structure and electronic performance of our ITO-based FGMs with previously reported FGMs, highlighting superior performances in terms of on-state current and on/off current ratio, thus laying an important foundation for high-performance, low-power display drivers.

### Integration and electrical performance of unit pixels

**Fig. 3:**
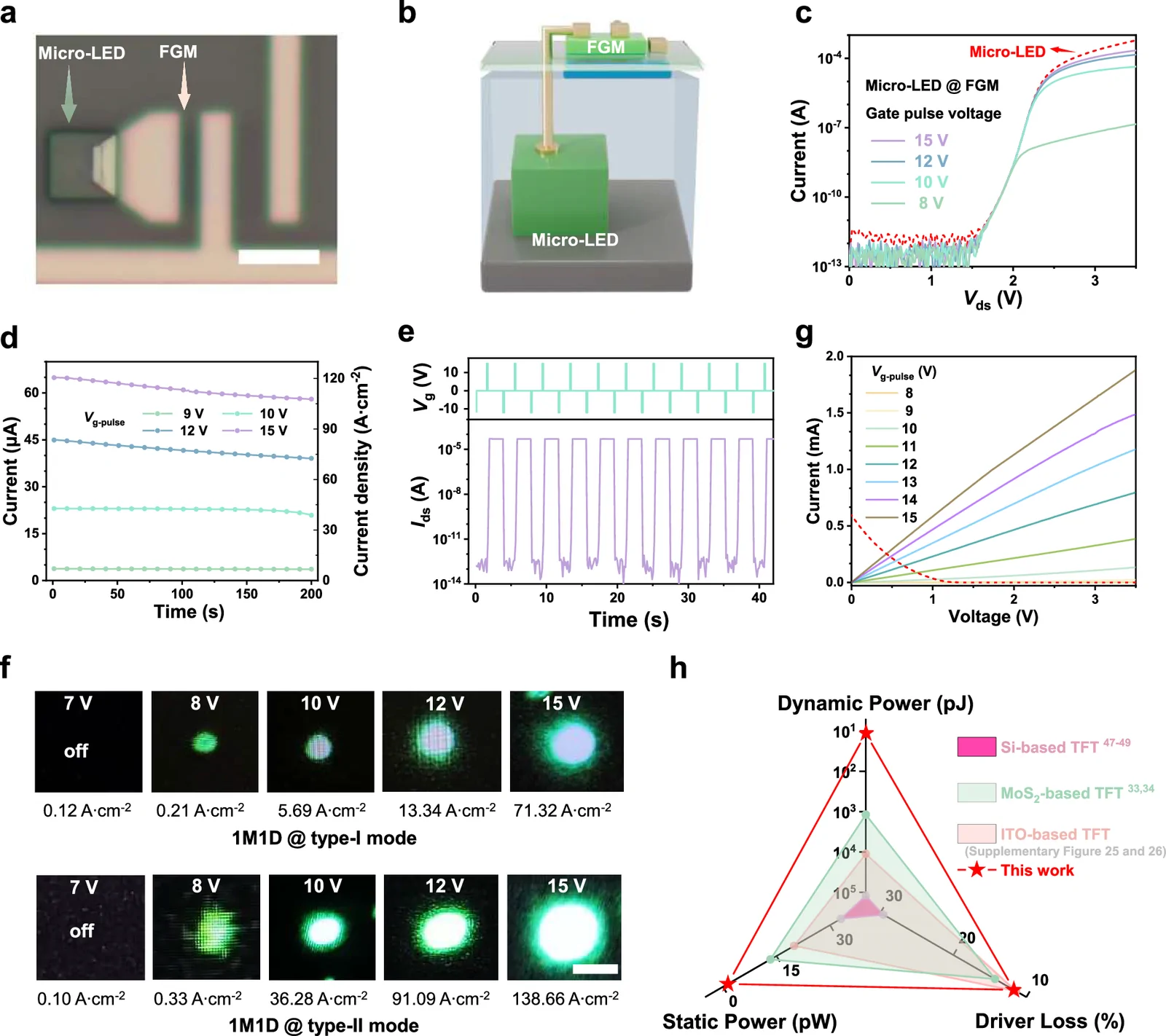
Individual Micro-LED integrated with ITO-based FGM. **a** Optical micrography of the 1M1D pixel. Scale bar, 10 μm. **b** Schematic diagram of the 1M1D structure. **c** The IV characteristics of the 1M1D and Micro-LED (red dashed line) in log scale. **d** The retention characteristics of the 1M1D under different gate voltages. **e** The on/off current of the integrated device under the state of programmed (15 V & 0.1 s) and erased (−12 V & 0.1 s), exhibiting the on/off ratio over 10^8^. **f** Micro-LED brightness of ITO-based memory integrated with type-I/II modes. Scale bar, 10 μm. **g** The IV curve of the device controlled by the pulse voltage forwarded on the gate, all pulse widths are one hundred milliseconds, where the solid lines and red dashed lines represent ITO-based FGMs and Micro-LED, respectively. **h** Statistics of power consumption levels for different Micro-LED pixel driver circuits.

To demonstrate practical integration of FGM for an integrated display-illumination application, a monolithic 1M1D architecture was realized by integrating the ITO-based FGM with GaN-based Micro-LEDs, as illustrated in Fig. 3a. A Micro-LED size of 7.5 μm was employed, with FGM-enabled modulation serving as the driver to configure pixel states without the need for a continuous power supply, as shown in Fig. 3b. Current characteristics of the 1M1D pixel were first measured over a wide range of drain and gate voltage sweeps. Similar to the stand-alone memory, a large memory window was observed, confirming that both bias and gate voltage pulses can effectively regulate the circuit current and thereby enable reliable switching of Micro-LED pixels (Supplementary Fig. 17). Figure 3c presents the current-voltage (I-V) characteristics of the integrated 1M1D unit under different gate pulse voltages, alongside the I–V curve of a stand-alone Micro-LED. The Micro-LED exhibits typical diode-like rectification, with a large on-state current of 594 μA at a drain-source voltage of 3.5 V and an on/off ratio exceeding 10^8^, indicating overall high diode quality with low on-state contact resistance. Furthermore, the external quantum efficiency (EQE) characteristics was displayed in the Supplementary Fig. 18, showcasing moderate reductions, which demonstrate effective thermal management enabled by the device structure (Supplementary Fig. 19). In the interconnected 1M1D configuration, the on-state current under 15 V gate pulses approaches that of the standalone Micro-LED (217 μA, corresponding to a current density of 385.7 A·cm^‒2^), demonstrating small voltage drop across the memory and implying minimal power consumption in the transistor during full-on operation (further power analysis is discussed in the following part). Moreover, by varying the amplitude of the gate pulses, the on-state current at a fixed drain-source voltage can be modulated, confirming effective brightness control by the memory element.

Under a fixed drain-source bias, the brightness of the Micro-LED can be effectively modulated by gate pulse voltages applied to the FGM, demonstrating nonvolatile behavior. Figure 3d shows the temporal evolution of the 1M1D circuit current read at a drain-source voltage of 2.8 V following different gate pulse voltages. It indicates that the on-state current varies from several microamperes to tens of microamperes, corresponding to Micro-LED current densities of 8.55-146.93 A·cm^‒2^, enabling effective grayscale control. The current also exhibits excellent long-term retention, with the Micro-LED remaining steadily illuminated for over 200 s with negligible decay in brightness (Supplementary video 1 and Supplementary Figs. 20 and  21), where all of the brightness retention remains above 90%, especially up to 96.87% under gate pulse voltages of 9 V (1.6 × 10^5 ^Cd·cm^‒2^), with a pulse width of 1 s. The light-emitting state could also be reliably switched between on and off by modulating the resistance of the memory transistor through gate pulse voltage (Fig. 3e and Supplementary Fig. 22), demonstrating effective light on/off control. Moreover, such switching can be performed at high speed, further demonstrating compatibility with dynamic display scenarios, similar to those achieved with 1T1C or 2T1C drivers^46^. As shown in Fig. 3f, the brightness of the pixel was effectively modulated by two operation modes. Under a constant read voltage of 2.8 V, the Micro-LED could be driven from the off to the on state by adjusting the gate pulse voltage. Below 7 V, the device remained off due to insufficient carrier tunneling into the charge-trap layer. As the gate pulse voltage increased, the brightness of the pixel cell also increased. Supplementary Fig. 23 illustrates the brightness levels under various test conditions, marking the maximum brightness up to 4.6 × 10^6 ^Cd·m^‒2^ at 138.66 A·cm^‒2^, confirming excellent modulation controllability. Micro-LEDs with lateral dimensions of 15 μm and 30 μm were also successfully illuminated (Supplementary Fig. 24). This further demonstrates the compatibility of the memory-driven driving scheme with various pixel sizes.

To evaluate the contribution of the memory transistor to the overall power consumption of the 1M1D circuit—including on-state voltage drop, off-state leakage, switching energy, and refresh-related power—different aspects of the circuit were systematically analyzed. The output characteristics of the ITO-based FGM were measured after programming with gate voltage pulses of varying amplitudes (pulse width: 100 ms) and plotted alongside the I–V curve of the integrated Micro-LED (Fig. 3g). The maximum current is measured to be 1.9 mA, showcasing good driving ability. The intersection points between the FGM and Micro-LED curves represent the operating states of the integrated 1M1D pixel at a fixed source-drain bias, allowing direct extraction of the voltage drop across each component. As can be seen, following programming with a +15V pulse, the intersection occurs at a point where only 0.41 V is dropped across the memory, indicating that 88.29% of the total 3.5 V bias is applied to the Micro-LED. This low-voltage drop across the memory highlights its excellent on-state conductivity, enabling efficient current delivery to the Micro-LED. In the off state (Micro-LED is switched off), the circuit current is as low as 5 × 10^‒13 ^A. This corresponds to a static power consumption per pixel of only 1.4 × 10^−12 ^W at a bias voltage of 2.8 V, which is orders of magnitude lower than commercial values and can substantially extend the battery life of portable display–illumination systems. Gate leakage current—another critical contributor to the overall system power consumption—is only 7.61 × 10^−12 ^A under a 15 V gate bias, yielding a switching energy of merely 11.41 pJ per gate voltage pulse. Since no continuous gate voltage is required to maintain the display state, the theoretical power consumption associated with refresh driving is effectively reduced to nearly zero.

Figure 3h presents a polar comparison of the power consumption among representative Micro-LED driver circuits^33,34,47–49^, where the detailed computational specifics are provided in the Supplementary Figs. 25 and 26. Benefiting from the high carrier transport capability of ITO, the on-state current of the FGM and ITO driver is significantly larger than that of conventional Si and MoS_2_ channels, which is favorable for high-brightness Micro-LED driving, leading to low voltage drop and small energy consumption on the ITO TFT and FGM driver circuit. As compared with the TFT driver, the FGM operates under pulse programming without the need for continuous refresh, leading to a reduction by several orders in dynamic power consumption. Moreover, in the off-state, the FGM ensures complete channel depletion, and the wide bandgap of ITO suppresses leakage current, together resulting in ultralow static power consumption. Compared with recently reported capacitor-less active driving schemes, these synergistic merits endow the integrated ITO-based FGM drivers with distinctive advantages in both energy efficiency and data retention, highlighting their strong potential for next-generation low-power Micro-LED display systems (Supplementary Table 2).

### Design of micro-LED illumination-display system

Unlike conventional TFT-driven Micro-LED systems that require a continuous gate power supply, the INDIS system proposed in this work adopts nonvolatile ITO-based FGMs as drivers, thereby eliminating the need for sustained gate voltage during operation (Supplementary Fig. 27). The fabricated Micro-LED array comprises 10 × 10 pixels with a pitch of 45 μm, corresponding to a pixel density of 564 PPI. Each pixel consists of a 7.5-μm Micro-LED vertically integrated with an ITO-FGM element. The PPI of the INDIS can be further increased by reducing the channel width of the FGM and constructing vertically stacked integrated devices based on a fully transparent FGM structure. To construct the test system, peripheral control circuits were implemented on a main printed circuit board (PCB), providing precise pulse generation and timing control. The Micro-LED arrays were wire-bonded to a socket and subsequently soldered onto a transfer PCB, which housed embedded routing traces for signal distribution.

**Fig. 4:**
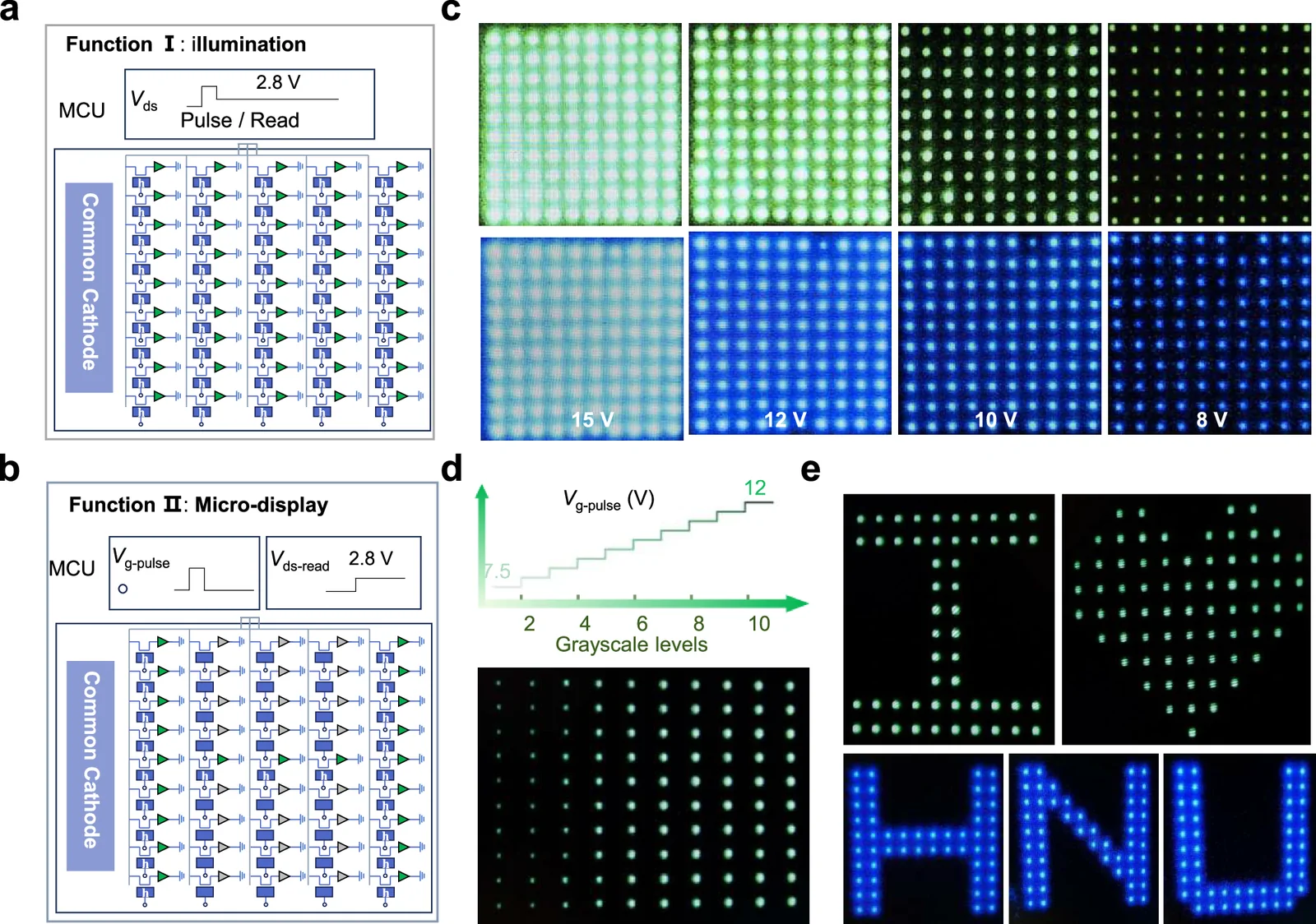
Two function modes for 10×10 arrays in INDIS with the Micro-LED size of 7.5 μm. Schematical for type-Ⅰ (**a**) and type-Ⅱ (**b**) modes in INDIS, respectively. **c** Optical images of Micro-LED arrays modulated in type-Ⅰ mode. **d** The brightness of each row modulated in type-Ⅱ mode (*V*_g_ from 7.5 V to 12 V), achieving different arrays. **e** Display of geometric letters and specific shape modulated in type-Ⅱ mode.

Figures 4a, b illustrate the drain modulation mode and gate modulation mode of the system, respectively. In the drain modulation mode (Fig. 4a), a programming pulse voltage is first applied to the anode, inducing hole trapping in the floating gate and thereby lowering the resistance of the semiconductor channel. Upon applying a read voltage of 2.8 V, all Micro-LEDs are uniformly turned on. As shown in Fig.4c, both green and blue Micro-LED arrays exhibit homogeneous emission without observable defective pixels, indicating high device yield and excellent luminance uniformity. Moreover, by adjusting the amplitude of the drain-source programming pulses, multiple brightness levels can be achieved, enabling adaptable operation from low- to high-intensity illumination.

In contrast, the gate modulation mode (Fig. 4b) enables selective programming of individual pixels. By applying voltage pulses to specific gate electrodes, holes are locally stored in the corresponding FGMs. Under a common read voltage applied at the anode, only the programmed pixels are activated, allowing programmable image display. As demonstrated in Fig. 4d, grayscale modulation can be realized at the row level by varying the gate pulse amplitude from 7.5 V to 12 V, enabling fine control over pixel brightness and improved display contrast. Finally, Fig. 4e shows the dynamic display of the pattern “I ♥ HNU (Hunan University)” on the 10 × 10 arrays. A larger 200 ×128 array capable of displaying the word “MICRO” is presented in Supplementary Fig. 28, further validating the system’s scalability. The results highlight the feasibility and versatility of our integrated nonvolatile display and illumination system, laying a solid foundation for future development of energy-efficient and multifunctional Micro-LED technologies.

## Discussion

In summary, we have demonstrated an integrated nonvolatile display-illumination system based on ITO-based floating-gate memories. The FGMs were successfully monolithically integrated with Micro-LED arrays using standard lithography and deposition processes on a 4-inch wafer, achieving high fabrication yield and excellent uniformity. Comprehensive characterization of two distinct modulation modes confirms the robust driving capability, programmable memory window, strong retention performance, and high on/off current ratio of the system. In Type-I mode, all Micro-LEDs can be uniformly illuminated with tunable brightness levels, enabling both high- and low-illumination scenarios. In Type-II mode, specific images can be displayed and retained for extended periods without gate power, demonstrating an ultra-low-power display functionality. We believe that the developed INDIS paves the way for lightweight, energy-efficient, and highly integrated Micro-LED-based electronic systems.

## Methods

### Fabrication of micro-LED arrays

The 4-inch blue and green GaN-based epitaxy wafers were first grown on 1000-μm thickness Si (111) by metal-organic chemical vapor deposition (MOCVD), which consists of a 1.5 μm thick AlN/AlGaN/GaN buffer layer, a 2 μm thick n-GaN layer, a 4-period pre-quantum wells, an 8-period InGaN/GaN emitting multiple quantum wells (MQWs), a p-type AlGaN/GaN superlattices and p-type GaN. Then, a 110-nm-thickness ITO layer was deposited by sputtering and annealed at 500 °C for 3 min to achieve good ohmic contact with p-GaN. Subsequently, stacked metal layers were evaporated and bonded to a prepared Si substrate. Next, the epitaxy of the original Si (111) substrate and buffer layers was removed by combining wet and dry methods. Through the standard lithography process, pixel definition was successfully achieved by an inductively coupled plasma (ICP) system, in which the exposed GaN layer was removed to form a pixel mesa with Cl_2_ and BCl_3_. Subsequently, the pixel mesas were covered with an atomic-level passivation layer (50-nm Al_2_O_3_) deposited by atomic layer deposition (ALD), which could effectively passivate defects caused by plasma etching and reduce the non-radiative recombination rate. SiO_2_ layer was deposited and planarized through a chemical mechanical polishing (CMP) process for obtaining an ultra-flat interface. Finally, the Ti / Au stack metal layers were evaporated to establish n and p electrodes by E-beam evaporation (EBE).

### Monolithic integration process

To integrate ITO-based FGMs, the wafer with Micro-LED arrays was transferred to the magnetron sputter chamber. First, a 6-nm ITO layer was deposited with argon at 100 °C, which was patterned to be a charge trap layer with lithography and a wet etching process. Then, 18-nm Al_2_O_3_ was deposited by ALD as a charge tunneling layer. Next, 6-nm ITO semiconductor material was deposited and patterned through the above process. The drain, source, gate and interconnected metal stack layers were evaporated (5 nm Cr/50 nm Au) at one time after opening the n/p electrodes windows of Micro-LED arrays (using Ar plasma ICP), which is aiming to establish the electrical interconnection between Micro-LEDs and FGMs through connecting the n electrodes with the source.

To integrate ITO-based TFTs, the back gate electrodes (5 nm Ti/20 nm Pd) were first deposited. Then, 18-nm Al_2_O_3_ was deposited by ALD as a gate dielectric layer. Subsequently, the semiconductor material was deposited and patterned through the same process as FGMs. Finally, the drain, source and interconnected metal stack were established.

### Material and device characterizations

The optical images of the Micro-LED arrays integrated with ITO-based FGMs were characterized through a microscope (Zeiss Axio Scope A1) and a camera of Nova 11. Cross-sections of TEM (HR-TEM) images were acquired with a transmission electron microscope (FEI Themis Z (3.2)). The electrical performance of the ITO-based FGMs was measured using an Agilent B1500 semiconductor parameter analyzer in a Lake Shore vacuum probe station (10^−5^ Pa). The optoelectronic characteristics of the Micro-LEDs integrated with FGMs were utilized with a self-built probe station with the spectroradiometer SR-5100HM coupled with a high-accuracy sphere system and a Keithley 2450 to supply voltage.

## Supplementary information


Supplementary Information
Description of Additional Supplementary Files
Supplementary video 1
Transparent Peer Review file


## Data Availability

The data of this report have been included in the published article and its Supplementary Information. Additional raw data are available from the corresponding authors upon request.
