# Supplementary material for: Dual-function floating-gate memory driver for energy-efficient integrated display–illumination system
Source: Nat Commun. 2026 Aug 3;17:9313. doi: 10.1038/s41467-026-76263-3 (PMC13530162; doi:10.1038/s41467-026-76263-3)
Supplement: Supplementary file 1 [file 41467_2026_76263_MOESM1_ESM.pdf]

Dual-function Floating-Gate Memory Driver for Energy-Efficient Integrated Display–Illumination System

Haifeng Wu[1†], Yizhe Wang[1†], Qijun Zong[1], Jiali Yi[1], Huwei Liu[2], Xingxia Sun[1], Qin Shuai[1], Jian Hu[1], Xiao Lin[1,3], Yazhou Wang[3], Penghui Guo[1], Xudong Yang[1], Tanghao Xie[1], Xiaoqin Liao[1], Ben Wang[1], Xiaoming Zhao[4], Xiaoli Zhu[1], Bobo Tian[4], Xiao Wang[1]*, Dong Li[1,5]*, and Anlian Pan[1,2]*

[1] Key Laboratory for Micro-Nano Physics and Technology of Hunan Province, State Key Laboratory of Advanced Design and Manufacturing Technology for Vehicle, Hunan Institute of Optoelectronic Integration, College of Materials Science and Engineering, Hunan University, Changsha, 410082, China

[2] School of Physics and Electronics, Hunan Normal University, Changsha, 410081, China

[3] Innovision Technology (Suzhou) Co., Ltd, Suzhou, 215000, China

[4] East China Normal University, Shanghai 200241, China.

[5] FuRong Laboratory, Changsha 410078, Hunan, China.

*Corresponding Author: liidong@hnu.edu.cn; xiao_wang@hnu.edu.cn; anlian.pan@hnu.edu.cn

Table of contents

Supplementary Note 1. Operation principle of different drive modes.

Supplementary Note 2. Performance of FGM devices with thinner tunneling layers.

Supplementary Note 3. Heat buildups under high-brightness conditions.

Supplementary Note 4. Energy consumption analysis of 1M1D.

Supplementary Figure 1. Cross-sectional TEM images of the Micro-LED.

Supplementary Figure 2. Fabrication process of INDIS.

Supplementary Figure 3. Working mechanisms of INDIS.

Supplementary Figure 4. Schematic illustration of memory operation in type-I mode.

Supplementary Figure 5. Transfer characteristics of an ITO-based TFT.

Supplementary Figure 6. Schematic illustration of memory operation in type-II mode.

Supplementary Figure 7. Performance of FGM devices with thinner tunneling layer.

Supplementary Figure 8. Current stability for FGM devices with different drain bias applied.

Supplementary Figure 9. Electrical characteristics of FGM devices with different channel length.

Supplementary Figure 10. Electrical stability and endurance of the FGM device.

Supplementary Figure 11. Threshold voltage stability of the FGM devices.

Supplementary Figure 12. Retention performance of FGM devices.

Supplementary Figure 13. Switching performance of FGM devices.

Supplementary Figure 14. Demonstration of high-frequency operation in type-II mode.

Supplementary Figure 15. Uniformity of FGM devices across a 4-inch wafer.

Supplementary Figure 16. Current distribution of FGM devices across a 4-inch wafer.

Supplementary Figure 17. Memory window of 1M1D pixel with different modulation modes.

Supplementary Figure 18. External quantum efficiency characteristics of the Micro-LED.

Supplementary Figure 19. Brightness retention characteristics of Micro-LED pixel.

Supplementary Figure 20. Retention characteristics of the 1M1D under different gate voltage.

Supplementary Figure 21. Retention characteristics of the 1M1D pixel.

Supplementary Figure 22. Stability and switching performance of 1M1D pixel.

Supplementary Figure 23. Brightness modulation of 1M1D pixel.

Supplementary Figure 24. Brightness modulation of the 1M1D pixel through voltage pulse.

Supplementary Figure 25. Transfer characteristics of 1T1D and 1M1D pixels.

Supplementary Figure 26. Output performance of 1T1D and 1M1D pixels.

Supplementary Figure 27. Optical microscope of the INDIS.

Supplementary Figure 28. Micro-LED display with large arrays of 200 × 128 pixels.

Supplementary Table S1. Recent advances in various floating-gate memory devices.

Supplementary Table S2. Power consumption and stability in Micro-LED devices with various driver technology.

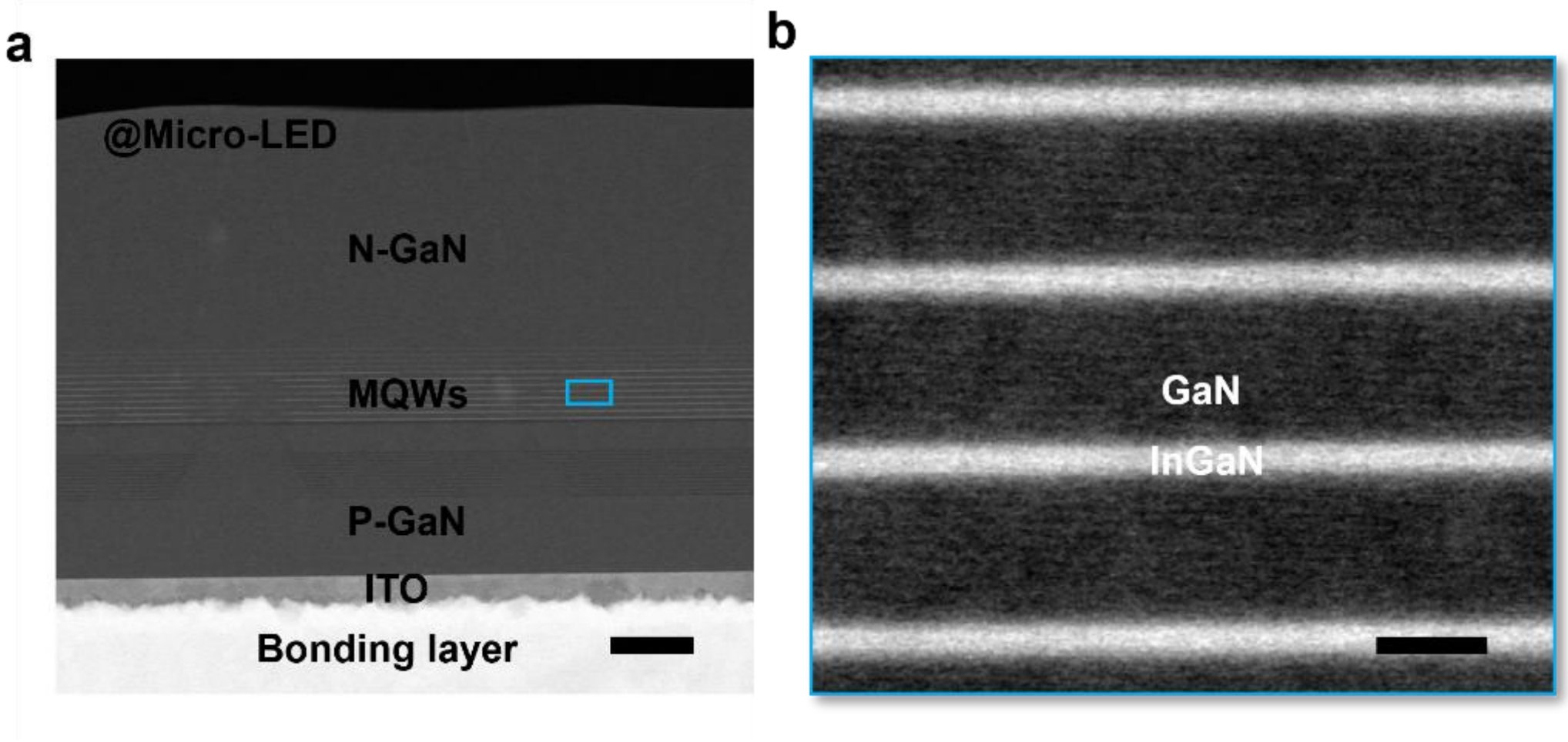

Supplementary Figure 1. Transmission electron microscope (TEM) images of a, The cross-section of Micro-LED epitaxy layers. Scale bars, 500 nm. **b**, Zoomed-in image of the interface of InGaN/GaN in multiple quantum wells. Scale bars, 10 nm. The 110-nm-thick ITO layer, as observed in Supplementary Figure 1a, was deposited on the epi-wafer via sputtering and subsequently annealed at 500 °C for 3 minutes. This process facilitates a high-quality Ohmic contact with the p-GaN layer.

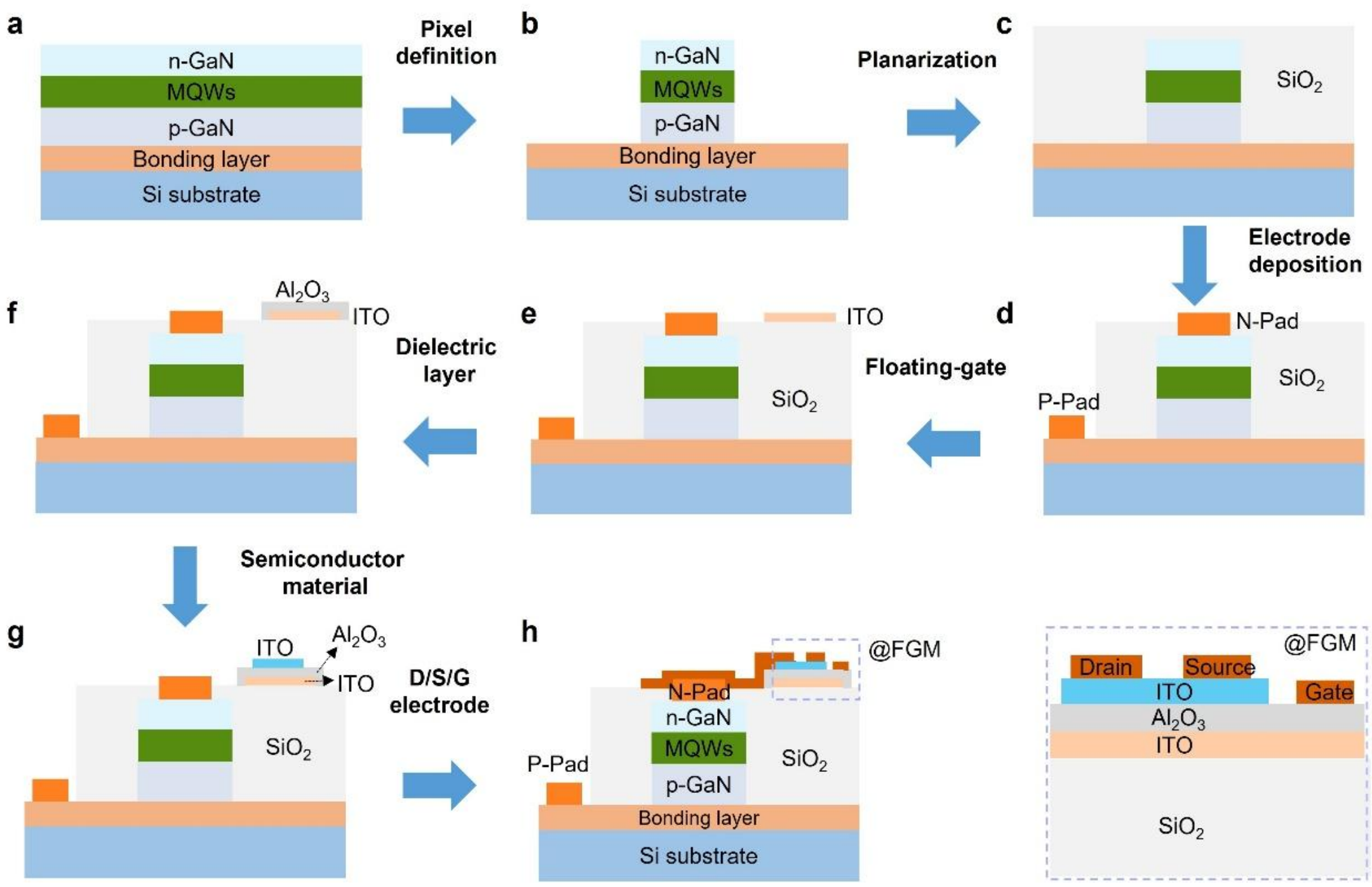

Supplementary Figure 2. Fabrication process of Micro-LED pixels integrated with ITO-based floating gate memories (FGM devices). a, Blue and green wafer structure after removal of the original growth substrate and buffer layer, with indium content in MQWs of 15% and 25% respectively, where the bonding layer consisted of 700 nm thick Cr/Pt/ Au metal stacks. **b**, Fabrication of 7.5-µm Micro-LED mesas using standard photolithography and dry etching. **c**, Deposition of 2.1-µm SiO_2 by plasma-enhanced chemical vapor deposition PECVD, followed by chemical mechanical polishing (CMP) to planarize the surface and achieve an ultra-flat mesa top. **d**, Formation of interconnection vias by inductively coupled plasma ICP etching (CF_4 plasma), followed by deposition of electrode metal (50 nm Ti/150 nm Au) via electron-beam evaporation (EBE). **e**, Deposition of ~6-nm ITO as the charge-trap layer by sputtering, patterned by photolithography and wet etching ($FeCl_3$/HCl solution). **f**, Deposition of ~18-nm Al_2O_3 dielectric layer by atomic layer deposition (ALD) under vacuum conditions at 250 °C, causing the properties of ITO transited from semiconducting to metallic. **g**, Vertical stacking of 6-nm semiconductor material onto the charge-trap layer by sputtering. **h**, Opening of electrode windows by ICP etching (Ar plasma), followed by simultaneous deposition of D/S/G electrodes and interconnect metal layers of 5 nm/50 nm Cr/Au by EBE. **i**, Schematic diagram showing layer configurations in the fabrication process.

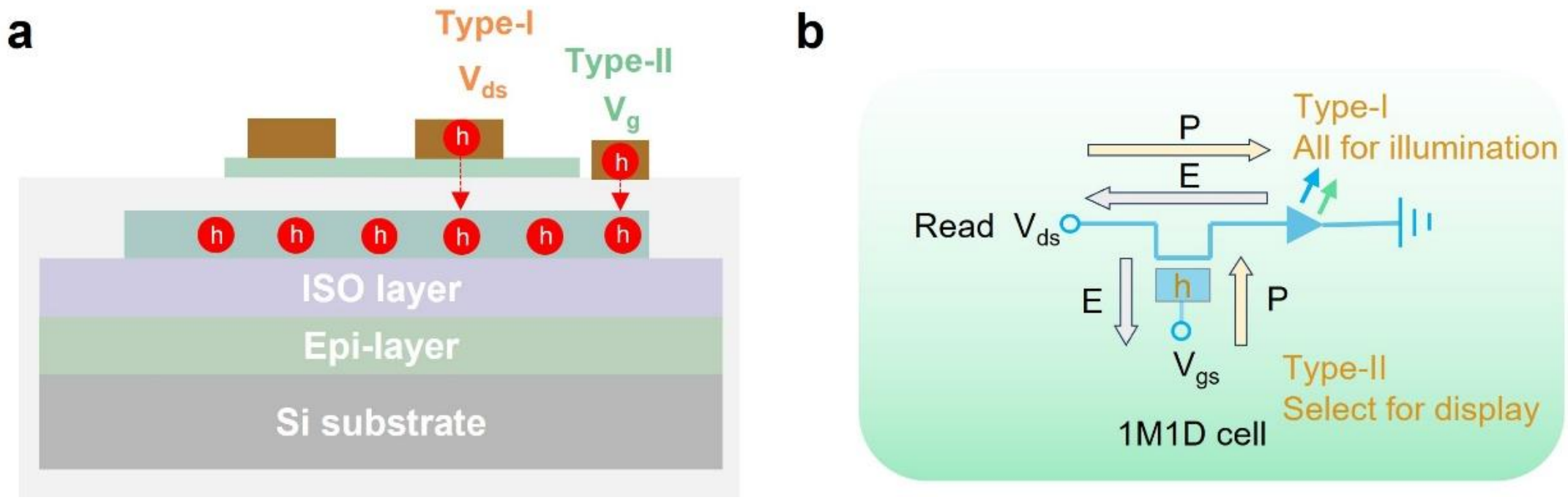

Supplementary Figure 3. Working mechanisms of the Integrated Nonvolatile Display-Illumination System (INDIS). a, Cross-sectional schematic of the FGM fabricated on a GaN wafer, where the two voltage injection ports enable distinct modulation modes. **b**, Illustration of the pixel circuit operation, showing two- and three-terminal modulation modes, which can be adapted to different application scenarios corresponding to the lighting modes of Micro-LEDs.

Supplementary Note 1. The operation principle of different drive modes and their potential application.

As shown in Supplementary Figure 3a, holes can be injected into the electron-capture layer by applying voltage pulses either to the drain electrode (common anode) or to the gate electrode, enabling dual-mode operation that underpins the Integrated Nonvolatile Display-Illumination System. As illustrated in Supplementary Figure 3b, when a large positive voltage is applied to the common anode (V_{ds}), holes are injected and stored in all charge-trapping layers, thereby increasing the carrier density in the channel region. This corresponds to type-I operation, where applying a subsequent read voltage to the anode turns on all Micro-LEDs simultaneously, providing high-brightness illumination for lighting scenarios. In contrast, when a positive pulse is applied to a specific gate electrode (V_g), charge is selectively stored in the corresponding trapping layers, activating only the targeted Micro-LED. This corresponds to type-II operation, which enables pixel-level pattern control suitable for display applications. Such dual-mode functionality allows the system to simultaneously support both display and lighting, thereby minimizing footprint and reducing overall complexity. For example, it can dynamically project warning symbols, pedestrian indicators, or navigation arrows onto the road surface while maintaining sufficient ambient illumination, thereby facilitating real-time information exchange between the vehicle and its

surroundings. Furthermore, its pixel-level programmability allows for adaptive lighting strategies tailored to diverse traffic scenarios (e.g., urban streets versus highways), effectively balancing visibility requirements and energy efficiency.

The INDIS system enables flexible switching between full-field illumination and high-resolution patterned display under complex driving environments. For instance, it can project warning signs, pedestrian indicators, or navigation arrows onto the road while maintaining sufficient ambient illumination, thereby facilitating real-time information exchange between the vehicle and its surroundings. Furthermore, its pixel-level programmability allows for adaptive lighting strategies tailored to diverse traffic scenarios (e.g., urban streets versus highways), effectively balancing visibility requirements and energy efficiency.

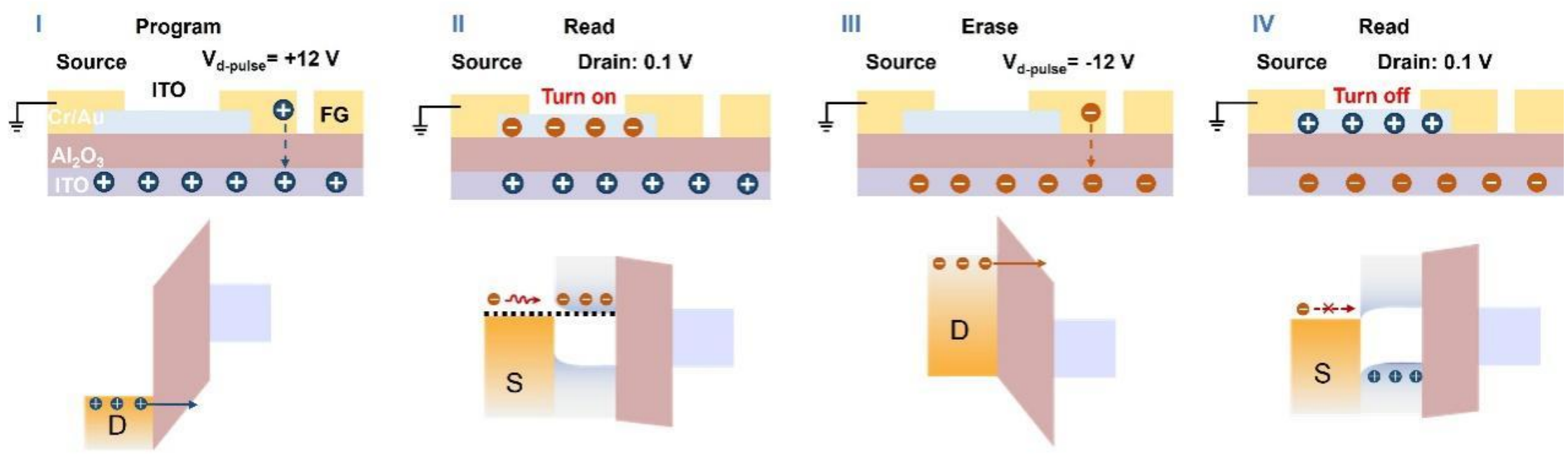

Supplementary Figure 4. Schematic illustration of the memory operation and corresponding energy band diagrams in type-I operation mode, including the hole programming state (I), read-after-program state (II), erase state (III), and read-after-erase state (IV).

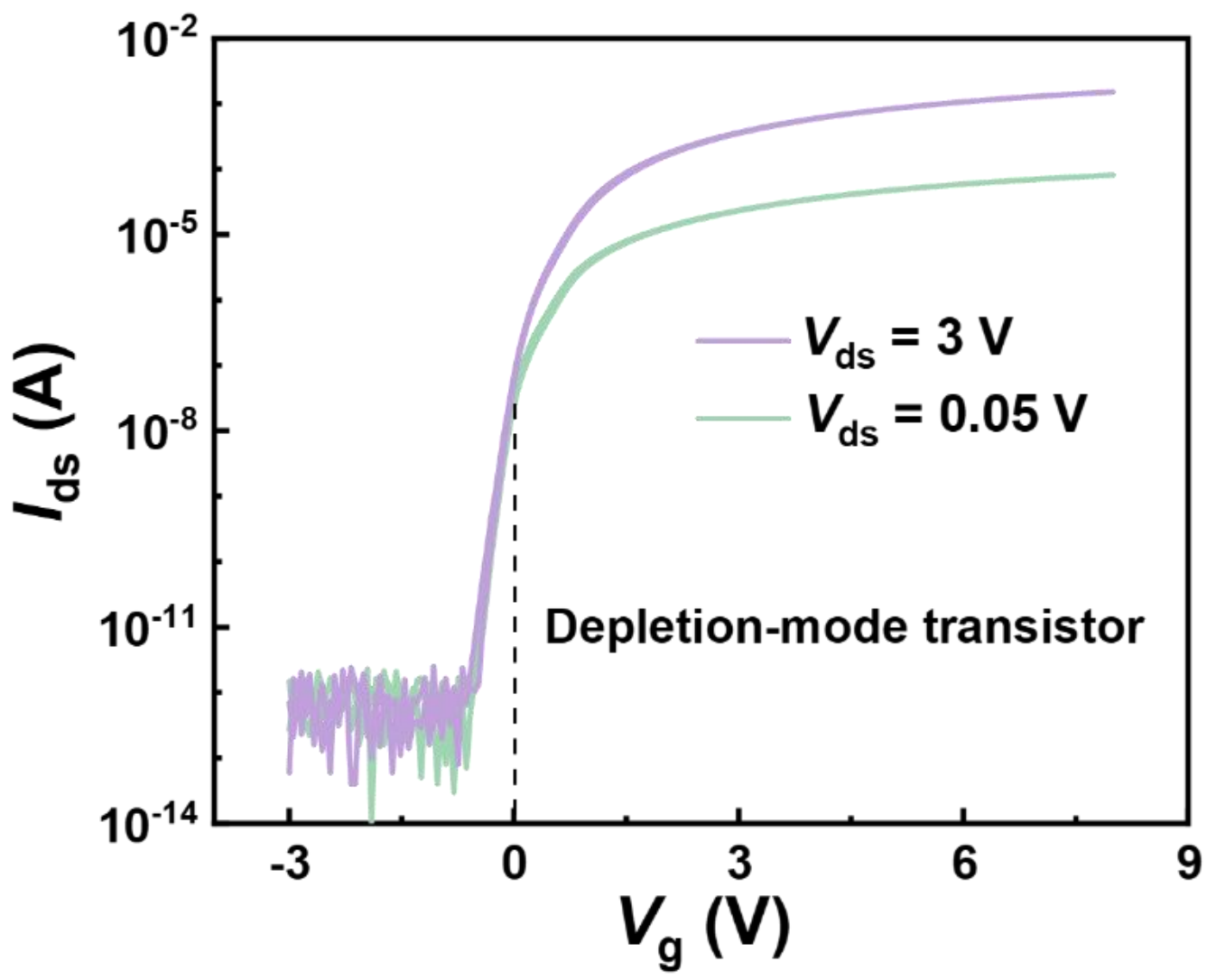

Supplementary Figure 5. Transfer characteristics of an ITO-based TFT (L = 1 µm, W = 16 µm) with voltage double sweeping range from -3 V to 8 V.

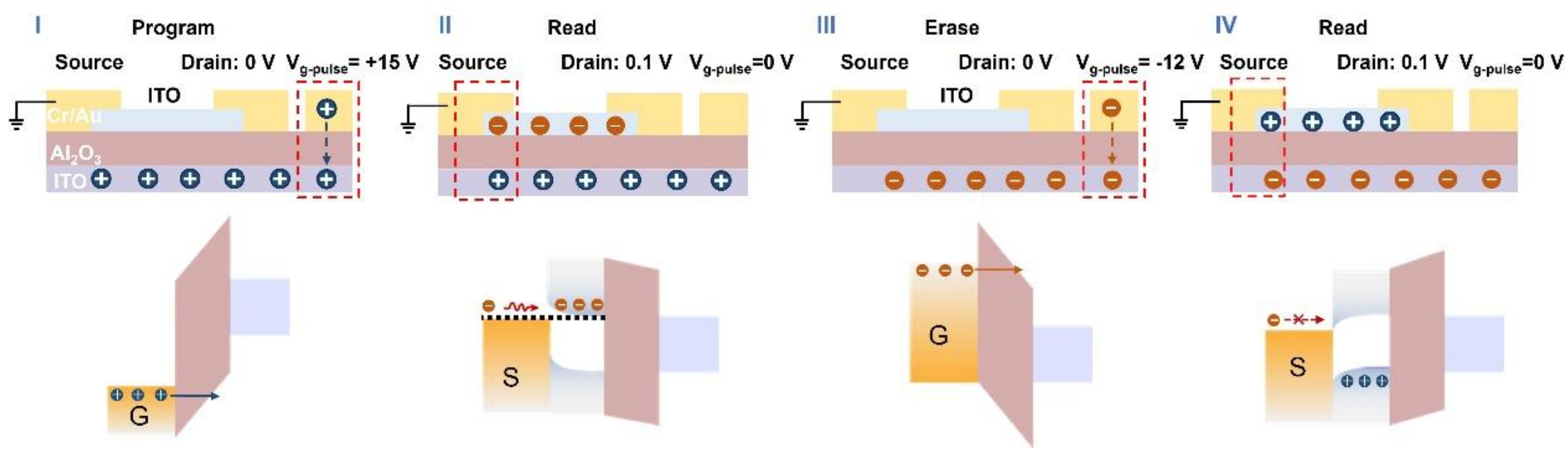

Supplementary Figure 6. Schematic illustration of the memory operation process and corresponding energy band diagrams in type-II operation mode, including the programming state (I), read-after-program state (II), erase state (III), and read-after-erase state (IV).

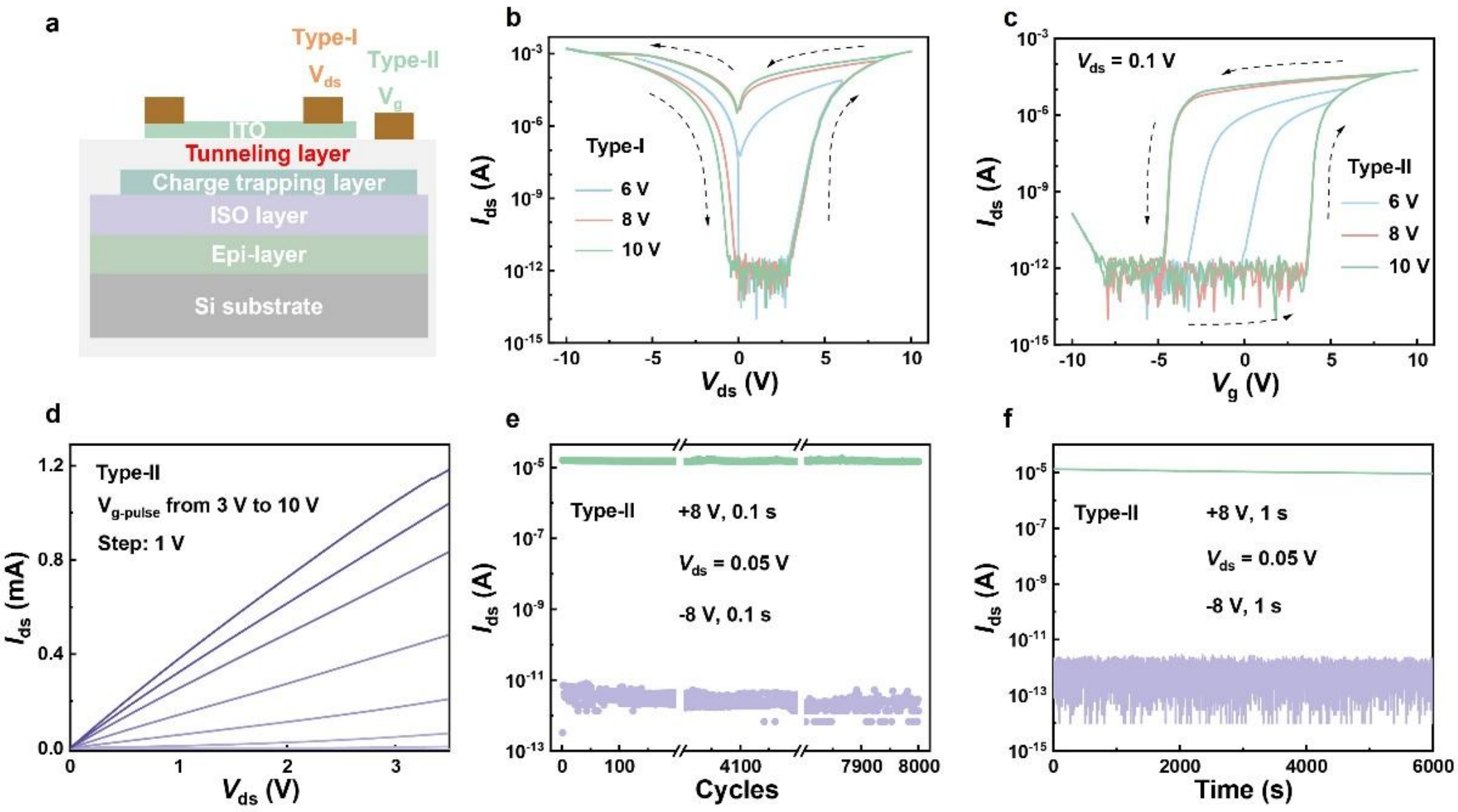

Supplementary Figure 7. The performance of FGM devices with thinner tunneling layer (10 nm). a, Cross-sectional schematic of the FGM fabricated on a GaN wafer. **b,c,** Sweep transfer characteristics of the device controlled by drain pulse voltage from -10 V to +10 V with type-I modulation (**b**), and type-II modulation (**c**). **d,** The IV curve of the device controlled by the pulse voltage forwarded on the gate, all pulse width is one hundred milliseconds. **e,f,** Endurance (**e**) and retention (**f**) performance in type-II operation.

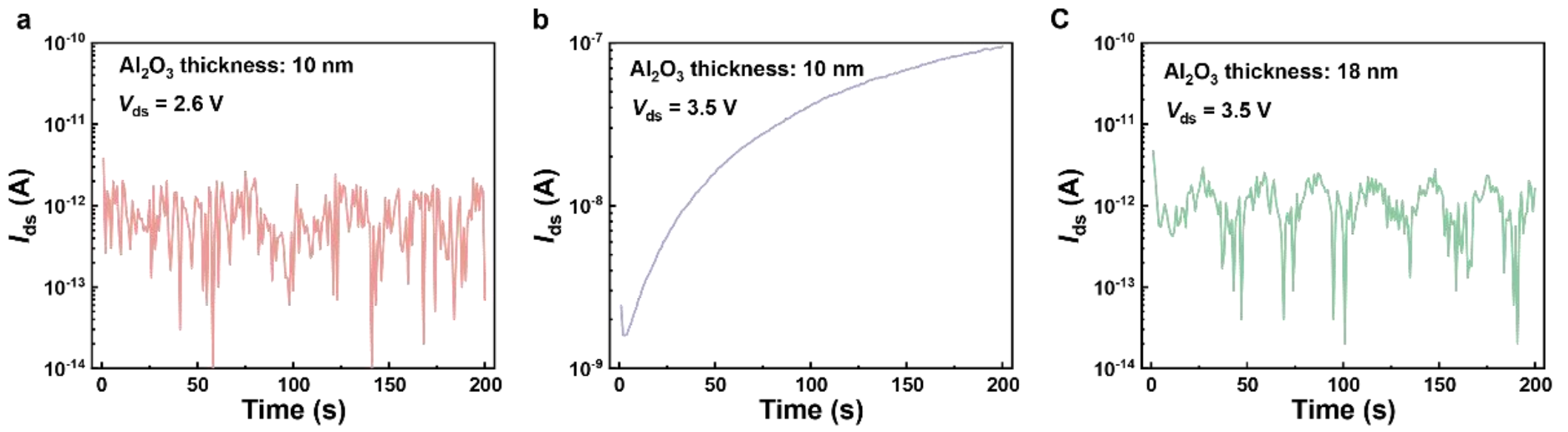

Supplementary Figure 8. Current stability for FGM devices with different drain bias applied. a,b, Current variation of FGM devices using a 10-nm Al_2O_3 tunneling dielectric layer at V_{ds} = 2.6 V (**a**) and V_{ds} = 3.5 V (**b**) during continuous operation. **c, C**urrent variation of FGM devices using an 18-nm Al_2O_3 tunneling dielectric layer at V_{ds} = 3.5 V.

Supplementary Note 2. The performance of FGM devices with thinner tunneling layer.

To explore lower-voltage operation, standalone FGM devices is fabricated with thinner Al_2O_3 tunneling layer (10 nm). As shown in Supplementary Figure 7, these devices exhibit large memory window of 3.48 V along with on/off current ratio exceeding 10^7 within a voltage sweep range of -8 V to +8 V in type-I operation mode. in type-II operation mode, the transfer characteristics show pronounced hysteresis with a memory window of 8.3 V and an on/off current ratio exceeding 10^8, while maintaining a positive threshold voltage around 3.5 V. The output characteristics under gate pulse programming (100 ms pulse width) show a maximum current of ~1.2 mA, indicating robust driving capability. Furthermore, endurance testing up to 8,000 program–read–erase–read cycles (±8 V pulse, 0.1 s; read at 0.1 V) demonstrates that the read current remains stable, while preserving a consistent on/off ratio. A retention time of 6000 s was characterize, with the on/off current ratio maintained above 10^8. Notably, the on-state current exhibits only a slight decrease from 13.4 μA to 9.2 μA, demonstrating excellent data retention capability and device stability. However, as the drain voltage increases, the retention performance degrades significantly. As shown in Supplementary Figure 8, for FGM devices with a thinner tunneling layer, a gradual increase in the output current is clearly observed when the drain voltage exceeds 3.5 V. This behavior indicates that devices with thinner dielectric layers are more suitable for low-brightness operating conditions.

By contrast, such current drift is absent in FGM devices with an 18 nm-thick tunneling layer, corresponding to the device used in this work, highlighting its superior stability under higher operating voltages. Looking forward, further reduction of the operating voltage while maintaining robust nonvolatile storage will depend on the development of high-permittivity dielectric materials, which can ensure sufficient charge confinement even at reduced thickness. This strategy is expected to enable an improved balance between energy efficiency and storage performance in next-generation FGM-integrated display systems.

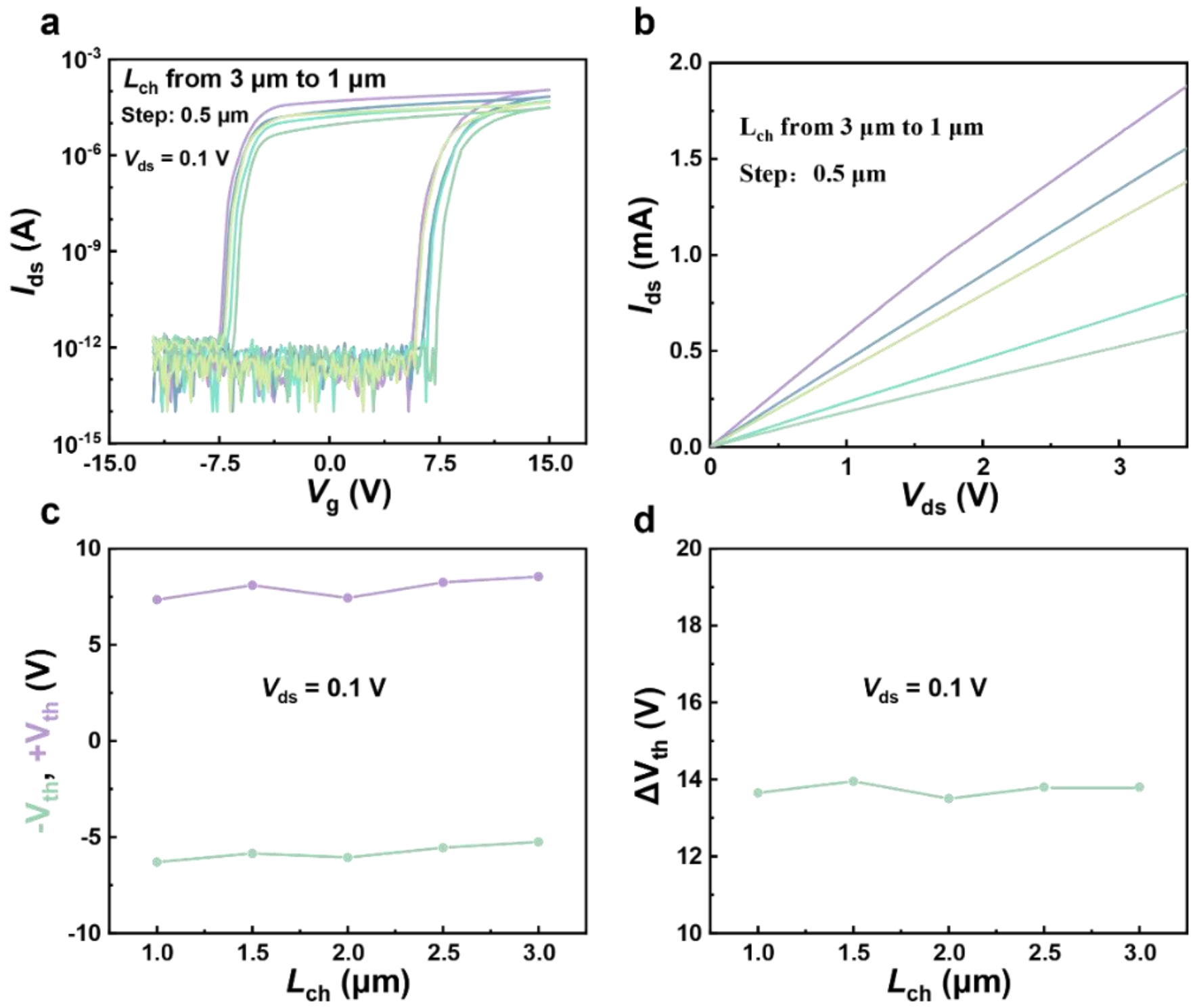

Supplementary Figure 9. Electrical characteristics of FGM devices with different channel length in type-II operation. **a**, Transfer characteristics of the FGM devices with gate voltage sweeping from -12 V to +15 V. **b**, Output characteristics of the FGM devices after +15 V gate pulse voltage. Pulse width: 0.1 s. **c**,**d**, Threshold voltage ($\pm V_{th}$) distribution (**c**) and memory window (**d**) extracted from Supplementary Figure 5a.

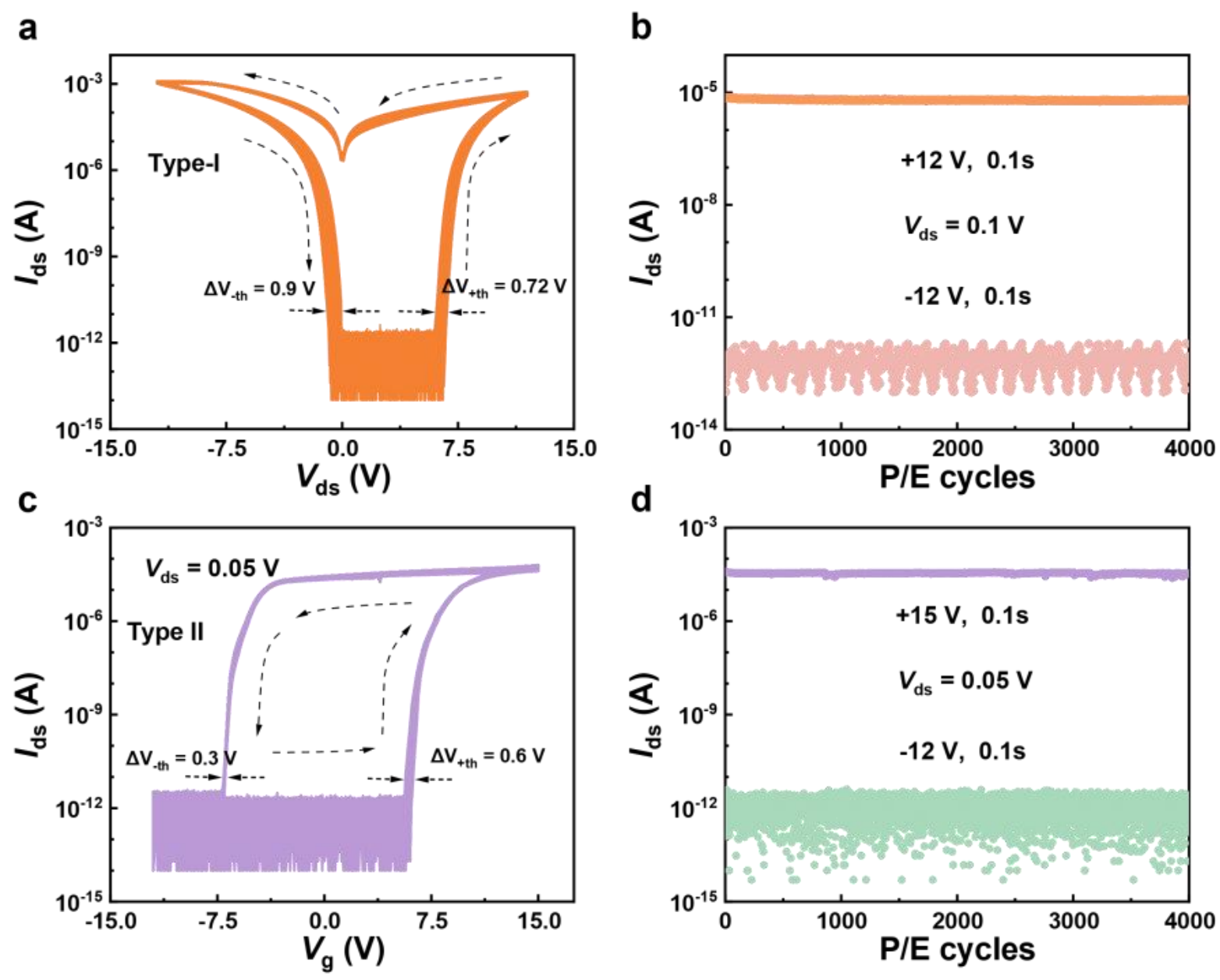

Supplementary Figure 10. Electrical stability and endurance of the FGM device. a,b, Transfer characteristics of a FGM device measured over 200 consecutive cycles in type-I (**a**) and type-II (**b**) operation mode, indicating stable operation. **c,d,** Endurance performance in type-I (**c**) and type-II (**d**) operation mode, evidencing robust switching over repeated cycling.

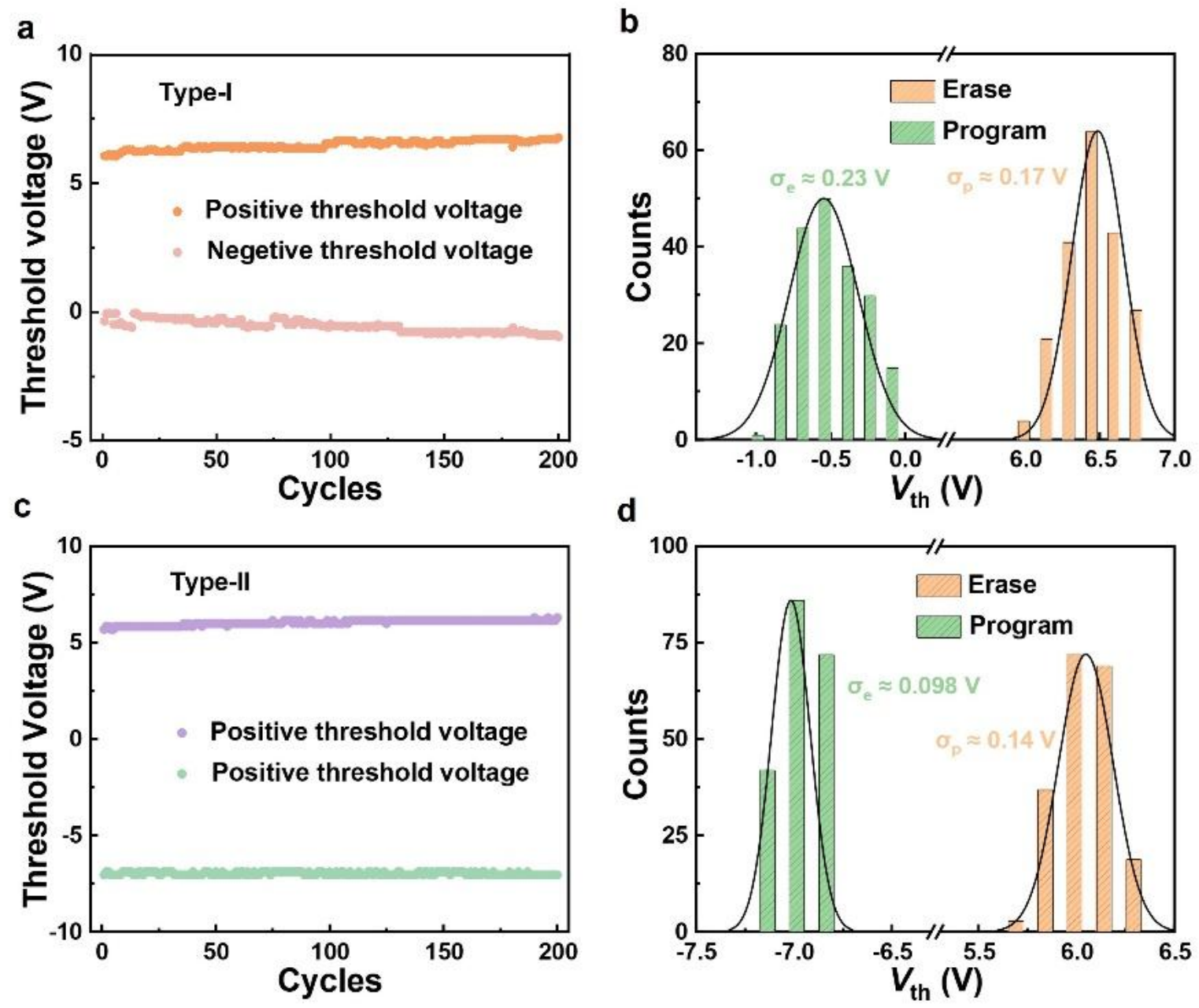

Supplementary Figure 11. Threshold voltage stability of the FGM device. Threshold voltage variation (a) and its statistical distribution (b) extracted from Supplementary Figure 10a in type-I mode. Threshold voltage variation (c) and distribution (d) extracted from Supplementary Figure 10c in type-II mode.

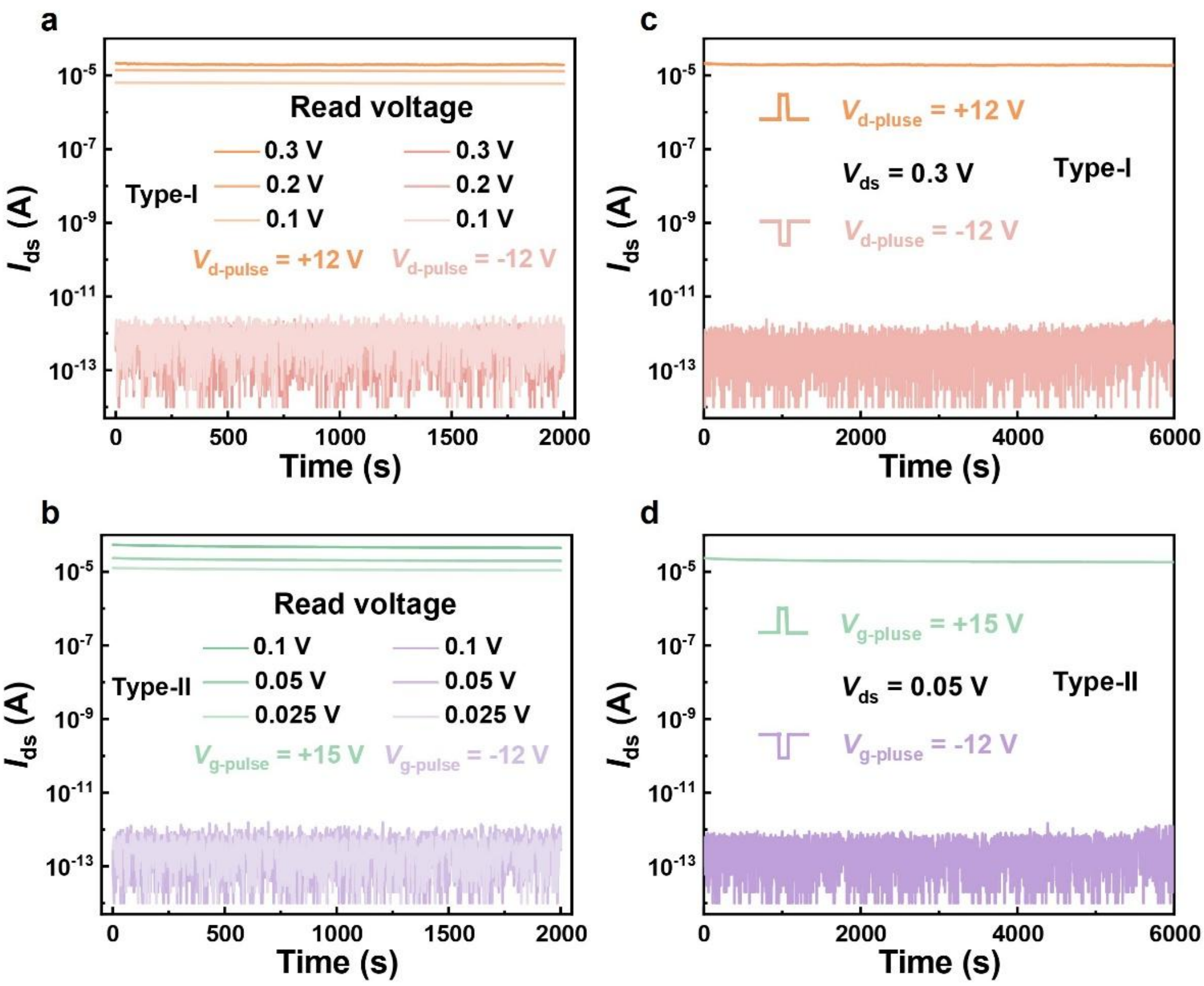

Supplementary Figure 12. Retention performance of FGM devices. Retention characteristics over 2000 s under different read voltages for type-I (a) and type-II (b) operation modes, respectively. Long-term retention characteristics measured at a read voltage of 0.3 V for type-I mode (c) and 0.05 V for type-II mode (d).

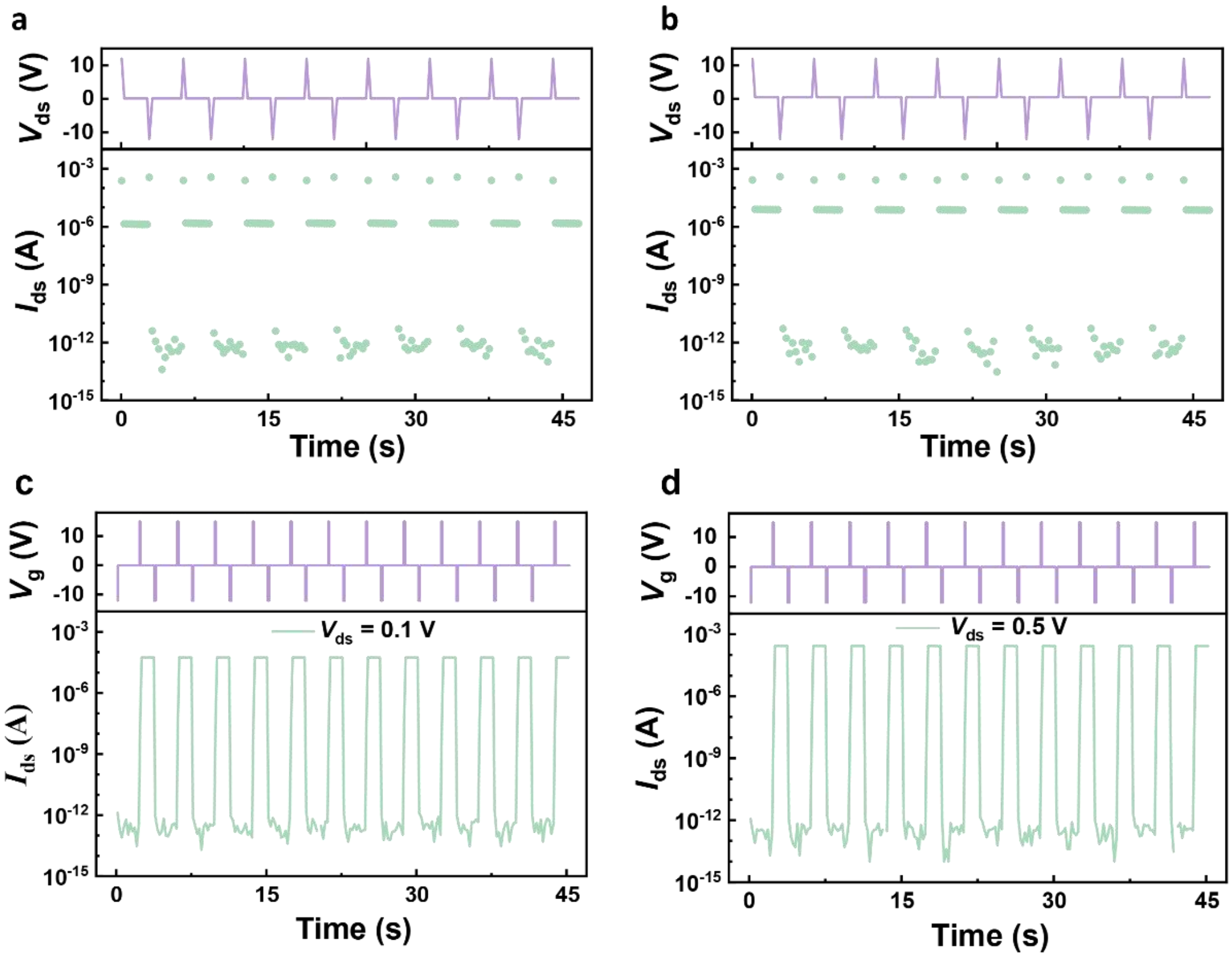

Supplementary Figure 13. Switching performance of FGM devices under different modulation modes. **a**,**b**, type-I operation: repeated program/read/erase/read sequences by alternating drain voltage pulses. Program and erase voltages are +12 V and –12 V, respectively, with a pulse width of 0.1 s; read voltages were 0.1 V and 0.5 V, respectively. **c**,**d**, type-II operation: program and erase processes applied via the gate electrode (+15 V/–12 V, 0.1 s). Read voltages are 0.1 V and 0.5 V, respectively.

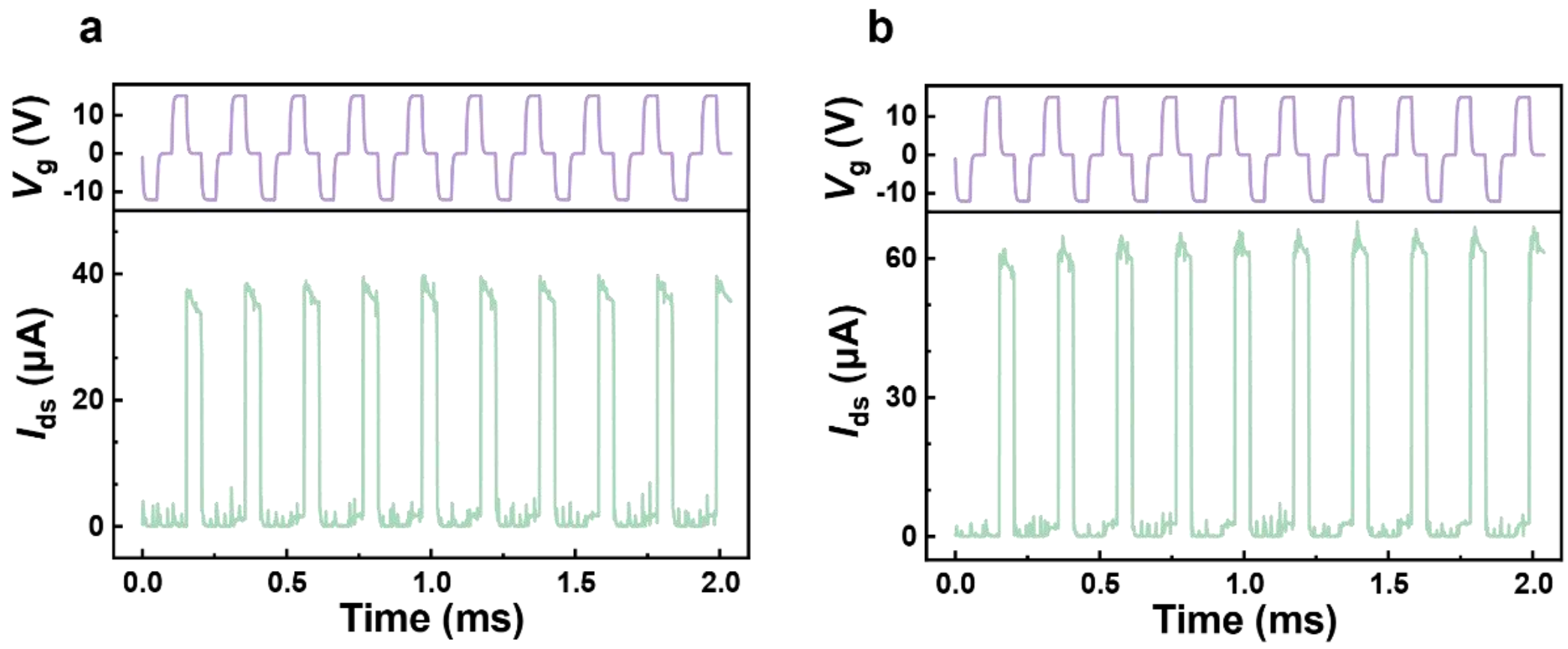

Supplementary Figure 14. Demonstration of high-frequency operation in type-II mode by sequential programming (+15 V) and erasing (-12 V) pulses with ~50 μs intervals. a, The standalone FGM. **b**, The integrated 1M1D. And the read voltage applied between the drain and source are 0.5 V and 3 V, respectively**.**

The voltage rise time and fall time of ~11 μs and ~10 μs are obtained, respectively. It indicates a total time of ~200 μs per frame, corresponding to an effective refresh rate of approximately 5 kHz.

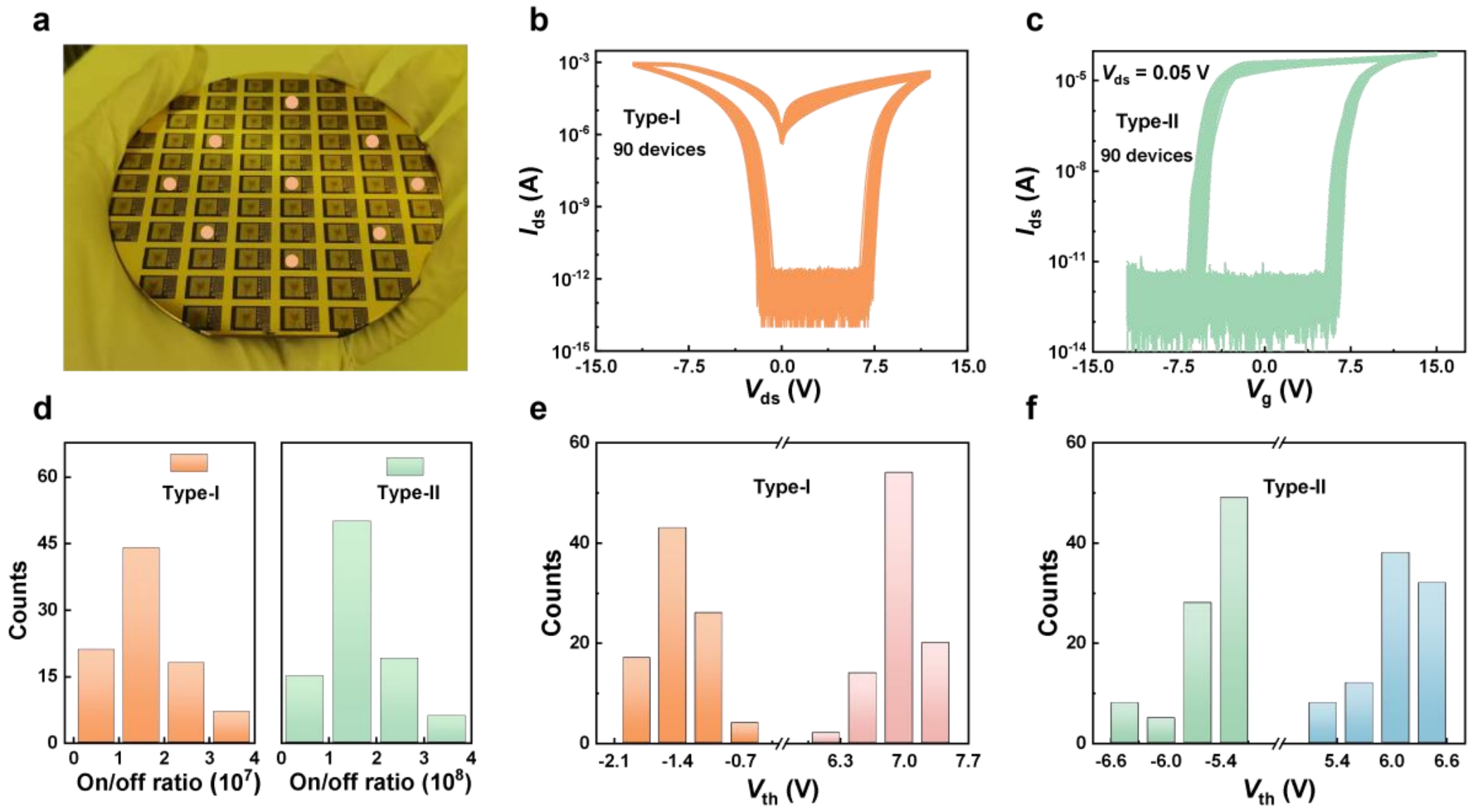

Supplementary Figure 15. Uniformity performance of FGM devices on a 4-inch wafer. a, Schematic illustration of the sample locations across the 4-inch wafer. Summary of transfer characteristic curves obtained from 90 devices with type-I mode (**b**) and type-II mode (**c**). d, Statistical distribution of the on/off current ratios under both modes. Statistical distributions of positive and negative threshold voltage under two modes with type-I mode (**e**) and type-II mode (**f**).

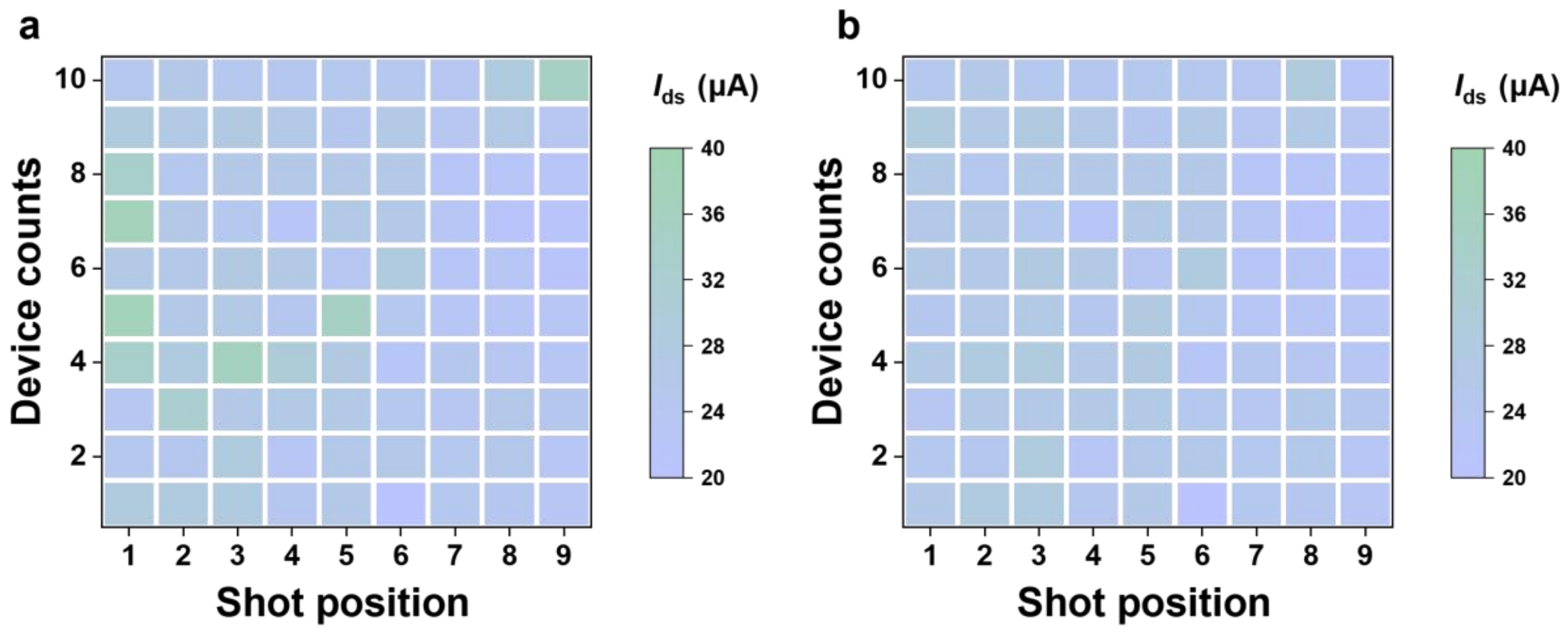

Supplementary Figure 16. Current uniformity accross the 4-inch wafer. a, Current distribution of the devices selected from Supplementary Figure 15a after programming (+15 V, 0.1 s). **b,** Spatial current distribution of high-current devices after reducing the corresponding programming voltage of the device, demonstrating improved uniformity and reduced standard deviation.

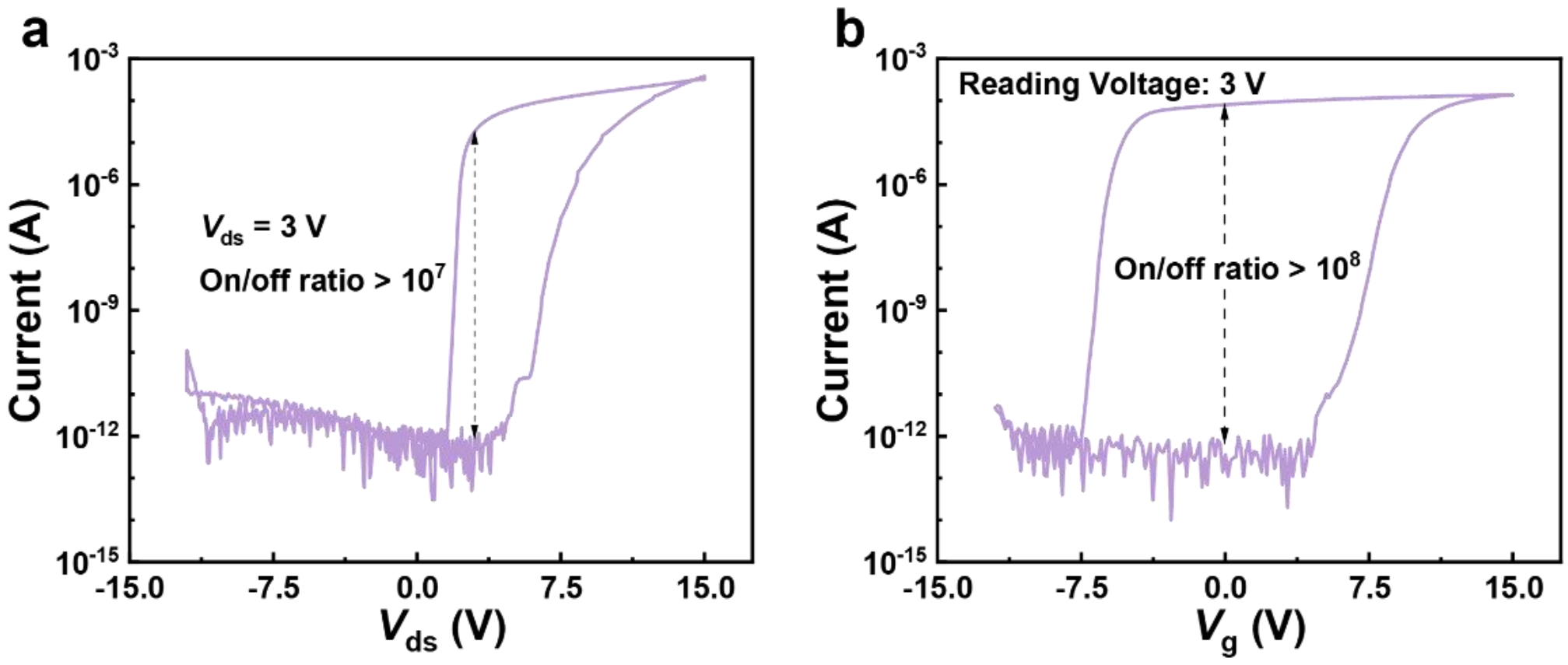

Supplementary Figure 17. Memory window of 1M1D pixel with different modulation modes. a, The I–V characteristic of integrated device in type-I modulation mode, exhibiting a clearly memory window and a high on/off current over 10^7 at V_{ds} = 3 V. **b**, in type-II, transfer characteristics of the integrated device with gate voltage sweeping from -12 V to +15 V, showcasing a large and stable window of 12.8 V and a high on/off current over 10^8, with reading voltage of 3 V.

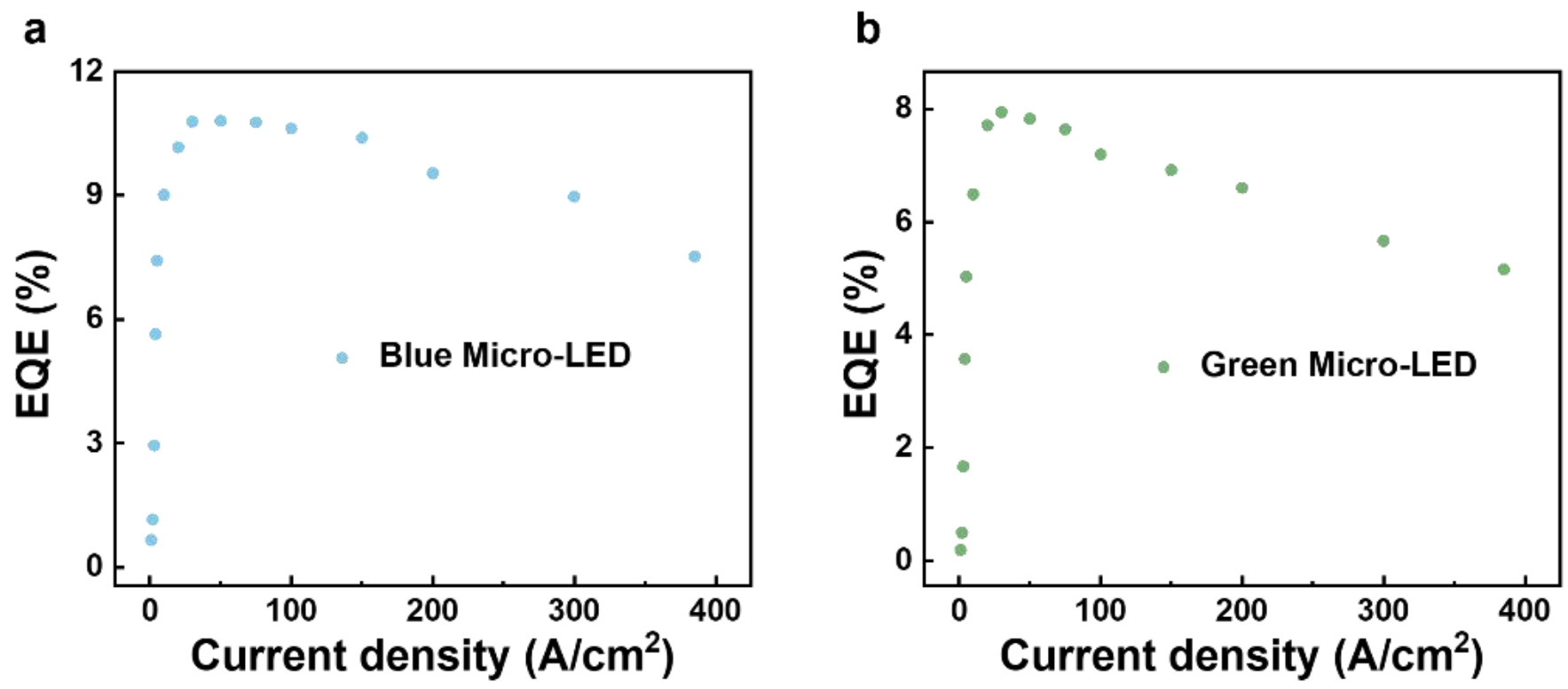

Supplementary Figure 18. External quantum efficiency (EQE) characteristics of the Micro-LED. Blue (**a**) and green (**b**), respectively.

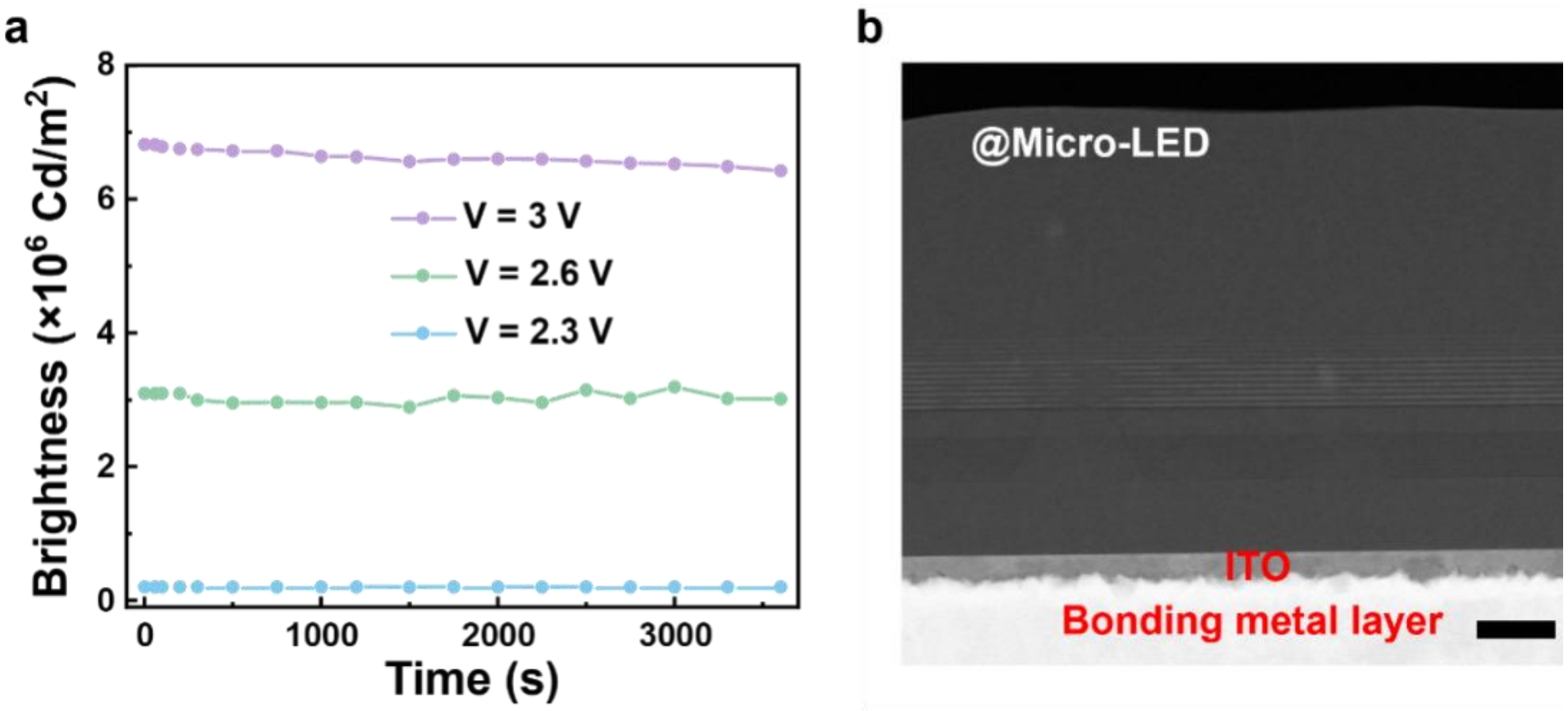

Supplementary Figure 19. Brightness modulation and structural characterization of Micro-LEDs. a, The variation of Micro-LED brightness lit up under different conditions. **b,** The cross-section of Micro-LED epitaxy layers. Scale bars, 500 nm.

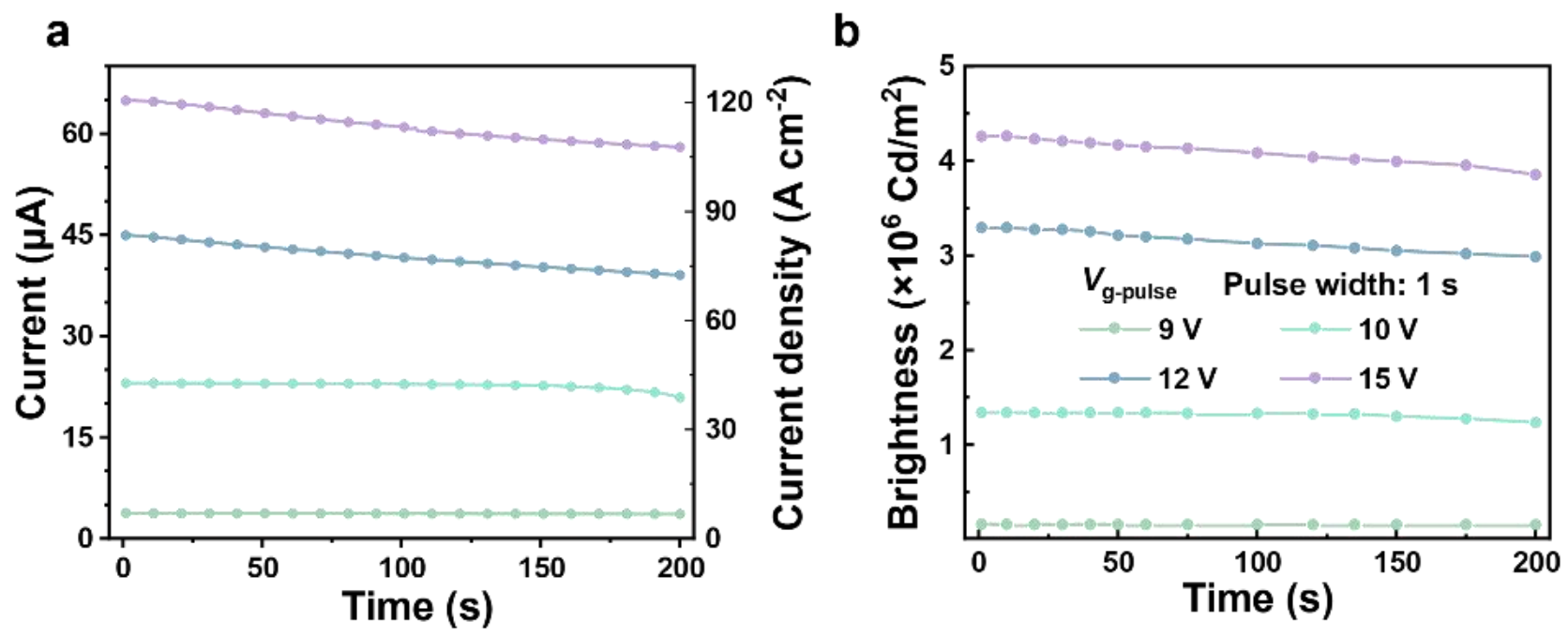

Supplementary Figure 20. Current (a) and brightness (b) retention characteristics of the 1M1D under different gate voltages. To further evaluate the reliability of the retention analysis, quantitative results of the luminance evolution during the retention process are presented in Supplementary Figure 20. The results show that, under different gate pulse voltages (9 V, 10 V, 12 V and 15V; pulse width of 1 s), the brightness retention remains at 96.87%, 92.27%, 90.62%, and 90.51%, respectively, after 200 s. These results are consistent with the electrical retention behavior of the FGM device and further confirm the stable light output of the integrated system.

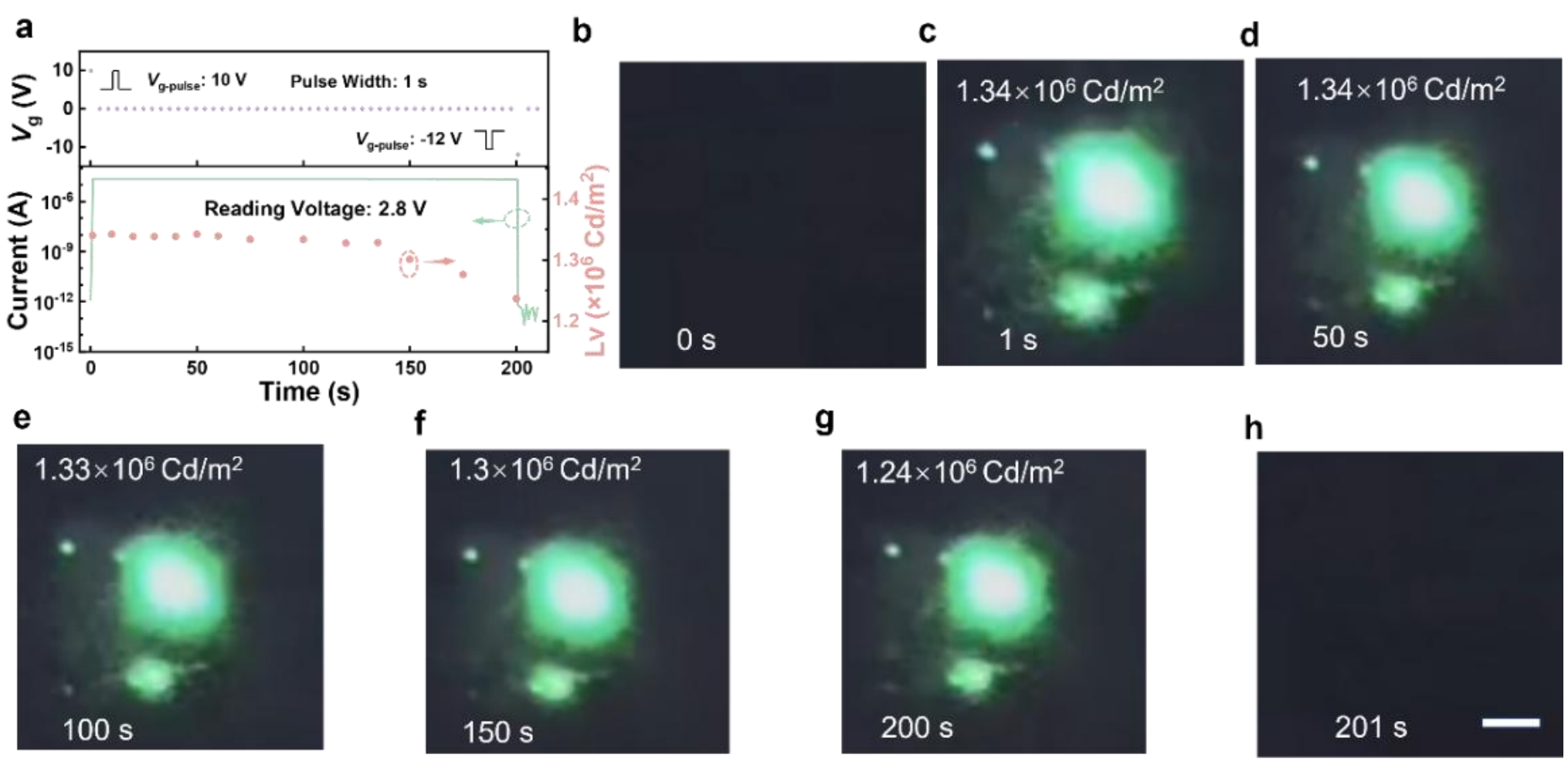

Supplementary Figure 21. Retention characteristics of the 1M1D pixel acquired from Supplementary video 1. a, Time-dependent variation of current and brightness after a pulse voltage (10 V, 1 s) applied onto the gate, with the reading voltage of 2.8 V. The results indicate negligible variation in driving current after 200 s. **b**, Initial optical image of Micro-LED before gate voltage pulse. **c,** The image of Micro-LED after a positive pulse voltage applied onto the gate. **d-g**, The images of Micro-LED after a certain duration following the removal of gate voltage, corresponding to 50 s (**d**), 90 s (**e**), 150 s (**f**) and 200 s (**g**). **f**, The image of Micro-LED after a negative pulse voltage applied onto the gate. Scale bar, 10 μm. The results indicate excellent brightness retention.

Supplementary Note 3. The discussion of heat buildups under high-brightness conditions.

The EQE of the standalone Micro-LEDs has been measured and is now included in the Supplementary Figure 18. At a current density of 385.7 A·cm^{-2}, the EQE values are 7.52% (blue) and 5.16% (green), showing moderate reductions compared to their peak values (10.78% and 7.94%, respectively), indicating that efficiency droop remains within an acceptable range. It can be contributed to improved thermal dissipation, with the implementation of metallic interconnects on the bottom side, which help to reduce the heat accumulation and Auger non-radiative recombination.

Furthermore, to evaluate the thermal stability, Supplementary Figure 19 displays long-term continuous operation tests carried out under different brightness conditions. When operated at a luminance of 6.81 × 10^{6} Cd·m^{-2} (3 V bias), the device exhibits only ~5.76% luminance degradation after 3.6 × 10^{3} s of continuous operation. And there are almost no degradation under lower brightness conditions (3 × 10^{6} Cd·m^{-2} (2.6 V bias), 0.2 × 10^{6} cd·m^{-2} (2.3 V bias)).

These results indicate that the device can sustain stable operation under high-brightness conditions without significant irreversible degradation, demonstrating effective thermal management enabled by the device structure, where heat generated from the chip is timely conducted away via the bottom bonding metal.

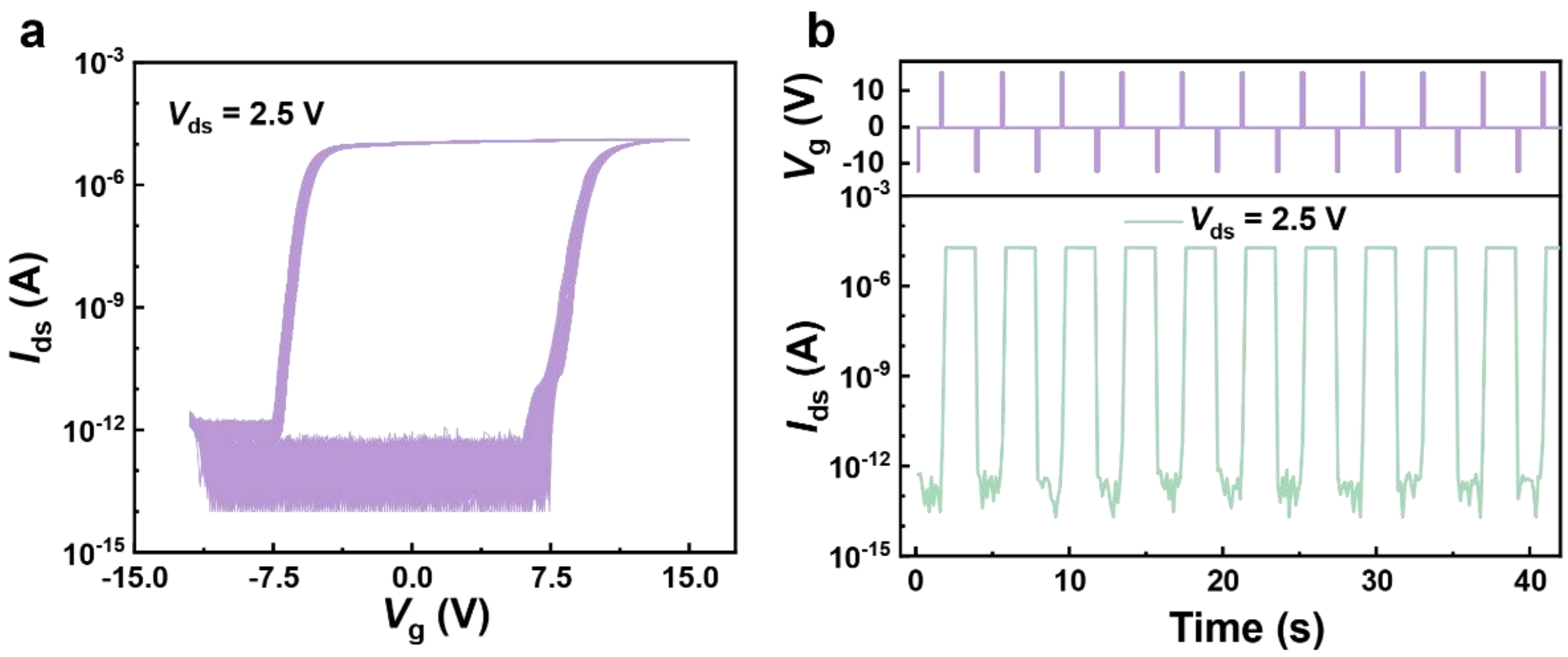

Supplementary Figure 22. Stability and switching performance of 1M1D pixel in type-II operation. a, The transfer characteristics of the integrated device under 200 continuity cycle tests, indicting outstanding stability. **b**, Switching curves of the integrated device with reading voltage of 2.5 V and gate pulse width of 0.1s, respectively.

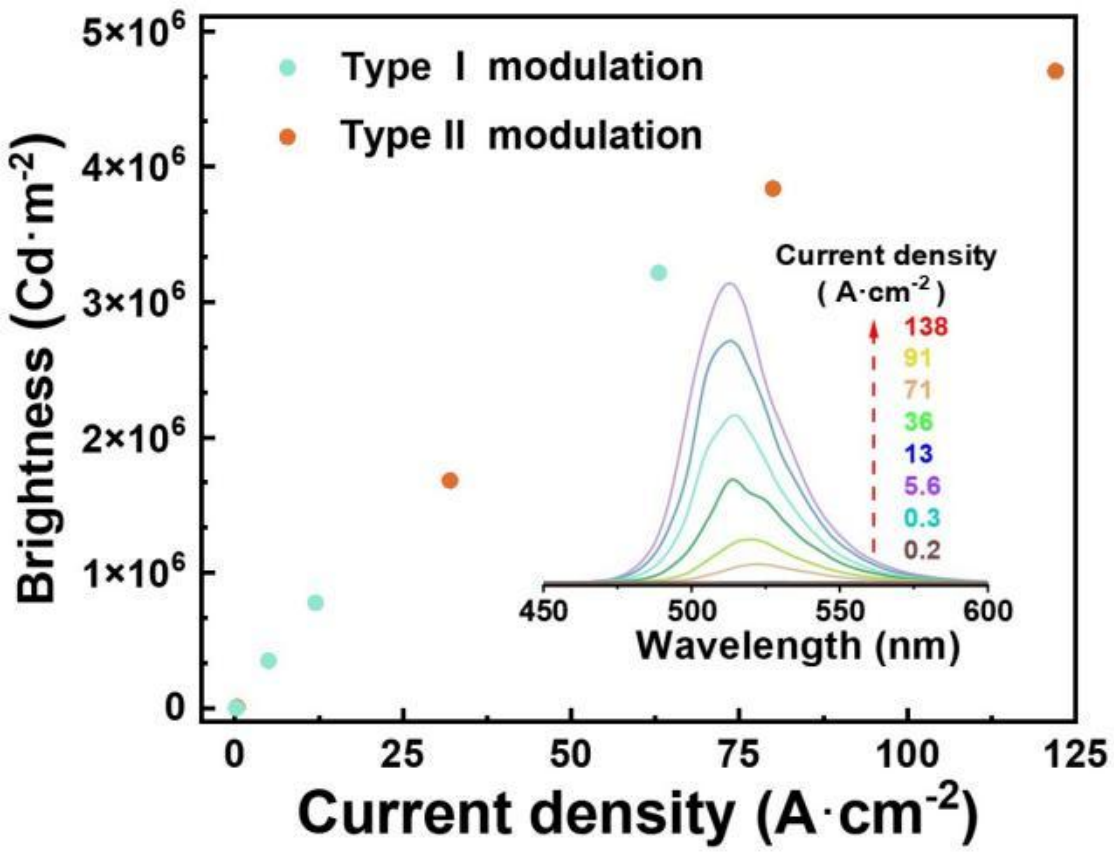

Supplementary Figure 23. Brightness modulation of 1M1D pixel in type-I and type-II modes, where inset displays the EL spectra at the corresponding current densities.

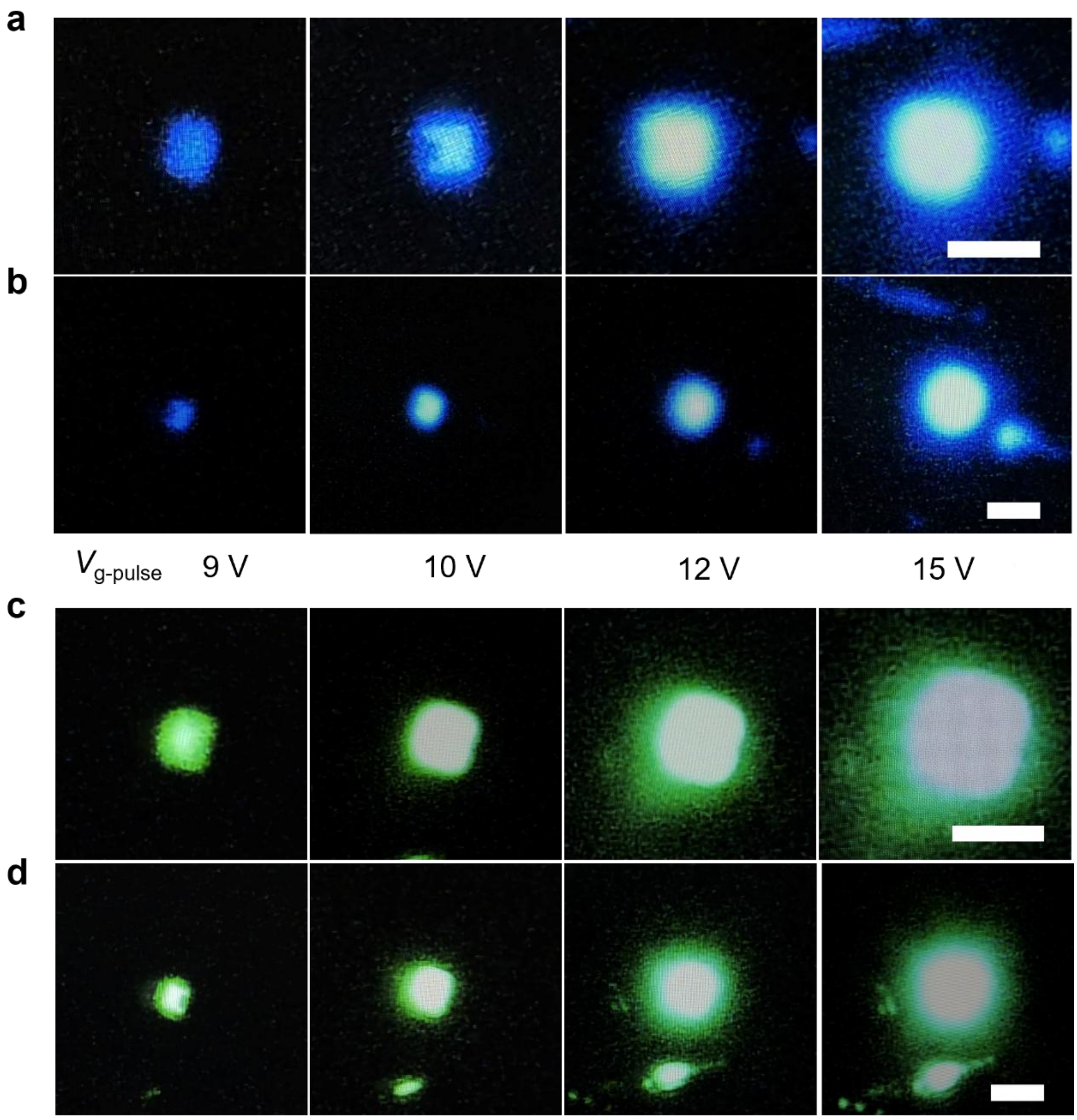

Supplementary Figure 24. Brightness control of the FGM integrated Micro-LED with different sizes. a,**b**, Blue Micro-LEDs with pixel sizes of 30 μm and 15 μm. **c**,**d**, Green Micro-LEDs with pixel sizes of 30 μm and 15 μm. Scale bars, 60 μm and 30 μm for (**a**,**c**) and (**b**,**d**).

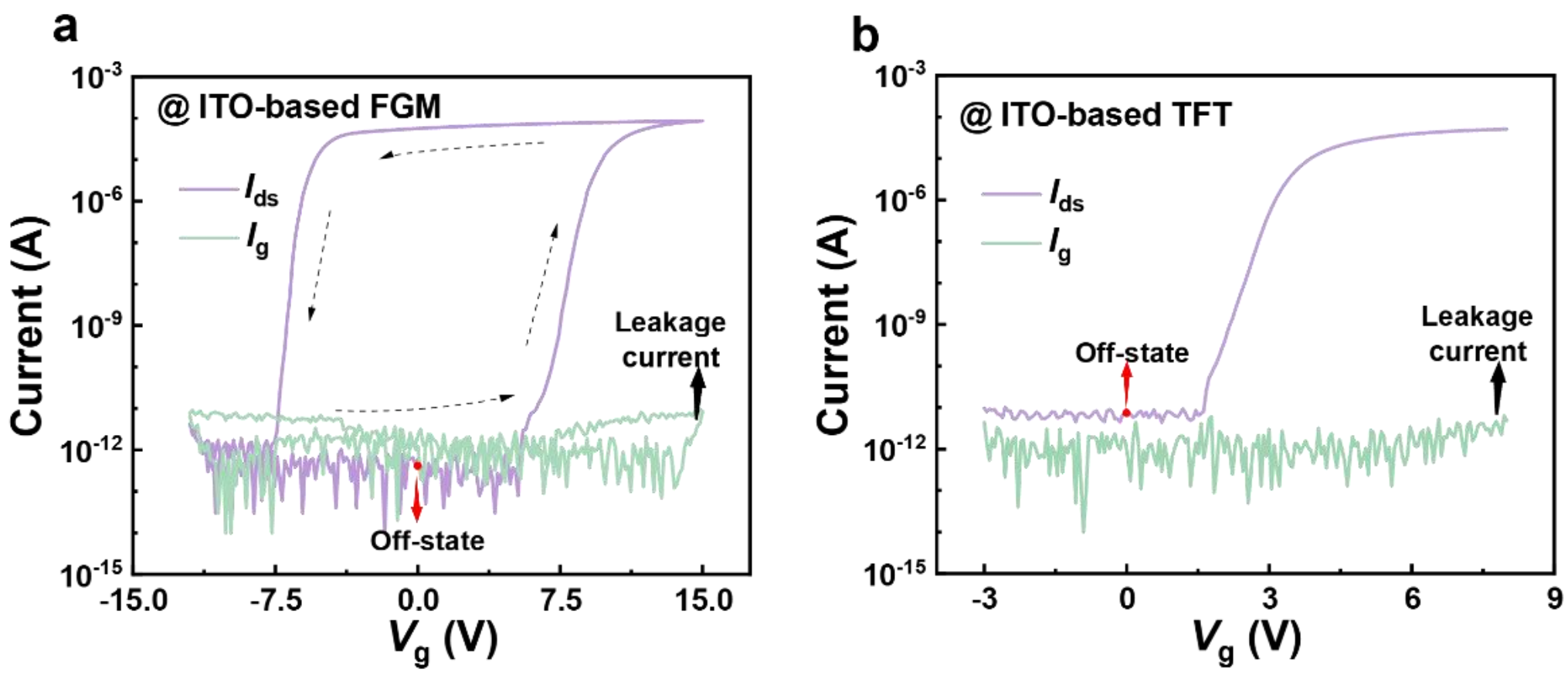

Supplementary Figure 25. Transfer performance of the Micro-LED device integrated with ITO-based TFT/FGM. a,**b**, The transfer curve of (a) the FGM-integrated Micro-LED pixel with gate voltage sweeping from -12 V to 15 V, and (b) Single sweep from -3 V to 8 V for TFT-integrated Micro-LED pixel with V_{ds} fixed at 2.8 V.

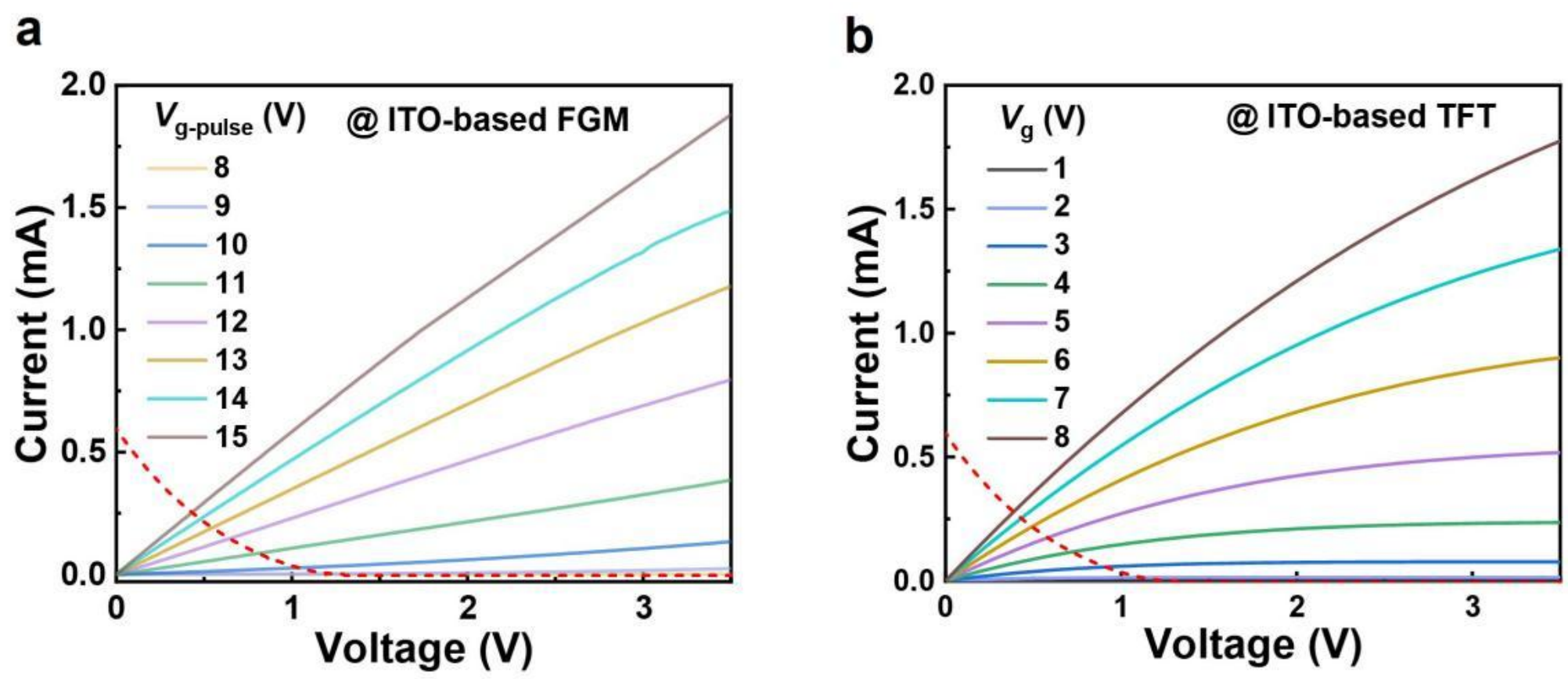

Supplementary Figure 26. Output performance of the Micro-LED device integrated with ITO-based TFT/FGM. a,b, The IV curve of the integrated device controlled by the gate pulse voltage (a), continuously gate supply (b). In **a**, all of the pulse width are 0.1 s.

Supplementary Note 4: The energy consumption analysis of 1M1D.

A significant portion of the power consumption in Micro-LED displays originates from the driving circuitry; therefore, we provide a detailed discussion on how different factors in the driving scheme affect the overall system power consumption.

(1) Dynamic power: the first component of power consumption arises from sustained gate-line scanning for image refresh.

For a normal TFT-drived Micro-LED displays, the power consumption can be calculated as follows:

$$W = |V_g| \times I_g \times \mathrm{t} \tag{1}$$

Where V_g, I_g and t are the gate voltage, gate leakage current and gate voltage duration.

For the FGM-drived Micro-LED displays, the power consumption can be calculated as follows:

$$W = |V_g| \times I_g \times \triangle \mathrm{t} \times \mathrm{n} \tag{2}$$

Where V_g, I_g, $\triangle$t and n are the gate voltage, gate leakage current and gate voltage pulse width and pulse number.

Assuming a Micro-LED is continuously lit for 5 minutes. For our developed FGM pixel driver circuit, as shown in Supplementary Figure 25a. when a 15 V pulse voltage applied onto gate electrode (pulse width 100 ms), the reading current and switching gate current were 51.2 μA and 7.61×10^{-12} A, corresponding to a switching energy of just 11.41 pJ. Since no continuous gate voltage is required to maintain the display state, the theoretical dynamic power consumption associated with refresh driving is reduced to nearly zero. For the ITO-based TFT pixel driver circuit, Supplementary Figure 25b indicates the transfer characteristics of the integrated device; in order to attain the same drive current with the developed FGM integrated device, an 8 V voltage should be applied to gate with a gate current of 5.33×10^{-12} A. At this condition, the dynamic power consumption is measured to be 11560 pJ after continuously lit for 5 minutes. For the reported MoS_2 based TFT with atomically thin channel, previous work[1] demonstrated that the leakage current could be as low as 6.41×10^{-13} A at a gate voltage of 6.2 V, corresponding to a dynamic power consumption is 1192 pJ after the Mico-LED remained continuously illuminated for 5 minutes. Conventional

low-temperature polycrystalline silicon (LTPS) TFTs typically exhibit a leakage current of 5×10^{-11} A with a gate voltage of approximately 8 V, resulting in a dynamical power consumption of ~1.2×10^{5} pJ[2,3]. It indicates that all of the above TFT-based devices consume much more energy than our developed FGM-integrated device, particularly under low-refresh-rate operation.

(2) Static power: the second component of total power consumption originates from the driver circuit in off-state when the Micro-LED is in the off state.

For our developed FGM pixel driver circuit, Supplementary Figure 25a depicts that the circuit current is as low as 5×10^{-13} A in off state. This ultralow leakage current results from the complete channel depletion ensured by the FGM structure and the wide bandgap of ITO, together yielding an extremely low static power of only 1.4×10^{-12} W at a bias voltage of 2.8 V. In contrast, for the ITO-based TFT pixel driver circuit, Supplementary Figure 25b indicates an off-state current of approximately 7.11×10^{-12} A, corresponding to a static power consumption per pixel of only 2×10^{-11} W at the same bias voltage, which is several times higher than that of the FGM-based circuit due to incomplete depletion channel (Supplementary Figure 5). Similarly, reported driver circuits based on depletion-mode MoS_2-TFT and LTPS-TFT also exhibit high static power[1-3], which are 1.4×10^{-11} W (off-state current: 5×10^{-12} A) and 3.3×10^{-11} W (off-state current:1.17×10^{-11} A), respectively.

(3) Driver loss: the third component of power consumption originates from the voltage dropped across the driver circuit when the Micro-LED is on.

As shown in Supplementary Figure 26, to more accurately evaluate the proportion of power consumed by the driver under operating conditions, the IV characteristics of both the Micro-LED and driver circuit are measured separately. The high carrier transport capability of ITO results in a low channel resistance and thus a high on-state current. The intersection point indicates that only 0.41 V is dropped across the ITO-based FGM, meaning that 88.29% of the total 3.5 V bias is applied to the Micro-LED, corresponding to a driver power loss of just 11.71% (Supplementary Figure 26a). For the ITO-based TFT pixel driver circuit (Supplementary Figure 26b), the power loss is similarly low at 11.43%. In comparison, previous studies[4,5] reported driver losses of 14.3% and 29.6% for MoS_2-based and

LTPS-based TFTs, respectively. Therefore, the developed ITO-based FGM-integrated nonvolatile display–illumination system demonstrates distinct advantage in terms of power consumption.

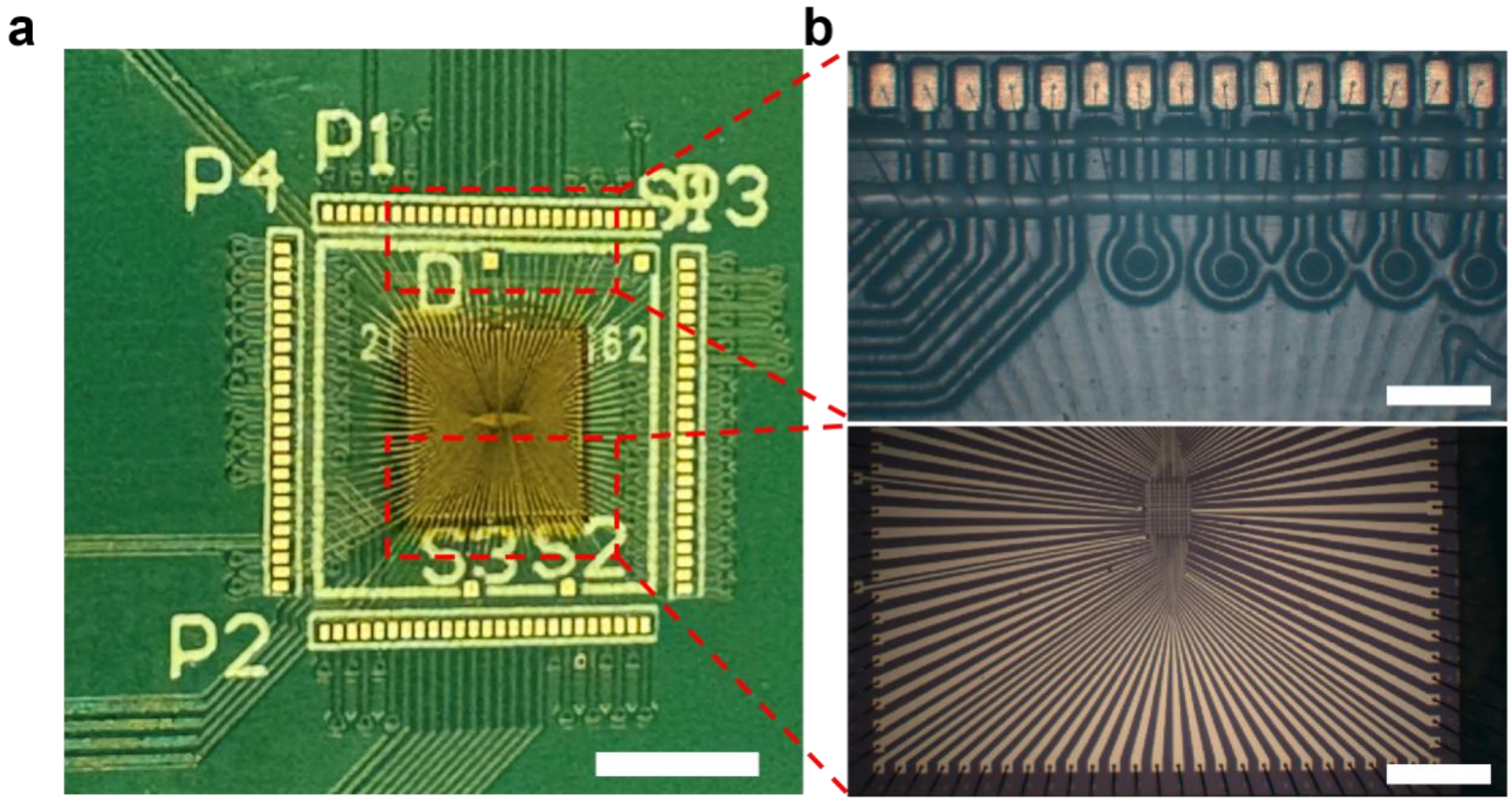

Supplementary Figure 27. Optical characterization of the ITO-based FGM-integrated Micro-LED array. a, Overview image of the Micro-LED array wire-bonded to a socket, showing the full layout. Scale bar, 5 mm. **b**. Zoomed-in of the dashed area indicated by the red box. Scale bar, 1 mm.

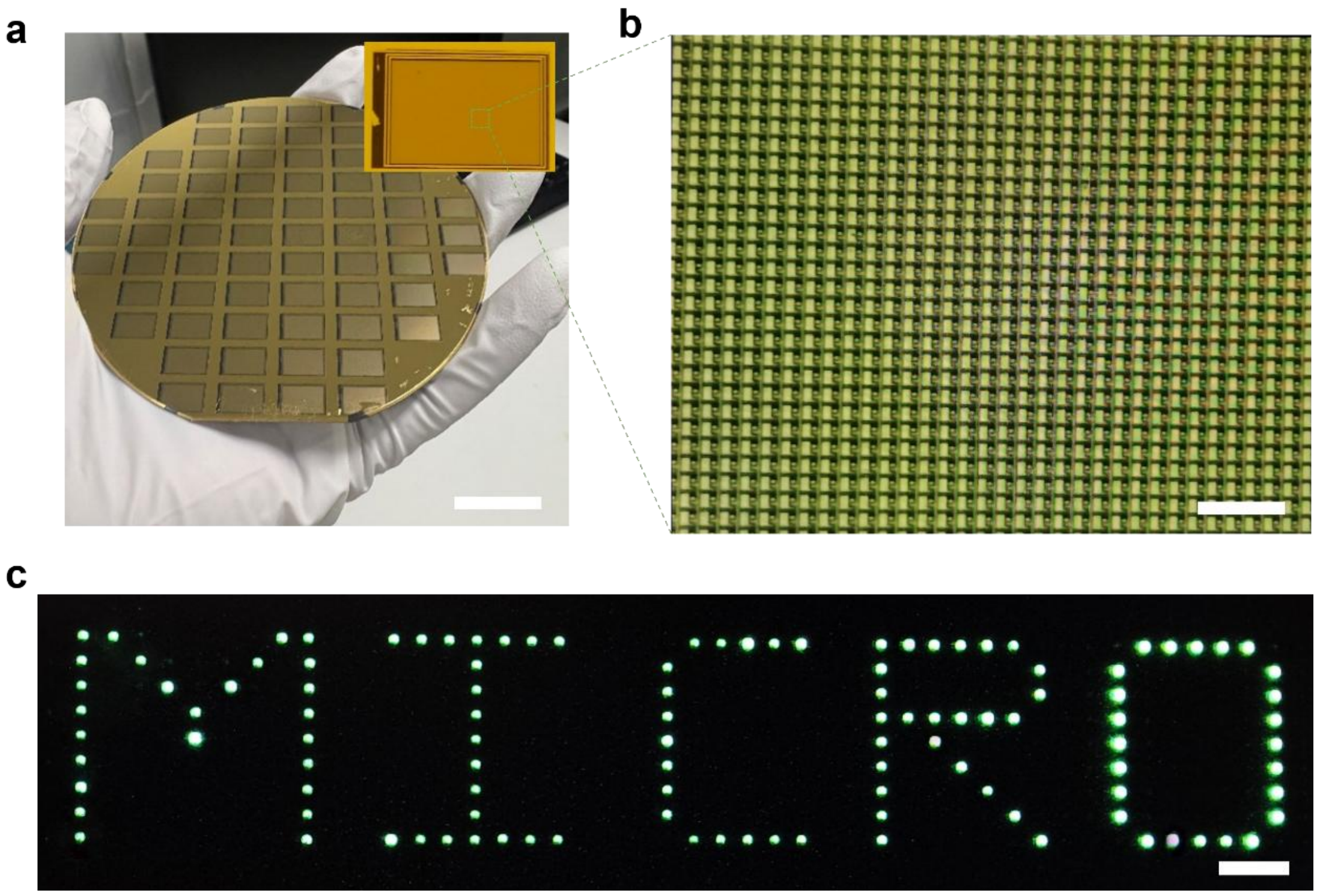

Supplementary Figure 28. Micro-LED display with large arrays of 200 × 128 pixels. a, Optical micrograph of the chips on a 4-inch wafer; the inset presents a single micro-display integrated with the ITO-based FGM devices. **b,** Magnified view of the integrated local arrays. c, Display of the text 'MICRO' in type-II modulation mode. Scale bars: 2 cm, 200 μm and 100 μm for a-c, respectively.

Supplementary Tabel S1 Summary of recent advances in various floating-gate memory devices.

Device structure	P/E Voltage (V)	Reading Voltage (V)	Reading current (A)	On/off ratio	Leakage current (pA)	Switching Energy (fJ)	Retention	Endurance (cycles)	Large-scale fabrication	Ref
MoS_2 / h-BN /Gra	+8 / -8	0.5	3.9×10^{-6}	1×10^{8}	/	14.4	>1000 s	/	no	6
MoS_2 / h-BN / Gra	+35 / -35	0.05	5×10^{-6}	$\approx 5 \times 10^{6}$	~4	0.0028	5300 s	1390	no	7
MoS_2 / h-BN / Te	+15 / -15	0.1	0.1 $\mu A\cdot\mu m^{-1}$	$\approx 10^{8}$	$>10^{-6}$ $\mu A\cdot\mu m^{-1}$	0.075	4000 s	4000	no	8
MoS_2 / h-BN / MLG	+25 / -25	0.05	$\approx 3 \times 10^{-6}$	1×10^{8}	> 100	0.0604	10^4 s	4200	no	9
MoS_2 / h-BN / Gra	+20 / -20	0.1	$\approx 5 \times 10^{-6}$	$\approx 10^{5}$	48.2	771.2	>1500 s	1000	no	10
BP / Al_2O_3 / BP	+10 / -10	-0.05	$\approx 3 \times 10^{-6}$	$\approx 10^{6}$	≈ 20	~ 2	>100 s	5000	no	11
DPPDTT / Al_2O_3 / Au NPs	+100 / -100	60	$\approx 10^{-3}$	$\approx 10^{3}$	$\approx 10^{5}$	10^{8}	/	/	Yes (solution)	12
InSe / h-BN / MLG	+30 / −30	-1	$\approx 10^{-5}$	$\approx 10^{8}$	> 100	> 0.06	3000 s	860	no	13
ZnO / Al_2O_3 / ZnO	+15 / -15	0.1	$\approx 10^{-6}$	$\approx 10^{7}$	~ 6	~9	10^4 s	10^4	Yes (ALD)	14
IGZO / Al_2O_3 / ZnO	+20 / -20	0.1	$\approx 10^{-6}$	$\approx 10^{5}$	6.62	3.972	1800 s	/	Yes (ALD)	15
ITO / Al_2O_3 / ITO	+15 /-12	0.05	4.3×10^{-5}	1.8×10^{8}	7.61	1.14	6000 s	>4000	Yes (sputter)	This work
ITO / Al_2O_3 / ITO	+8 /-8	0.05	2.43×10^{-5}	1.2×10^{8}	3.84	0.372	6000 s	>8000	Yes (sputter)	This work

Note: The superior memory performances can be primarily attributed to the high-quality interfaces and optimized device architecture. The ultra-smooth surface achieved by CMP ensures clean and uniform ITO/Al_2O_3/ITO interfaces with suppressed trap density, leading to efficient charge transport and improved retention. Meanwhile, the planar three-terminal configuration minimizes process-induced damage and preserves material integrity, resulting in enhanced device stability and uniformity.

Supplementary Tabel S2 Summary of the power consumption and stability in Micro-LED devices with various driver technology.

Display type	Driver type	Voltage (V)	Leakage Current (A)	On/off current ratio	Switch energy	Retention (s) Charge/Brightness	Endurance	Ref.
Micro-LED	In-memory transistors	-35	$>10^{-12}$	$<10^{7}$	17.5 pJ	NA / 60	1000	16
Micro-LED	Memristor	4.3	10^{-8}	$<10^{6}$	~ 10^{6} pJ	$>10^{4}$ / NA	> 480	17
Micro-LED	Memristor	0.17	10^{-9}	$<10^{7}$	2×10^{5} pJ	$>10^{4}$ / NA	10000	18
Micro-LED	Fe-MFFT	8	10^{-14}	4×10^{8}	Sub-pJ	NA / 50	10^{8}	19
Micro-LED	Fe-TFT	6	$>10^{-13}$	$<10^{8}$	Sub-pJ	NA / 100	10^{8}	20
Micro-OLED	Fe-OLET	60	$>10^{-10}$	$<10^{7}$	>300 pJ	10^{4}/ NA	>100	21
Micro-LED	MoS_2- TFT	6.2	6.41×10^{-13}	$<10^{8}$	1192 pJ (for 5 min)	NA	NA	1
Micro-LED	Poly-Si TFT	8	5×10^{-11}	$<10^{8}$	1.2×10^{5} pJ (for 5 min)	NA	NA	5
Micro-LED	ITO-based TFT	8	5.33×10^{-12}	~10^{7}	11560 pJ (for 5 min)	NA	NA	22
Micro-LED	ITO-FGM	15	7.61×10^{-12}	$>1.8\times10^{8}$	11.43 pJ	6000 / 200	>4000	This
Micro-LED	ITO-FGM	8	3.84×10^{-12}	$>1.2\times10^{8}$	3.07 pJ	6000 / 200	>8000	work

Supplementary Reference:

1. Hwangbo, S., Hu, L., Hoang, A. T., Choi, J. Y. & Ahn, J.-H. Wafer-scale monolithic integration of full-colour Micro-LED display using MoS_2 transistor. *Nat. Nanotechnol.* **17**, 500-506 (2022).
2. Yeo, Y. C. et al. Direct tunneling gate leakage current in transistors with ultrathin silicon nitride gate dielectric. *IEEE Electron Device Lett*. **21**, 540-542 (2000).
3. Deng, M.-Y. et al. Reducing power consumption of active-matrix mini-LED backlit LCDs by driving circuit. *IEEE Trans. Electron Devices* **68**, 2347-2354 (2021).
4. Meng, W. et al. Three-dimensional monolithic Micro-LED display driven by atomically thin transistor matrix. *Nat. Nanotechnol*. **16,** 1231-1236 (2021).
5. Hwang, K. et al. 1.6 - Inch Transparent Micro - Display with Pixel Circuit Integrated Micro-LED Chip Array by Misalignment - Free Transfer. *Adv. Mater*. **37**, 2416015 (2025).
6. Sun, X. *et al.* Reconfigurable logic-in-memory architectures based on a two-dimensional van der Waals heterostructure device. *Nat. Electron.* **5**, 752-760 (2022).
7. Liu, L. *et al.* Ultrafast non-volatile flash memory based on van der Waals heterostructures. *Nat. Nanotechnol.* **16**, 874-881 (2021).
8. Zha, J. *et al.* A 2D heterostructure - based multifunctional floating gate memory device for multimodal reservoir computing. *Adv. Mater.* **36**, 2308502 (2024).
9. Wang, H. *et al.* Ultrafast non - volatile floating - gate memory based on all - 2D materials. *Adv. Mater.* **36**, 2311652 (2024).
10. Li, W. *et al.* Demonstration of nonvolatile storage and synaptic functions in all-two-dimensional floating-gate transistors based on MoS_2 channels. *ACS Appl. Electron. Mater.* **5**, 4354-4362 (2023).
11. Xiong, X., Wang, X., Hu, Q., Li, X. & Wu, Y. Flexible synaptic floating gate devices with dual electrical modulation based on ambipolar black phosphorus. *Iscience* **25,** 103947, (2022).
12. Chen, Y. *et al.* Nanofloating gate modulated synaptic organic light-emitting transistors

for reconfigurable displays. *Sci. Adv.* **8**, eabq4824 (2022).

13. Wang, H. *et al.* Ultrafast‐Programmable 2D Homojunctions Based on van der Waals Heterostructures on a Silicon Substrate. *Adv. Mater.* **35**, 2301067 (2023).
14. Zhang, N. *et al.* Transparent floating gate memory based on ZnO thin film transistor with controllable memory window. *IEEE J. Electron Devices Soc.* **10**, 275-280 (2022).
15. Ryoo, H.-J. & Yoon, S.-M. Channel conductance modulation of dual-gate charge-trap oxide synapse TFT using In-Ga-Zn-O channel and ZnO trap layers. *IEEE Electron Device Lett.* **41**, 1661-1664 (2020)
16. Wang, F. et al. Micro-LED/van der Waals heterointegration for in-pixel processing display architecture. Nat. Commun. 17 (2026).
17. Yuk, S. H. et al. Transparent Active‐Matrix Micro‐LED Array Driven by Vertically Stacked TiO_2/HfO_2‐Based Memristors. Adv. Mater. Technol. 11, e01709 (20226).
18. Lee, H. J. et al. Monolithic integration of sub-volt memristor-driven pixels for capacitorless active-matrix Micro-LED displays. Int. J. Extrem. Manuf. 8, 025501 (2026).
19. Huang, T. et al. Flexible active-matrix Micro-LED display with 1T-1FeMFET architecture featuring scaling-limit-free design. Nat. Commun. (2026).
20. Jin, T. et al. Demonstration of programmable light intensity of a Micro-LED with a Hf-based ferroelectric ITZO TFT for Mura-free displays. Nanoscale Adv. 5, 1316-1322 (2023).
21. Xu, M. et al. Nonvolatile Memory Organic Light-Emitting Transistors. Adv. Mater. 35, 2307701-2307703 (2023).
22. Wang, Y. et al. Atomically Thin ITO Transistor Matrix for Monolithic Integration of Ultrabright Micro-LED Displays. Nano Lett. 25, 18091-18099 (2025).
