# Supplementary material for: Dual-function floating-gate memory driver for energy-efficient integrated display–illumination system
Source: Nat Commun. 2026 Aug 3;17:9313. doi: 10.1038/s41467-026-76263-3 (PMC13530162; doi:10.1038/s41467-026-76263-3)
Supplement: Supplementary file 2 [file 41467_2026_76263_MOESM2_ESM.pdf]

Description of Additional Supplementary Files:

Supplementary Video 1. The switching and retention process for Micro-LED integrated with ITO-based FGMs.
