# Supplementary material for: Dual-function floating-gate memory driver for energy-efficient integrated display–illumination system
Source: Nat Commun. 2026 Aug 3;17:9313. doi: 10.1038/s41467-026-76263-3 (PMC13530162; doi:10.1038/s41467-026-76263-3)
Supplement: Supplementary file 3 [file 41467_2026_76263_MOESM4_ESM.pdf]

nature portfolio

Peer Review File

Dual-function Floating-Gate Memory Driver for Energy-Efficient Integrated Display–Illumination System

Corresponding Author: Professor Anlian Pan

This file contains all reviewer reports in order by version, followed by all author rebuttals in order by version.

Version 0:

Reviewer comments:

Reviewer #1

(Remarks to the Author)
This manuscript demonstrated an Integrated Nonvolatile Display–Illumination System (INDIS) by monolithically integrating Micro-LED architecture based on transparent indium tin oxide (ITO) floating-gate memory (FGM) devices fabricated on 4-inch wafers. Notably, the FGMs offer dual-mode operation with an on/off current ratio exceeding 109, excellent retention stability beyond 2000 s, offering a scalable and energy-efficient solution for next-generation micro-display applications. Micro-light-emitting diodes (Micro-LEDs), after nearly a decade of development, have emerged as the most promising emissive technology. Significant efforts have been devoted toward the in-situ integration of Micro-LED arrays with driving units. There is an urgent need for a new circuit architecture that can support both persistent image retention and dynamic pattern switching.

The findings are novel and of broad interest to the community. This is an outstanding paper that promises to be highly influential. The impressive and robust results are supported by sound and logical descriptions throughout the majority of the manuscript.

However, there are some lacking in the technical explanations, which mislead to understand the research work. A revision is required to further stress it, there are some suggestions for the authors to consider:

1. Supplementary Figure 2 provides fabrication process and thicknesses of some semiconductor/ dielectric layers, however, the dimensions of metal electrodes were not mentioned. Adding these detailed dimensions will aid in providing readers with a clearer understanding of the device and assist in the reproduction of the relevant results. In Fabrication of Micro-LED arrays, the blue and green Micro-LEDs have been fabricated, the content of In in MQWs should be provided separately.

2. According to the Supplementary Tabel 1, the performance of ITO/Al2O3/ITO floating-gate memory devices exceeds most of the counterparts employing the other materials. The possible mechanism for this advancement could be discussed.

3. Line 136, page 5, ‘By enabling collective programming and erasing of pixels through simplified biasing, this mode facilitates system-level light modulation with reduced circuit complexity’. In display the pixels should be refreshed in required frequency, such as exceeds 60 Hz at least, how about the response time (rise and fall) of the proposed architecture.

4. Line 157, page 6, ‘This dual-mode programmability supports both static image holding and dynamic light modulation, offering reconfigurable functionality essential for next-generation display-illumination platforms.’ One of the possible typical display-illumination applications could be provided to enable a clearer understanding of the application prospects.

5. Line 189, page 7, ‘the standalone Micro-LED (217 μA, corresponding to a current density of 385.7A·cm-2).’ Have the EQE curves of standalone Micro-LEDs been measured? Perhaps the current density of 385.7 A·cm-2 is too high for the blue and green Micro-LEDs, which may face severe heat generation originating from Auger non-radiative recombination.

6. Line 200, page 7, ‘The current also exhibits excellent long-term retention, with the Micro-LED remaining steadily illuminated for over 200 seconds with negligible decay in brightness.’ However, in Figure 3d, it looks like that the current density for 15 V declines almost 15 A·cm-2 over 200 seconds. This result could be clarified. Furthermore, the long-term

retention in brightness is exhibited by photography, however, the values of luminance or photo counts collected by any spectrometer will be more convincing, for example those in Supplementary Figure 12.

7. Line 298, page 10, 'Then 110-nm thickness IO layer.' Please confirm the IO layer.

8. The pixel pitch is also very important for display applications since it determine the resolution. The manuscript demonstrated array of 564 PPI. This manuscript demonstrates an array achieving 564 PPI. A discussion on the implications of pixel pitch for floating-gate memory (FGM) integrated Micro-LEDs would be a valuable prospect.

9. The region between the neighboring Micro-LEDs is filled by the deposited of 2.1-μm $SiO2$ layer. Since the $SiO2$ layer is transparent. The optical crosstalk maybe a possible issue, which is visible in some subfigures in Figure 4. The impacts of planarization layer on optical crosstalk could be discussed.

Reviewer #2

(Remarks to the Author)
In this manuscript, the authors described multimodal floating-gate memory driver for energy-efficient integrated display-illumination system. However, the authors should respond to the following comment prior to publication in Nature Communications. The comments are as follows:

1. The gate programming voltage of ±15 V might be very high for large-scale integration and low-power systems. The authors should suggest a feasible method to reduce the operating voltage.
2. The authors should describe the durability performance of the switching characteristics in a TYPE-I mode.
3. The authors should describe the distribution of programming/erase threshold voltages during multiple switching cycles to explain the reliability of the device.
4. The authors should describe the effect of the current level in the high-resistivity state for the switching characteristic test on the system noise.
5. The authors should describe whether it is necessary to reset the device's conductivity state during the transition between multiple operating modes.
6. The authors should describe the possibility of the interference between Type-I and Type-II modes.
7. The authors should describe how the Micro-LEDs experience heat buildups under high-brightness operating conditions.
8. The authors should describe at least a table about comparison of the low power consumption and stability characteristics of the device studied in this work with those of the devices published in other literatures.

Reviewer #3

(Remarks to the Author)
This manuscript reports a micro-LED platform that integrates display and lighting functions using nonvolatile operation. The demonstration of monolithic integration at the 4-inch wafer scale with high yield is a notable strength and indicates a mature fabrication process with good scalability. Nevertheless, several key aspects of the device operation and the claimed conceptual novelty are not sufficiently clarified or supported by quantitative evidence. Addressing these issues would substantially improve the technical rigor and overall impact of the work. The following major and minor comments are provided to help strengthen the manuscript.

Major comments

1. Type I operation mechanism and charge injection process

In the Type I operation mode, program/erase is achieved via drain–source biasing, but the physical role of the tunneling dielectric (Al_2O_3) is not clearly explained. It is unclear whether the gate electrode is electrically floating and how a sufficient electric field is established across the tunneling dielectric using drain bias alone to induce hole injection into the charge trapping layer.
Furthermore, although the charge injection mechanism is described as Fowler–Nordheim tunneling, the reported Al_2O_3 thickness (~18 nm) is relatively large compared to conventional FN-based floating-gate memories. Clarification is needed as to whether FN tunneling is indeed dominant or whether other mechanisms, such as trap-assisted tunneling, may contribute. A clearer description of the charge injection pathways, electric field distribution, and the distinct roles of the gate and dielectric in Type I and Type II modes is required. Energy band diagrams and quantitative discussion of the electric field conditions would be highly beneficial.

2. Evaluation of nonvolatile operation

Although nonvolatile operation is a central claim of this work, its evaluation remains limited. The basis for selecting a retention time of 2000s is not defined, for example in terms of an acceptable percentage loss in optical output. It is also unclear whether longer-term retention measurements are feasible. Moreover, retention behavior is evaluated primarily using optical microscopy images, which provide only qualitative information. Quantitative metrics, such as the percentage change in optical output or luminance over time, should be provided to more convincingly substantiate the nonvolatile operation

claim.

3. Statistical support for device uniformity

The authors claim excellent uniformity; however, device-to-device and chip-to-chip statistical analyses are limited. In Figure 2g, the on/off ratio varies by up to a factor of ~4, yet the acceptability of this variation for display operation is not discussed. Providing I–V characteristics for all measured devices and, if available, chip-to-chip or position-dependent data across the 4-inch wafer would better support the scalability and uniformity claims.

4. Comparison with existing FGM-based devices

The energy and performance comparisons focus primarily on TFT-based drivers, which limits evaluation of the proposed FGM architecture itself. Even in the supplementary comparison among FGM-based devices, the presented metrics do not clearly demonstrate a distinct performance advantage of the proposed device. As improved retention enabled by the FGM is a central claim of this work, the lack of clear superiority over previously reported FGM-based drivers weakens the case for novelty.
A more critical and quantitative comparison with existing FGM-based devices, based on key metrics such as switching energy, retention, leakage current, and program/erase conditions, is necessary to convincingly establish the relative merits and technological significance of the proposed approach.

5. Use of the term “multi-modal”

The term “multi-modal” is typically used to describe systems that operate across distinct modalities or sensory domains. In the present manuscript, the term is used to denote the coexistence of lighting and display functions, which both belong to the optical domain. As such, the use of “multi-modal” may be conceptually inaccurate. The authors should reconsider this terminology or clearly justify its usage in the context of the proposed device.

Minor comments

1. The operating principle illustrated “on the right side of Figure 1a” (lines 97–98) requires clearer explanation.
2. The pulse width used for retention measurements in Figure 2e should be specified.
3. Figure 3g should include a legend.
4. The descriptions of Fig. 4b and Fig. 4c (line 262) appear to be interchanged.
5. The term “IO layer” in the Materials and Methods section (line 298) should be corrected or defined.
6. The origin and role of the ITO layer observed in Supplementary Figure 1a TEM image should be clarified.

Version 1:

Reviewer comments:

Reviewer #1

(Remarks to the Author)
The authors have made substantial and satisfactory revisions to address the concerns raised during the review process. The manuscript now meets the high standards of Nature Communications in terms of technical quality and clarity of presentation. Therefore, I recommend that the revised manuscript be accepted for publication in its current form.

Reviewer #2

(Remarks to the Author)
This manuscript has been improved according to the reviewers’ comments. However, minor revision is required for publication. The main comments are as follows:

1. A whole experimental data should be clearly described in Figure R12b.

Reviewer #3

(Remarks to the Author)
I appreciate the authors' efforts in revising the manuscript and providing additional explanations, figures, and supporting data. The added energy-band diagrams, device-uniformity data, and revised terminology significantly improve the clarity of the manuscript. However, there are still several critical points regarding the physical mechanism and fair metric comparisons that must be addressed before publication.

[Major Comments]

1. While the revised explanations and energy-band diagrams help clarify the intended Type-I and Type-II operations, the Type-I programming mechanism under a drain-only bias with a floating gate remains non-intuitive, particularly given the

relatively thick 18 nm Al2O3 tunneling layer. The authors cite previous two-terminal memory demonstrations (Nat. Commun. 2016, 7, 12725; Adv. Mater. 2017, 29, 1703363) which utilized highly conductive graphene floating electrodes to effectively establish an electric field across the tunneling dielectric. In the present work, however, both the top ITO channel and the bottom floating ITO layer appear to be fabricated via the same process, implying similar conductivity. Because the off-state current of the ITO channel is extremely low, the channel is nearly insulating in this state. Consequently, it is not immediately clear how the drain bias generates a sufficiently strong vertical electric field across the 18 nm Al2O3 layer, rather than predominantly dropping laterally along the top ITO channel. Please

a) Please explicitly confirm whether the underlying body/substrate/LED stack is electrically grounded or floating during the Type-I measurement, as this strongly influences the field distribution.

b) Please provide robust evidence demonstrating that a sufficient vertical field is established across the Al2O3 layer. This can be achieved by providing: An electrostatic simulation under actual Type-I bias conditions using realistic material parameters such as the specific conductivity of the ITO layers; OR Thickness-dependent tunneling-current measurements for the Al2O3 oxide similar to the cited Nat. Commun. work; OR Another clear experimental or theoretical demonstration.

2. The retention metric presented in Table S2 requires revision to ensure a fair comparison with the existing literature. The retention values cited for the reference Micro-LED devices correspond to the optical/light-output retention of integrated pixels. In contrast, the 6000 s value reported for this work appears to represent standalone FGM electrical retention. These two metrics are not directly comparable.

a) Update Table S2: Report the optical/luminance retention of the integrated 1M1D Micro-LED pixel for a fair comparison. (For example, the 200 s brightness-retention result from Figure S20, or the newly added 3600 s result from Figure R22)

b) Clarify Datasets: Explain the relationship between the 200 s brightness-retention data (Figure S20) and the 3600 s data (Figure R22). Although measured over vastly different time durations, the reported brightness losses are remarkably similar, ~9.5% and ~9.4%, respectively. Please clarify if these datasets were measured from different devices, and clearly define the representative luminance-retention value to be used consistently throughout the manuscript.

[Minor Comments]

1. Correct the y-axis label in Supplementary Figures S15e and S15f from "conts" to "counts."

2. Supplementary Figure S16 is currently difficult to interpret. While the purpose appears to be demonstrating improved current uniformity across the wafer, the current color scale and order obscure this improvement. Please revise the plot using a clearer, more intuitive, and consistent color scale to effectively support the claim of uniform device performance.

Version 2:

Reviewer comments:

Reviewer #3

(Remarks to the Author)
The authors have adequately addressed all the concerns raised during the review process. I believe the manuscript is now suitable for publication in its current form.

Open Access This Peer Review File is licensed under a Creative Commons Attribution 4.0 International License, which permits use, sharing, adaptation, distribution and reproduction in any medium or format, as long as you give appropriate credit to the original author(s) and the source, provide a link to the Creative Commons license, and indicate if changes were made.
In cases where reviewers are anonymous, credit should be given to 'Anonymous Referee' and the source.
The images or other third party material in this Peer Review File are included in the article's Creative Commons license, unless indicated otherwise in a credit line to the material. If material is not included in the article's Creative Commons license and your intended use is not permitted by statutory regulation or exceeds the permitted use, you will need to obtain permission directly from the copyright holder.
To view a copy of this license, visit https://creativecommons.org/licenses/by/4.0/

REVIEWER COMMENTS

Reviewer #1 (Remarks to the Author):

This manuscript demonstrated an Integrated Nonvolatile Display–Illumination System (INDIS) by monolithically integrating Micro-LED architecture based on transparent indium tin oxide (ITO) floating-gate memory (FGM) devices fabricated on 4-inch wafers. Notably, the FGMs offer dual-mode operation with an on/off current ratio exceeding 10^9, excellent retention stability beyond 2000 s, offering a scalable and energy-efficient solution for next-generation micro-display applications. Micro-light-emitting diodes (Micro-LEDs), after nearly a decade of development, have emerged as the most promising emissive technology. Significant efforts have been devoted toward the in-situ integration of Micro-LED arrays with driving units. There is an urgent need for a new circuit architecture that can support both persistent image retention and dynamic pattern switching.

The findings are novel and of broad interest to the community. This is an outstanding paper that promises to be highly influential. The impressive and robust results are supported by sound and logical descriptions throughout the majority of the manuscript.

However, there are some lacking in the technical explanations, which mislead to understand the research work. A revision is required to further stress it, there are some suggestions for the authors to consider:

Response: Thank you for your examination of our work and for your high praise. Regarding the issues you raised, we have carefully reviewed each one and provided detailed explanations and necessary supplements below.

1. Supplementary Figure 2 provides fabrication process and thicknesses of some semiconductor/ dielectric layers; however, the dimensions of metal electrodes were not mentioned. Adding these detailed dimensions will aid in providing readers with a clearer understanding of the device and assist in the reproduction of the relevant results. In Fabrication of Micro-LED arrays, the blue and green Micro-LEDs have been fabricated, the content of In in MQWs should be provided separately.

Response: We sincerely thank the reviewer for the valuable comments regarding the detailed information in fabrication process, which is helpful for improving the clarity and reproducibility of our manuscript.

1). Metal electrodes dimensions: In our device, the drain, source, and gate electrodes were defined by standard photolithography followed by electron beam evaporation (EBE) of Cr/Au

(5 nm/50 nm). The lateral dimensions of the electrodes (including channel length, channel width, and contact pad size) have now been explicitly added in the revised manuscript. These details are incorporated both in the fabrication section and in the caption of Supplementary Figure 2 for clarity.

2). Indium content in MQWs: As you comment, the In composition in the InGaN/GaN multiple quantum wells (MQWs) plays a key role in determining the emission properties. In our work, the indium composition is ~15% for blue Micro-LEDs and ~25% for green Micro-LEDs. In the revised manuscript, we have explicitly provided these values separately for the blue and green devices to ensure better transparency and reproducibility.

In the revised Supplementary information, such information is added in the corresponding part.
"Metal electrodes dimensions" are added in Page 5, line 68 and line 79 in the Supplementary information.
"Indium content in MQWs" is added in Page 5, line 67 in the Supplementary information.

2. According to the Supplementary Tabel 1, the performance of ITO/Al_2O_3/ITO floating-gate memory devices exceeds most of the counterparts employing the other materials. The possible mechanism for this advancement could be discussed.

Response: Thank you for your insightful comment and for highlighting the performance of the ITO/Al_2O_3/ITO floating-gate memory devices presented in Supplementary Table 1. In the revised manuscript, we have supplemented the discussion, and the possible origins of the enhanced performance can be attributed to the following aspects:

1). **High-Quality Interface and Material Compatibility:** A key factor is the excellent interface quality enabled by our fabrication process. Specifically, after mesa formation, a SiO_2 layer was deposited followed by chemical mechanical polishing (CMP), resulting in an ultra-flat surface. As shown in Figure R1, the RMS roughness is as low as 0.585 nm, which provides an ideal platform for subsequent layer stacking. This ultra-smooth surface ensures that the interfaces within the ITO/Al_2O_3/ITO stack are clean and uniform, effectively suppressing interfacial trap density. As a result, charge scattering and unintended trapping are minimized, leading to more efficient charge injection, retention, and improved device stability.

In addition, using indium tin oxide (ITO) as both the channel material and the floating gate ensures excellent material compatibility and energy band alignment, which facilitates carrier transport and reduces interface-related degradation mechanisms.

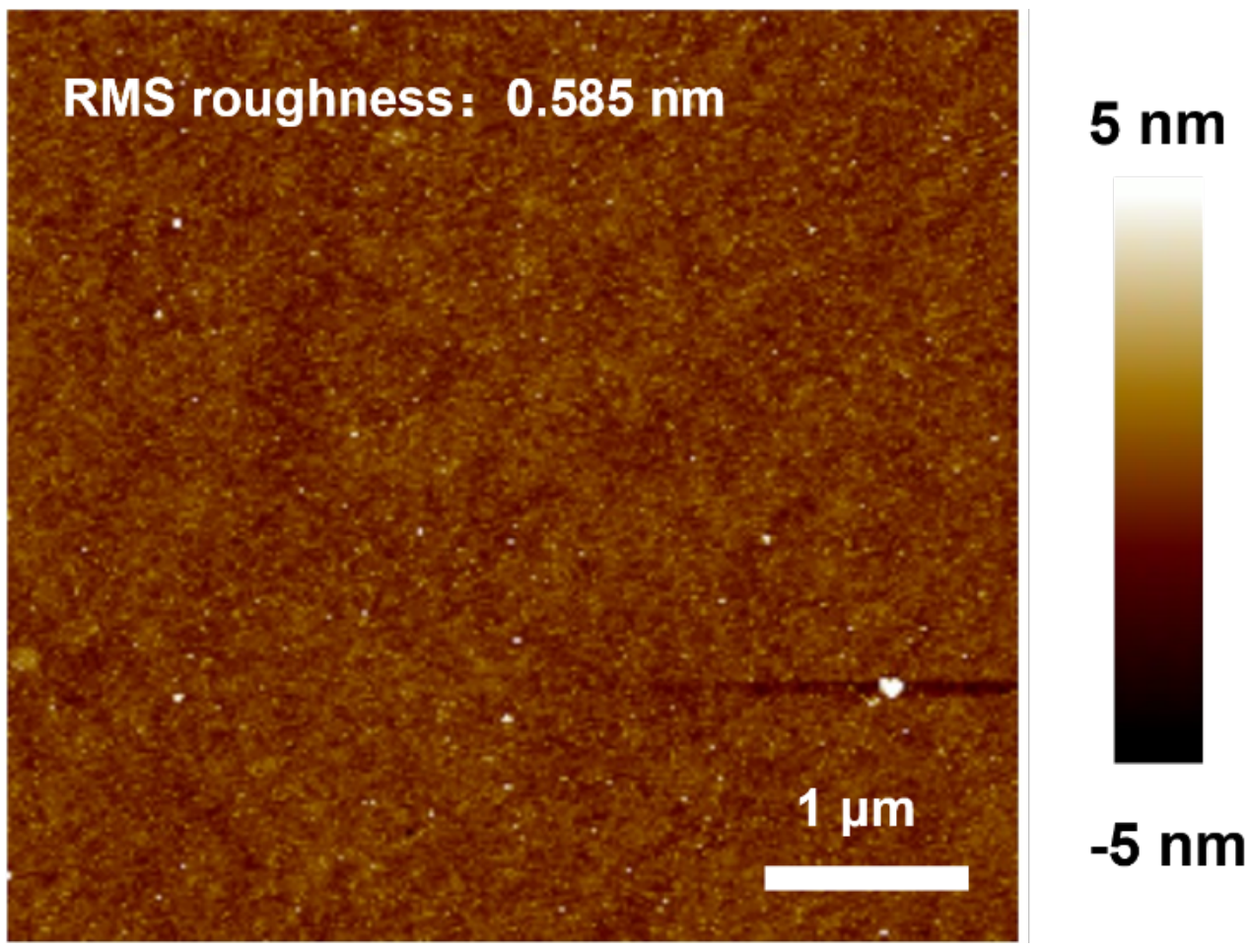

Figure R1: Atomic Force Microscope (AFM) image of mesa top after CMP process. The RMS roughness is 0.585 nm.

2). **Advanced device architecture design**: The superior performance is also closely related to the device architecture. As illustrated in Fig. R2 (Fig. 2a in the revised manuscript), the device adopts a planar three-terminal configuration, where the source, drain, and control gate are co-fabricated within the same plane. This design allows all electrodes to be defined in a single evaporation step, thereby avoiding repeated lithography, solvent exposure, and high-temperature processing that could otherwise damage the semiconductor channel or degrade interfaces. Such a fabrication strategy not only preserves the intrinsic material quality but also improves device uniformity and scalability, contributing to enhanced electrical performance compared with previously reported counterparts.

These combined advantages in interface engineering and structural design are believed to be the primary reasons for the improved performance observed in Supplementary Table S1.

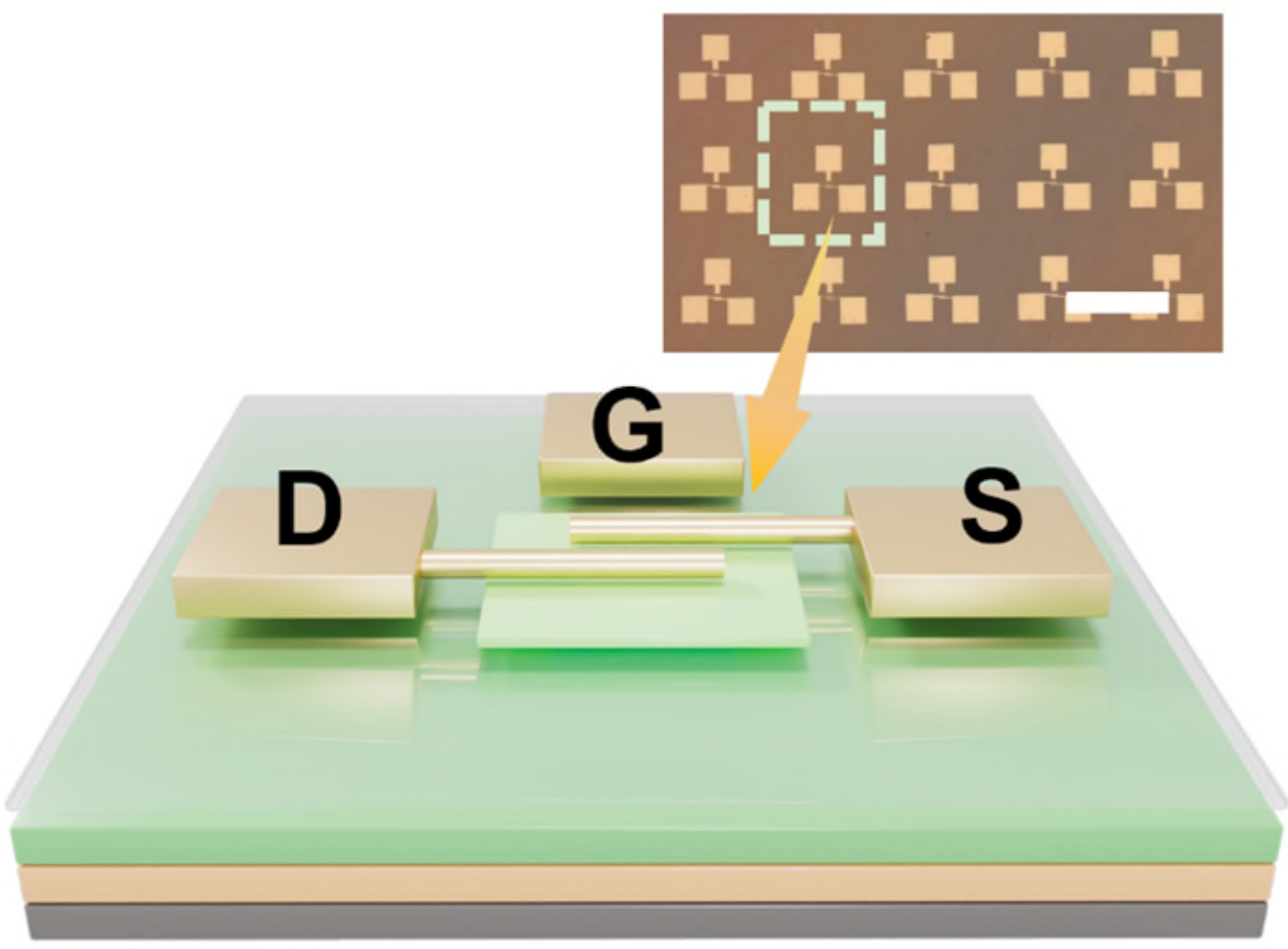

Figure R2 (Revised Figure 2a): Schematic diagram of the FGM device structure. The inset shows an optical image of a device array fabricated on GaN-based epitaxial wafers after mesa

formation. Scale bar, 300 μm.

In the revised manuscript, Figure 2a has been updated to more clearly illustrate the planar three-terminal device structure.

The origins of the enhanced performance are added after Supplementary Table S1 (page 32) and as indicated below.

"The enhanced performance can be primarily attributed to the high-quality interfaces and optimized device architecture. The ultra-smooth surface achieved by CMP ensures clean and uniform ITO/Al_2O_3/ITO interfaces with suppressed trap density, leading to efficient charge transport and improved retention. Meanwhile, the planar three-terminal configuration minimizes process-induced damage and preserves material integrity, resulting in enhanced device stability and uniformity."

3. Line 136, page 5, 'By enabling collective programming and erasing of pixels through simplified biasing, this mode facilitates system-level light modulation with reduced circuit complexity'. In display the pixels should be refreshed in required frequency, such as exceeds 60 Hz at least, how about the response time (rise and fall) of the proposed architecture.

Response: We sincerely thank the reviewer for this professional and insightful comment regarding the dynamic response and refresh capability of the proposed architecture, which is indeed critical for display applications.

In response, we have further clarified the dynamic performance of our device in the revised manuscript and Supplementary Information. Specifically, we experimentally evaluated the transient response of the integrated 1M1D device under gate voltage pulses. As shown in Fig. R3, voltage pulses of 50 μs (-12 V/+15 V) are alternatively applied on the device. As a result, voltage rise time and fall time of ~11 μs and ~10 μs are obtained, respectively, indicating fast-switching capability. Based on this response speed, the pixel refresh process—consisting of programming and erasing (i.e., two gate scans for on/off states)—requires a total time of ~200 μs per frame. This corresponds to an effective refresh rate of approximately 5 kHz, which is significantly higher than the typical requirement for display applications (e.g., >60 Hz).

These results demonstrate that the proposed architecture not only supports simplified biasing for system-level light modulation but also fully satisfies, and substantially exceeds, the refresh rate requirements for practical display operation. We have also incorporated this discussion into the revised manuscript to better highlight the high-speed operation capability of the system.

In the revised manuscript, the refresh rate is added in page 6 line 173, and the detailed response time (rise and fall) and refresh rate are added in Supplementary Figure 14 (page 17, line 210).

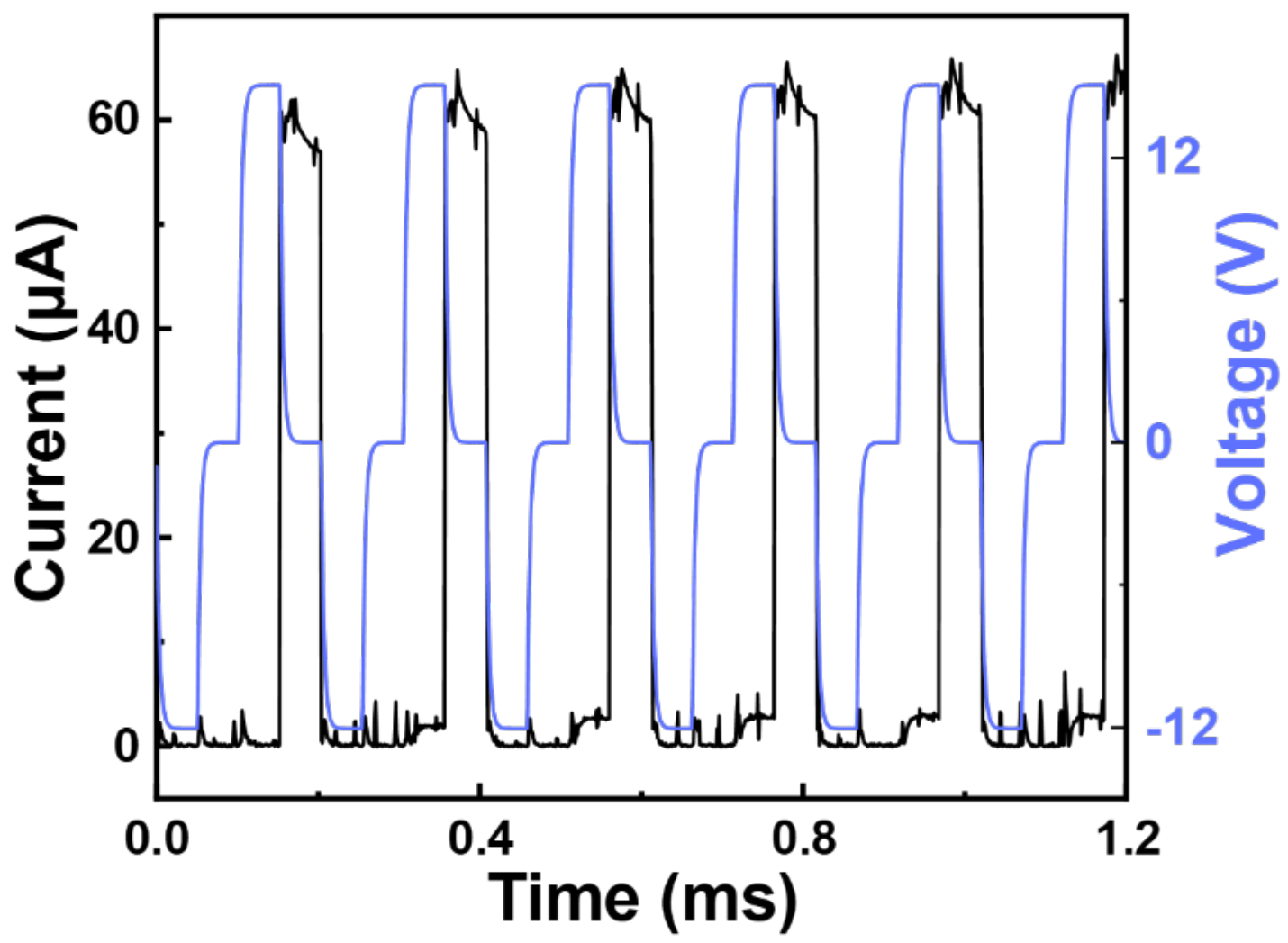

Figure R3: Demonstration of high-frequency operation in type-II mode by sequential programming (+15 V) and erasing (-12 V) pulses with ~50 μs intervals.

4. Line 157, page 6, 'This dual-mode programmability supports both static image holding and dynamic light modulation, offering reconfigurable functionality essential for next-generation display-illumination platforms.' One of the possible typical display-illumination applications could be provided to enable a clearer understanding of the application prospects.

Response: We sincerely thank the reviewer for this valuable suggestion. In the revised manuscript, we have added a typical application example based on a digital headlight system with pixelated illumination arrays, where the Integrated Nonvolatile Display–Illumination System (INDIS) can simultaneously realize lighting and information display. Specifically, under the **drain modulation mode**, a programming pulse is applied to the anode to store holes in the charge-trap layer, thereby reducing the channel resistance. Upon applying a read voltage, all Micro-LED pixels are uniformly turned on, generating high-brightness illumination suitable for nighttime driving. In contrast, under the **gate modulation mode**, selective programming of individual floating-gate memory units enables pixel-level control. Only the programmed pixels emit light under a common read voltage, allowing the system to display customizable patterns, symbols, or text.

Based on these two operation modes, the INDIS system can flexibly switch between **full-field illumination and high-resolution patterned display** in complex driving environments. For example, it can project warning signs, pedestrian crossing indicators, or simple dynamic symbols onto the road while maintaining sufficient illumination, thereby enhancing real-time information interaction between vehicles and their surroundings.

Furthermore, when integrated with onboard navigation systems and projection optics, the system can project directional guidance (e.g., navigation arrows) directly onto the road surface, improving driving safety and intuitiveness. In addition, pixel-level programmability enables adaptive lighting strategies under different traffic scenarios (e.g., urban streets, highways), optimizing both visibility and energy efficiency.

We believe this example clearly demonstrates the unique advantage of dual-mode programmability and its potential for next-generation display–illumination platforms.

In order to facilitate readers' comprehension of the INDIS developed in our work, we add this potential application into the principles section, corresponding to Supplementary Note 1 (page 6 line 89), with the specific description as follows:

The INDIS system flexibly switches between full-field illumination and high-resolution patterned display in complex driving environments. For instance, it can project warning signs, pedestrian indicators, or navigation arrows onto the road while maintaining adequate ambient lighting, enabling real-time vehicle-to-surrounding information exchange. Additionally, its pixel-level programmability supports adaptive lighting strategies for different traffic scenarios (e.g., urban streets vs. highways), balancing visibility and energy efficiency.

5. Line 189, page 7, 'the standalone Micro-LED (217 μA, corresponding to a current density of 385.7A·cm^{-2}).' Have the EQE curves of standalone Micro-LEDs been measured? Perhaps the current density of 385.7 A·cm^{-2} is too high for the blue and green Micro-LEDs, which may face severe heat generation originating from Auger non-radiative recombination.

Response: Thanks for your professional comment. According your comment, the EQE of the standalone Micro-LEDs has been measured and is now included in the Fig. R4. At a current density of 385.7 A·cm^{-2}, the EQE values are 7.52% (blue) and 5.16% (green), showing moderate reductions compared to their peak values (10.78% and 7.94%, respectively), indicating that efficiency droop remains within an acceptable range. The values are also superior to previously reported results (IEEE Transactions on Electron Devices, 2023, 70(9): 4689-4693, Photonics Research, 2023, 11(4): 549-557).

We also agree that such a high current density may induce heat generation and Auger non-radiative recombination. In our devices, this effect is mitigated by improved thermal dissipation, mainly due to the implementation of metallic interconnects on the bottom side, which help reduce heat accumulation.

In addition, the reported current density here corresponds only to the maximum driving condition, demonstrating the capability of the device to operate at high current and high brightness. In practical applications, the device can be operated at lower current densities as

needed, thereby reducing thermal effects and achieving higher efficiency.

In the revised manuscript (page 7, line 198) and Supplementary Note 3 (page22, 266), we have added the EQE characteristics and the related thermal effect analysis and discussion.

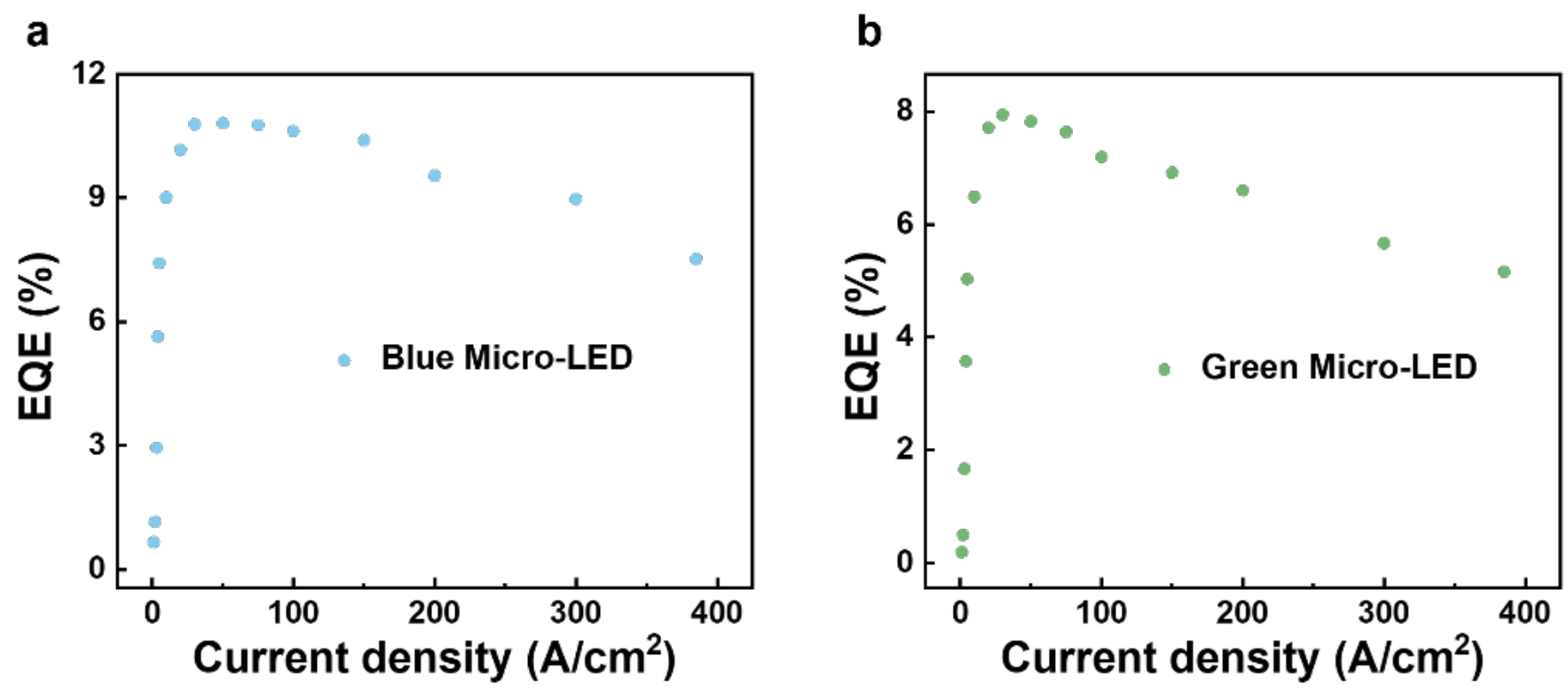

Figure R4: External quantum efficiency (EQE) characteristics of the micro-LED. Blue (a) and green (b), respectively.

6. Line 200, page 7, 'The current also exhibits excellent long-term retention, with the Micro-LED remaining steadily illuminated for over 200 seconds with negligible decay in brightness. However, in Figure 3d, it looks like that the current density for 15 V declines almost 15 A·cm^{-2} over 200 seconds. This result could be clarified. Furthermore, the long-term retention in brightness is exhibited by photography, however, the values of luminance or photo counts collected by any spectrometer will be more convincing, for example those in Supplementary Figure 12.

Response: Thanks for your insightful comments. First, regarding Fig. 3d, we acknowledge that a gradual decrease in current density is observed under a 15 V gate pulse. Quantitatively, the current decreases from 64.9 μA to 58.3 μA over 200 s, corresponding to a reduction of ~10.62 A·cm^{-2}. Despite this decrease, the device retains ~90.61% of its initial current under relatively high current density operation (~100 A·cm^{-2}), indicating reasonably stable retention behavior. We have clarified this point in the revised manuscript for better accuracy and transparency.

Second, we fully agree that quantitative optical characterization would provide more convincing evidence than photographic images alone. In response, we have added the luminance evolution during retention in the revised manuscript (Fig. R5b and R5c). The results show that, under different gate pulse voltages (9 V, 10 V, 12 V and 15V; pulse width of 1 s), the brightness retention remains at 96.87%, 92.27%, 90.62%, and 90.51%, respectively, after 200 s. These results are consistent with the electrical retention behavior and further confirm the stable light output of the device.

To strengthen the reliability of the retention analysis, these additional clarifications and quantitative results have been incorporated into the revised manuscript (page 8, line 214), Supplementary Note 3 (page 22, line 266) and Supplementary Figure 20 (page 21, line 247).

Moreover, the brightness was quantitatively measured over time under a 10 V gate pulse with a read voltage of 2.8 V. As shown in the Figure R6, the initial brightness reached 1.34 × 10^6 Cd/m^2, and the decreases of brightness were only 0%, 0.48%, and 7.72% after 50 s, 100 s, and 200 s of continuous operation, respectively, showcasing outstanding retention performance. We believe that the device can meet the stability requirements of most reconfigurable display applications. In the revised Supplementary information, Supplementary Figure 21 (page 21, line 256) has been updated to more convincingly display the variation of the illumination during retention process.

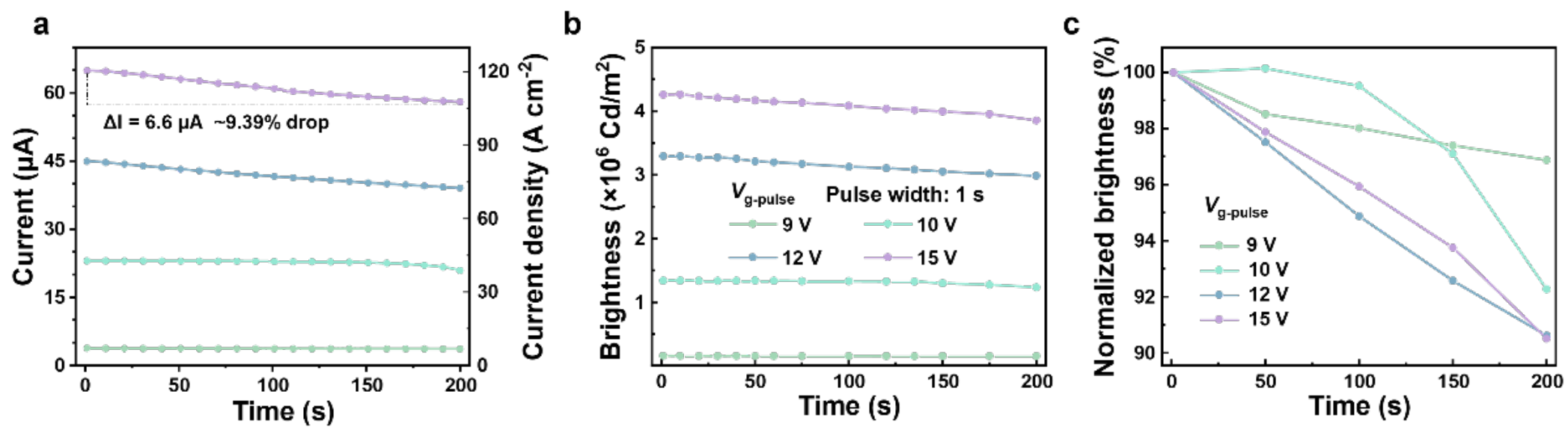

Figure R5: The retention characteristics of the 1M1D under different gate voltage. Current (a) and brightness (b) and (c), respectively.

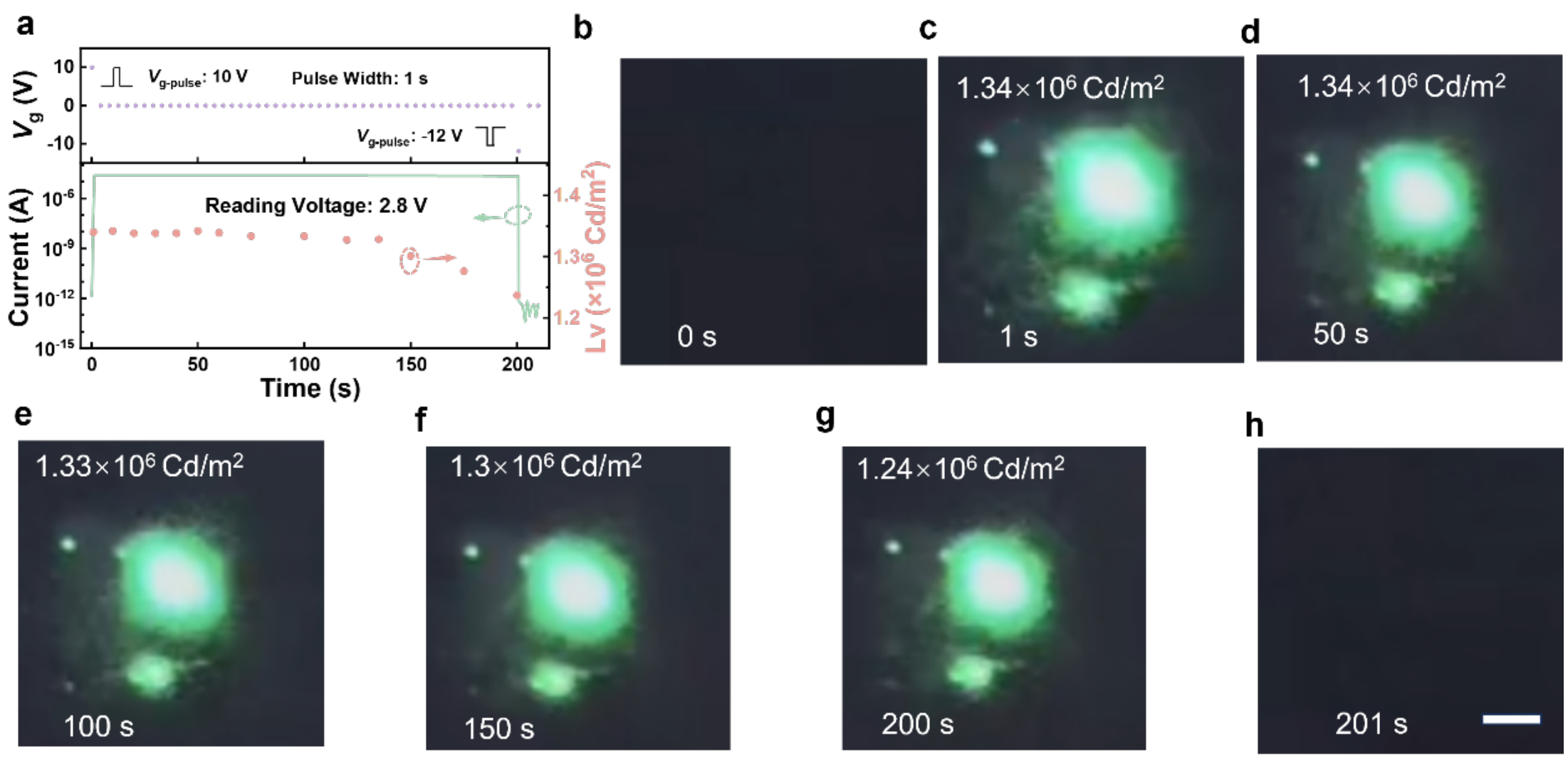

Figure R6 (Supplementary Figure 10): Retention characteristics of the 1M1D pixel acquired from Supplementary video 1. a, Time-dependent variation of current after a pulse voltage (10 V, 1 s) applied onto the gate, with the reading voltage of 2.8 V. The results indicate negligible variation in driving current and brightness after 200 s. b, Initial optical image of Micro-LED

before gate voltage pulse. c, The image of Micro-LED after a positive pulse voltage applied onto the gate. d-g, The images of Micro-LED after a certain duration following the removal of gate voltage, corresponding to 50 s (d), 90 s (e), 150 s (f) and 200 s (g). f, The image of Micro-LED after a negative pulse voltage applied onto the gate. Scale bar, 10 μm. The results indicate excellent brightness retention.

7. Line 298, page 10, 'Then 110-nm thickness IO layer.' Please confirm the IO layer.

Response: We thank the reviewer for pointing out the typo in Line 298, Page 10 regarding "IO layer." Upon rechecking the manuscript, we confirm that "IO" in this context refers to indium tin oxide (ITO), which is used to achieve an ohmic contact with p-GaN.

To avoid a misunderstanding and ensure clarity for readers, we have made the following revision:

Original text: "Then 110-nm thickness IO layer."
Revised text: "Then 110-nm thickness ITO layer."

8. The pixel pitch is also very important for display applications since it determine the resolution. The manuscript demonstrated array of 564 PPI. This manuscript demonstrates an array achieving 564 PPI. A discussion on the implications of pixel pitch for floating-gate memory (FGM) integrated Micro-LEDs would be a valuable prospect.

Response: We thank the reviewer for this insightful comment. The pixel pitch is indeed a key parameter for display applications, as it directly determines the achievable resolution. The demonstrated Micro-LED array with 564 PPI already represents a high-resolution level for in-plane driving integrated display pixels, comparable to or exceeding current state-of-the-art arrays reported in the literature. This demonstrates the potential of our approach for high-density, high-performance displays.

For future work, the resolution can be further improved through two main strategies.

First, reducing the lateral dimensions of the floating-gate memory channels would allow pixels to be placed closer together, decreasing the pixel pitch and increasing the PPI. In this work, relatively large channels (L/W = 16/1 μm) were used to achieve higher on-state current. Future designs with smaller channels (e.g., L/W = 5/1 μm or smaller) would directly contribute to higher pixel density.

Second, vertically stacked integration using transparent functional layers (ITO/Al_2O_3/ITO) could enable a 3D configuration of Micro-LEDs with FGM circuits, further reducing the

effective pixel pitch and maximizing display resolution.

In the revised manuscript, we add this discussion (page 9, line 270) as follows:

The fabricated Micro-LED array comprises 10 × 10 pixels with a pitch of 45 μm, corresponding to a pixel density of 564 PPI. Each pixel consists of a 7.5-μm Micro-LED vertically integrated with an ITO-FGM element. The PPI of the INDIS can be further increased by reducing the channel width of the FGM and constructing vertically stacked integrated devices based on a fully transparent FGM structure.

9. The region between the neighboring Micro-LEDs is filled by the deposited of 2.1-μm SiO_2 layer. Since the SiO_2 layer is transparent. The optical crosstalk maybe a possible issue, which is visible in some subfigures in Figure 4. The impacts of planarization layer on optical crosstalk could be discussed.

Response: We thank the reviewer for this insightful comment. In our device, a 2.1 μm-thick SiO_2 layer was deposited between neighboring Micro-LEDs and planarized via chemical mechanical polishing (CMP) to achieve an ultra-flat surface, which is critical for reliable integration with the ITO/Al_2O_3/ITO floating-gate memory layers. The SiO_2 was intentionally chosen as a transparent filling material to support the dual functionality of the Integrated Nonvolatile Display–Illumination System, enabling both high-brightness pixel illumination and static image display.

We acknowledge that, due to its transparency, the SiO_2 layer may allow optical crosstalk between neighboring pixels, as observed in some subfigures of Figure 4. To address this in future designs, we plan to implement sidewall light-blocking structures, such as evaporated metal mirrors around each pixel, to suppress crosstalk while maintaining high fill-factor and optical efficiency. These strategies will further enhance contrast and overall display performance.

Reviewer #2 (Remarks to the Author):

In this manuscript, the authors described multimodal floating-gate memory driver for energy-efficient integrated display-illumination system. However, the authors should respond to the following comment prior to publication in Nature Communications. The comments are as follows:

Response: We thank the reviewer for their careful reading of our manuscript and for providing valuable comments that help us improve the quality of our work. We have carefully considered all the points raised and addressed them in detail below.

1. The gate programming voltage of ±15 V might be very high for large-scale integration and low-power systems. The authors should suggest a feasible method to reduce the operating voltage.

Response: Thanks for your insightful comment. Reducing the programming voltage of the driver circuit is of great significance for lowering power consumption and enhancing system reliability.

For floating gate memory (FGM) device, the programming voltage is closely related to the thickness and dielectric constant of the tunneling layer. In our original design, the FGM employed an 18-nm Al_2O_3 layer as the charge tunneling dielectric, which ensures low leakage and prevents image crosstalk during high-current operation of Micro-LED arrays. However, the thicker tunneling layer also requires a higher programming voltage.

To explore lower-voltage operation, we fabricated standalone FGM devices with thinner 10-nm Al_2O_3 tunneling layer. As shown in Figure R7, these devices also exhibit large memory window of 3.48 V (type-I modulation) and on/off current ratio exceeding 10^7 under a sweep voltage above -8 V to +8 V sweeping range. In type-II operation (three-terminal FGM transistor), the transfer characteristics show pronounced hysteresis with a memory window of 8.3 V and an on/off current ratio exceeding 10^8, while maintaining a threshold voltage around 3.5 V. The output characteristics under gate pulse programming (100 ms pulse width) show a maximum current of ~1.2 mA, indicating robust driving capability. As can be seen the thinner tunneling layer allows programming and erasing with voltages below 10 V, demonstrating the feasibility of low-voltage operation without compromising switching performance.

Integration of this FGM with Micro-LEDs (1M1D) confirms that both drain and gate voltage pulses effectively control the pixel current, enabling reliable switching of Micro-LEDs (Figure R8). For the full display system, a shared cathode configuration allows independent gate control of each pixel, enabling selective illumination with reduced operating voltage (Figure R9).

We note that very thin tunneling layers (e.g., 10 nm Al_2O_3) can significantly reduce the energy required for programming and erasing. It can be effectively used for the display system with low light intensity output under small driving voltage (Figure R10a_1-a_2, INDIS-1). However, when a large continuous anode voltage is applied, a large current flowing through the circuit can inadvertently activate the memory cells of unselected pixels via tunneling since all pixels are connected in a parallel configuration, leading to pattern crosstalk (Figure R10b_1-b_2, INDIS-2). Such behavior can also be reasonably understood by the retention test of the standalone FGM device operating at different anode voltage shown in Figure R11. When the voltage is higher 3.5 V, a slow increase in the output current can be clearly observed.

In contrast, the 18-nm Al_2O_3 layer used in the main manuscript provides a good balance: it suppresses leakage current (~5×10^{-13} A), prevents crosstalk, and ensures reliable high-current operation and high light intensity output (INDIS-3, Figure R10c), while still allowing programming voltages compatible with low-power operation.

In summary, reducing the tunneling layer thickness provides a feasible approach to lower the FGM programming voltage, which is suitable for display scenarios requiring shorter image retention times. In contrast, the 18-nm Al_2O_3 thickness used in our main design ensures stable, high-current operation without crosstalk, representing a practical trade-off that balances low-power operation, nonvolatile storage, and scalability for integrated display–illumination systems. **Looking forward, achieving an even lower operating voltage while maintaining robust nonvolatile storage will rely on the development of dielectrics with higher permittivity, which can provide sufficient charge confinement even at reduced thickness, enabling better equilibrium between energy efficiency and storage performance in next-generation FGM-integrated displays**.

In the revised manuscript (page 5, line 148), we have also incorporated into this flexible method of reducing the operating voltage through thinning the tunneling layer. The context as follows:

Moreover, the operation voltage and power consumption can be further optimized by employing a thinner Al_2O_3 tunneling layer. As shown in Supplementary Figure 7 and Figure 8, when the tunneling layer is reduced to 10 nm, the programing/erase voltages can be readily lowered to below 10 V. In addition, the electrical performance is presented in the Supplementary Note 2 (page 12, line 148), Supplementary Figure 7 (page 11, line 135) and Figure 8 (page 11, line 143).

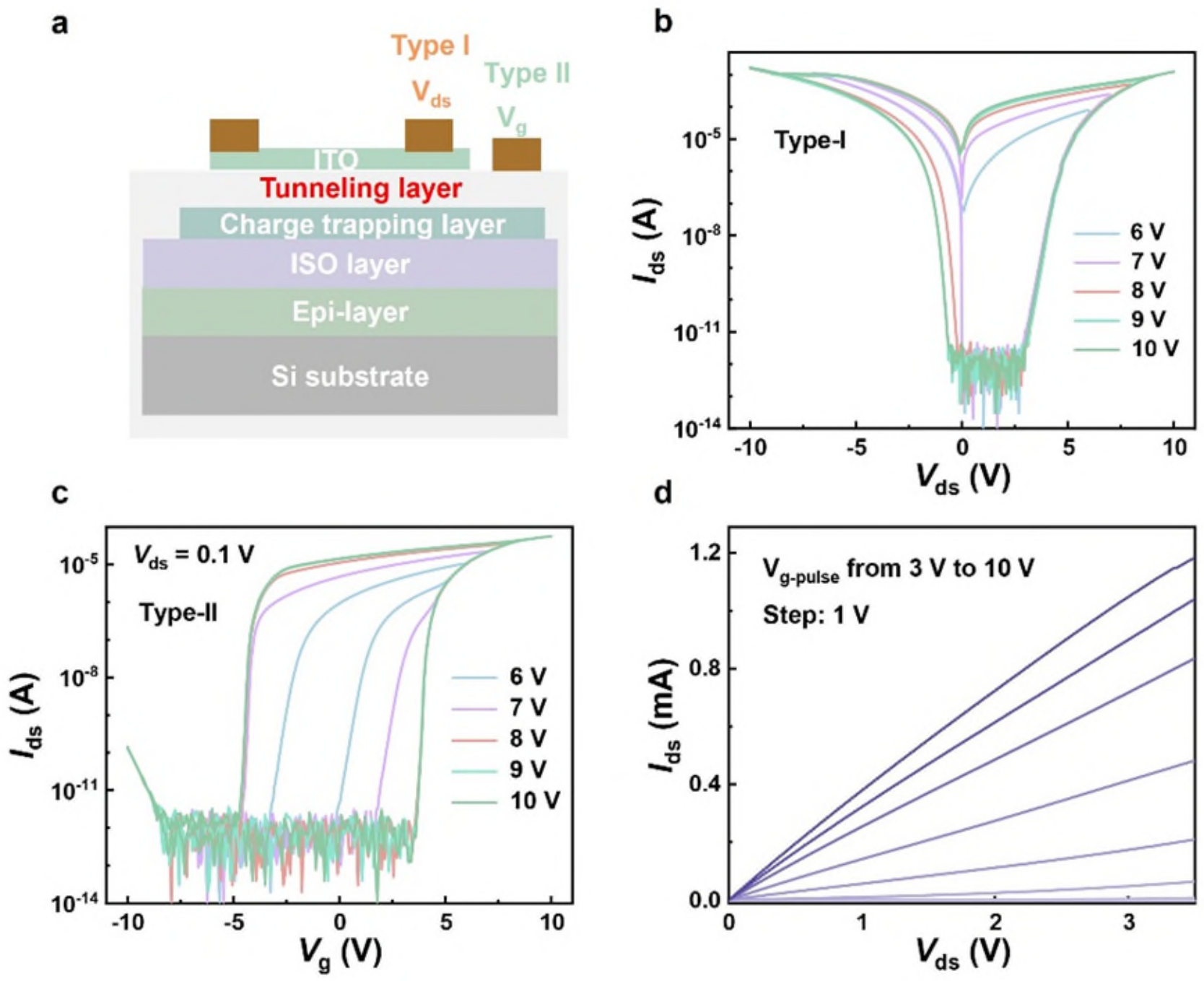

Figure R7: Electrical characterization of ITO-based floating-gate memory (FGM) devices with 10-nm gate tunneling dielectric layer. a, Cross-sectional schematic of the FGM fabricated on a GaN wafer, where the two voltage injection ports enable distinct modulation modes. **b,c,** Sweep transfer characteristics of the device controlled by drain pulse voltage from -10 V to +10 V with type-I modulation (b), and type-II modulation (c). **d,** The IV curve of the device controlled by the pulse voltage forwarded on the gate, all pulse width is one hundred milliseconds.

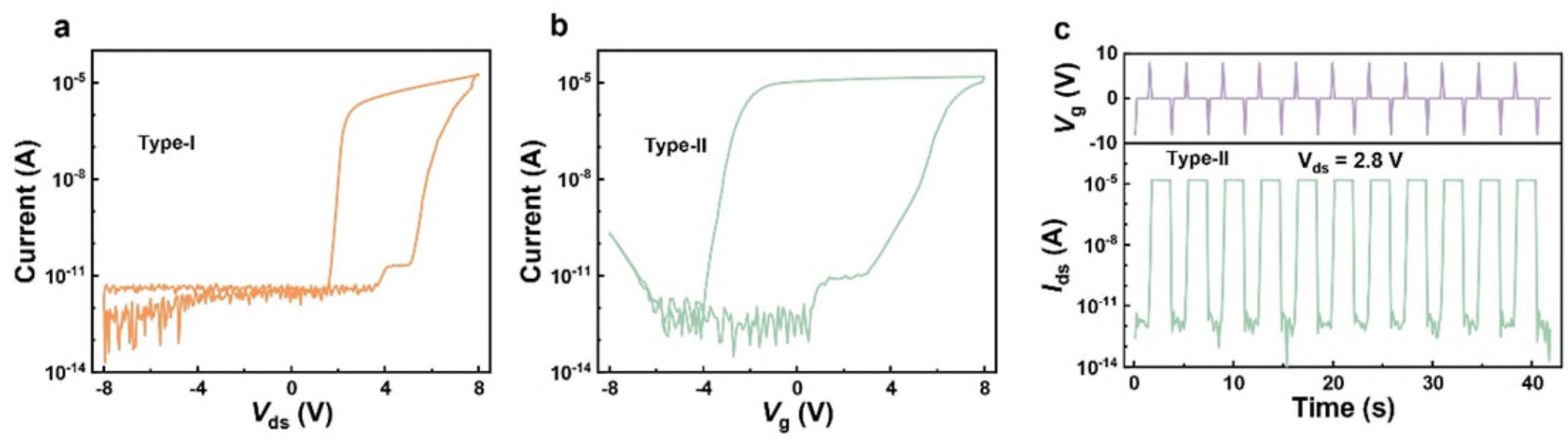

Figure R8: Individual Micro-LED integrated with ITO-based FGM, with 10-nm gate tunneling dielectric layer. a,b, The I–V characteristic of integrated device in type-I (a) and type-II (b) modulation mode, with voltage sweeping from -10 V to +10 V. c, Switching curves of the integrated device with reading voltage of 2.8 V and gate pulse width of 0.1s, respectively.

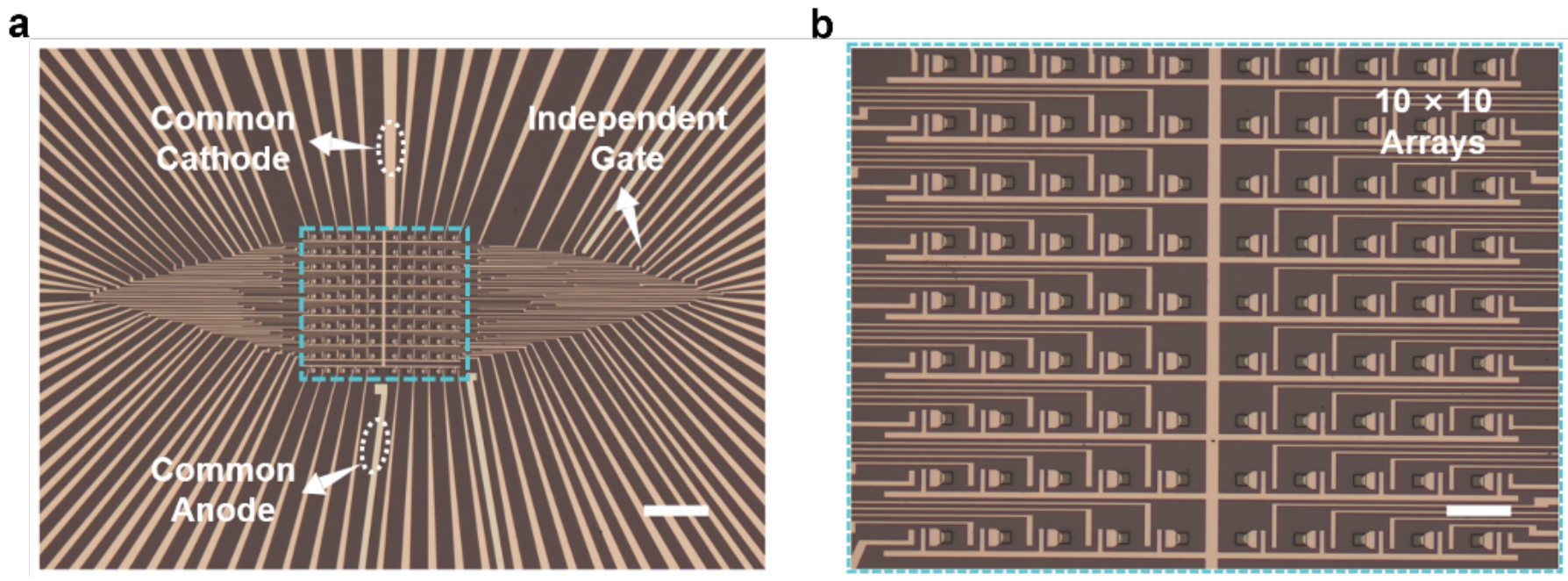

Figure R9: a, Optical micrographs of the Micro-LED arrays integrated ITO-based FGMs. Scale bars, 200 μm. **b,** Zoomed-in integration chips of the arrays. Scale bars, 100 μm.

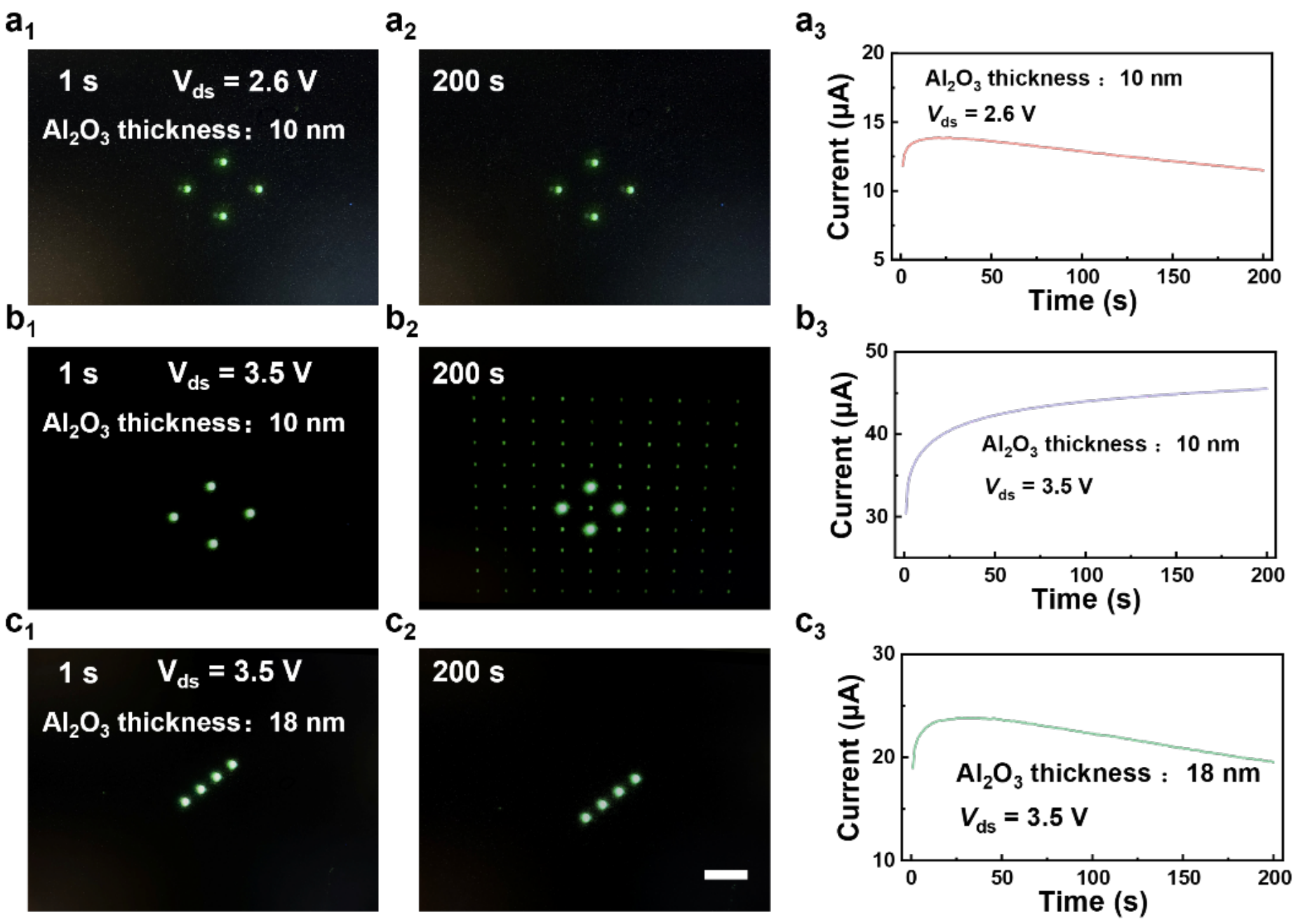

Figure R10: $\mathbf{a_1,a_2}$**,** Optical images of selectively illuminated Micro-LEDs integrated ITO-based FGMs using a 10-nm thickness Al_2O_3 tunneling dielectric layer at V_{ds} = 2.6 V. taken at 1 s (a_1) and 200 s (a_2). $\mathbf{a_3}$, Variation of Micro-LED current during continuous operation. $\mathbf{b_1,b_2}$**,** The same structure at V_{ds} = 3.5 V, taken at 1 s (b_1) and 200 s (b_2). $\mathbf{b_3}$, Corresponding current variation. $\mathbf{c_1,c_2}$**,** The integrated devices with an 18-nm-thick Al_2O_3 tunneling dielectric layer at V_{ds} = 3.5 V, taken at 1 s (c_1) and 200 s (c_2). $\mathbf{c_3}$, Corresponding current variation. Scale bars, 100 μm.

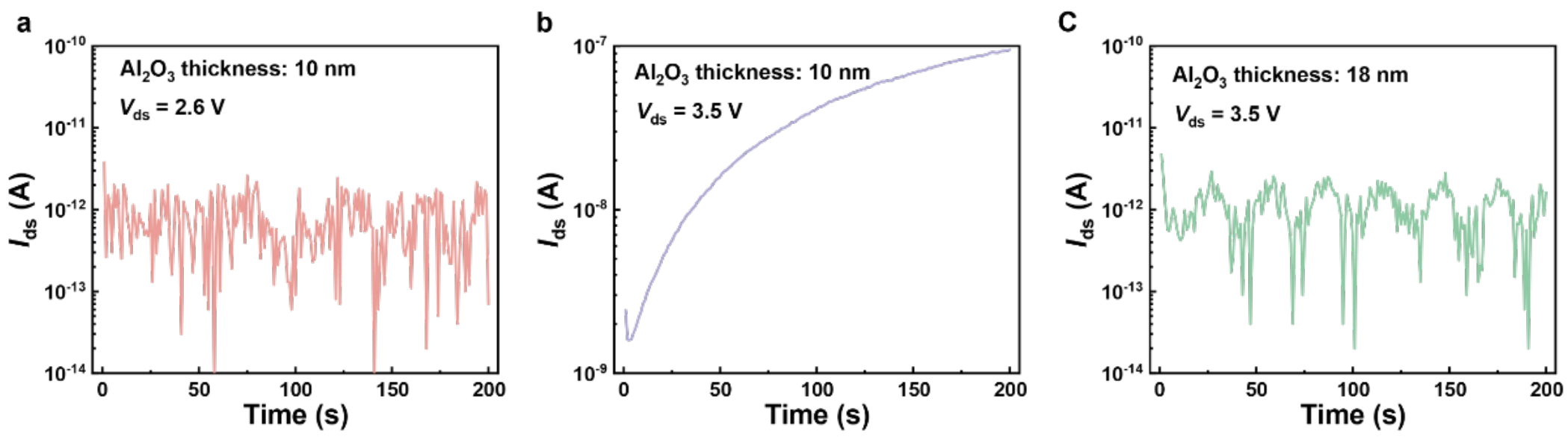

Figure R11: a,b, Variation current of FGMs using a 10-nm thickness Al_2O_3 tunneling dielectric layer at V_{ds} = 2.6 V (a) and V_{ds} = 3.5 V (b) during continuous operation. **c,** Variation current of FGMs using an 18-nm thickness Al_2O_3 tunneling dielectric layer at V_{ds} = 3.5 V.

2. The authors should describe the durability performance of the switching characteristics in a TYPE-I mode.

Response: We thank the reviewer for this valuable suggestion. According to your comment, the durability performance of the switching characteristics in type-I mode has been systematically evaluated, and the results are provided in the Figure R12 and revised Supplementary Figure 6.

As shown in Figure R12a, the device maintains a stable memory window (~7 V) and a high on/off current ratio (~10^7) over 200 continuous switching cycles, without noticeable degradation or threshold voltage shift. Furthermore, extended endurance testing up to 4,000 program–read–erase–read cycles (±12 V pulse, 0.1 s; read at 0.1 V) demonstrates that the read current remains stable at ~6.3 μA with a small fluctuation (±1.41 μA), while preserving a consistent on/off ratio (Figure R12b).

These results demonstrate the robust endurance and stable switching behavior of the device in type-I mode, which are essential for reliable and energy-efficient illumination applications. Together with the results of type-II mode, they are presented in the Supporting Figure 10 (page 14, line 182).

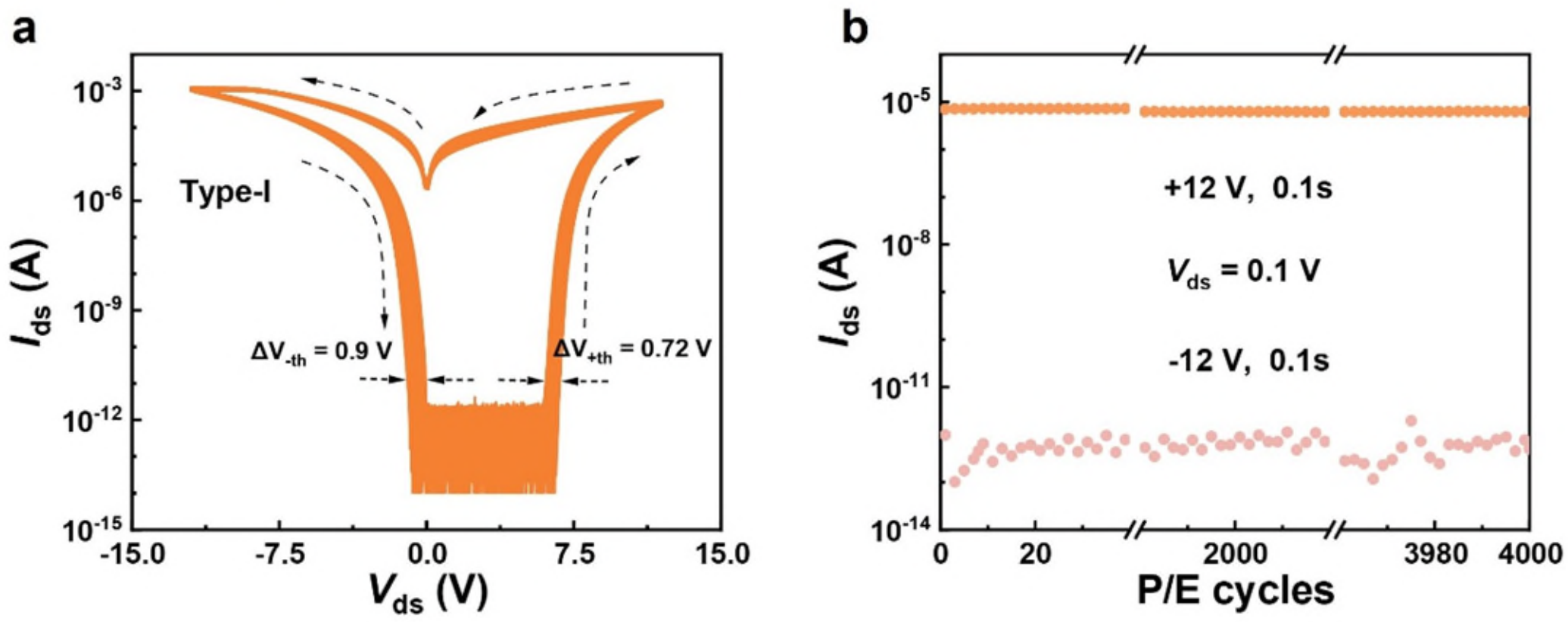

Figure R12 (Revised Supplementary Figure 6): Electrical stability and endurance of the FGM device in type-I mode. a, Transfer characteristics of the same FGM device measured continuously over 200 cycles. **b,** Endurance performance under alternating voltage pulses (V_g = +12 V/–12 V, pulse width = 0.1 s), with I_{ds} read at V_{ds} = 0.05 V and V_g = 0 V.

3. The authors should describe the distribution of programming/erase threshold voltages during multiple switching cycles to explain the reliability of the device.

Response: We thank the reviewer for this insightful suggestion. The distribution of programming and erase threshold voltages during repeated switching cycles has been analyzed to evaluate device reliability.

In type-I mode, the absolute values of both positive and negative threshold voltages exhibit a slight increasing trend over cycling (Figure R13a and R13b). Notably, the overall variation is well confined within ~1 V. Statistical analysis shows that the threshold voltages follow a near-Normal distribution, with standard deviations of $\sigma_p \approx 0.17$ V (program) and $\sigma_e \approx 0.23$ V (erase), indicating good cycle-to-cycle uniformity (Figure R13c).

As shown in Figure R13d and R13e, in type-II mode, the negative threshold voltage remains nearly unchanged, while the positive threshold voltage shows only a slight increase (within 0.6 V, ~9.52%). The extracted distributions also exhibit narrow spreads ($\sigma \approx 0.14$ V), demonstrating stable switching behavior (Figure R13f). These values are comparable to the best reported results (Nature Electronics, 2024, 7(10): 868-875.), further confirming the robustness of our devices.

Overall, the narrow distribution and small standard deviation of threshold voltages in both modes indicate high uniformity, low variability, and excellent reliability during repeated switching cycles. And results are presented in the revised Supplementary Figure 11 (page 14, line 187).

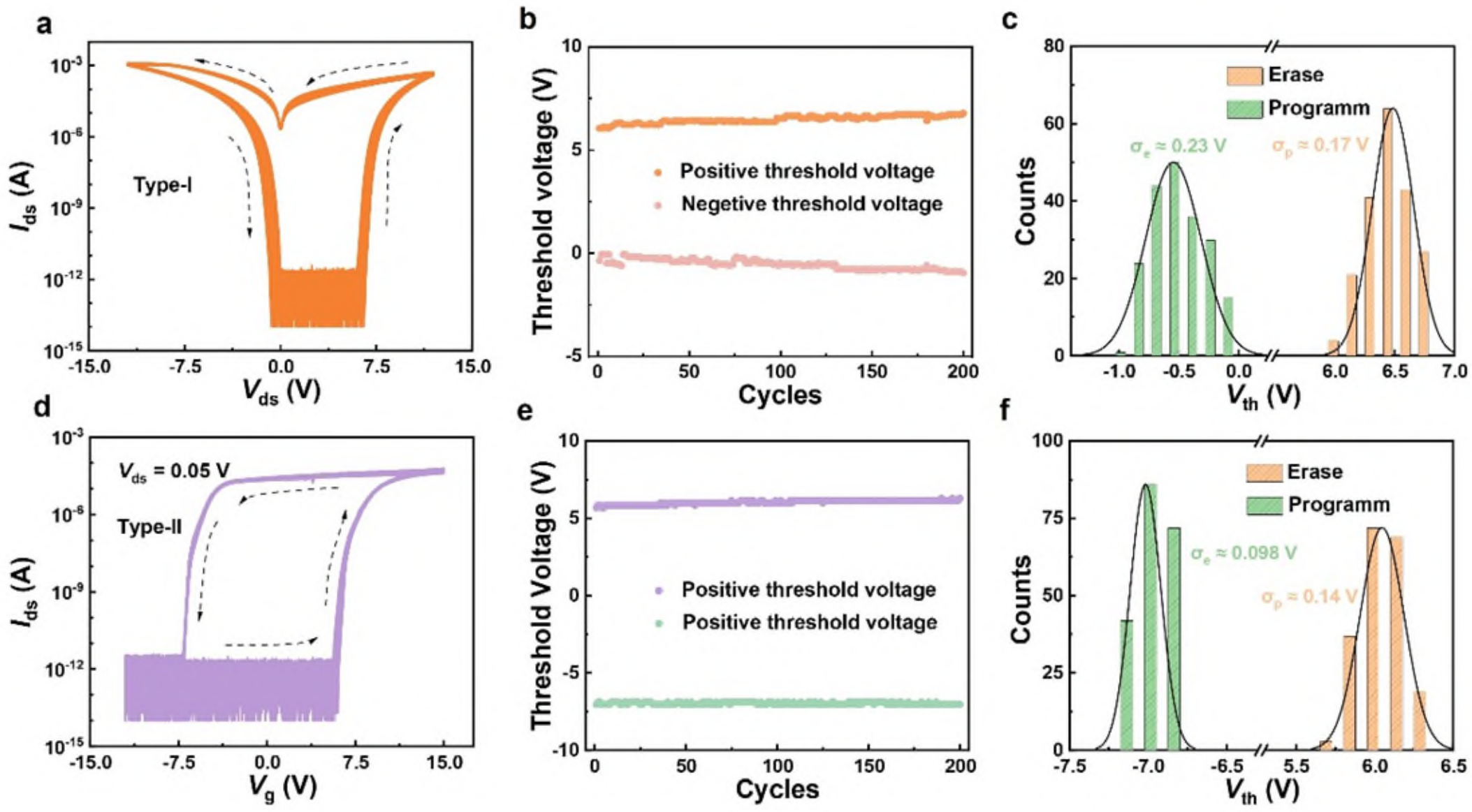

Figure R13 (Revised Supplementary Figure 7): In type-I mode a, Summary of transfer characteristic curves from continuous testing. **b,c,** along with the extracted threshold voltage variation (b) and its statistical distribution (c). **In type-II mode d,** Summary of transfer characteristic curves from continuous testing. **e,f,** along with the extracted threshold voltage variation (e) and its statistical distribution (f).

4. The authors should describe the effect of the current level in the high-resistivity state for the switching characteristic test on the system noise.

Response: We thank the reviewer for this valuable suggestion. The current level in the high-resistivity state (HRS) is closely related to system noise and thus directly affects the readout reliability. We have analyzed this effect and added discussion in the revised manuscript.

Firstly, to characterize the intrinsic noise of the measurement system, a dummy transfer measurement was performed by lifting the tungsten probe in the B1500 system (equipped with a Keithley source) so that it was in contact with air, ensuring a high-resistance condition. The resulting measured current represents the baseline system noise, which was around ~10^{-13} A.

Subsequently, the transfer characteristics of the fabricated FGM device were measured under the same conditions. The HRS current of the device remained at approximately the same level (~5×10^{-13} A), very close to the detection limit of the B1500 system (Figure R14). This demonstrates that the high-resistivity state current of our device is extremely low and contributes negligibly to system noise.

As a result, the low and stable HRS current ensures that the signal from the low-resistivity state

(LRS) is clearly distinguishable, producing a high on/off ratio (~10^7) and a high signal-to-noise ratio, which is critical for reliable switching and stable illumination of Micro-LED pixels.

Such an excellent high-resistivity state can be reasonably attributed to the wide bandgap of the ITO channel material, which provides excellent off-state characteristics and effectively suppresses leakage current. Additionally, the device structure and high-quality tunneling dielectric further ensure stable HRS operation. This low dark current guarantee reliable switching performance with minimal interference from measurement noise.

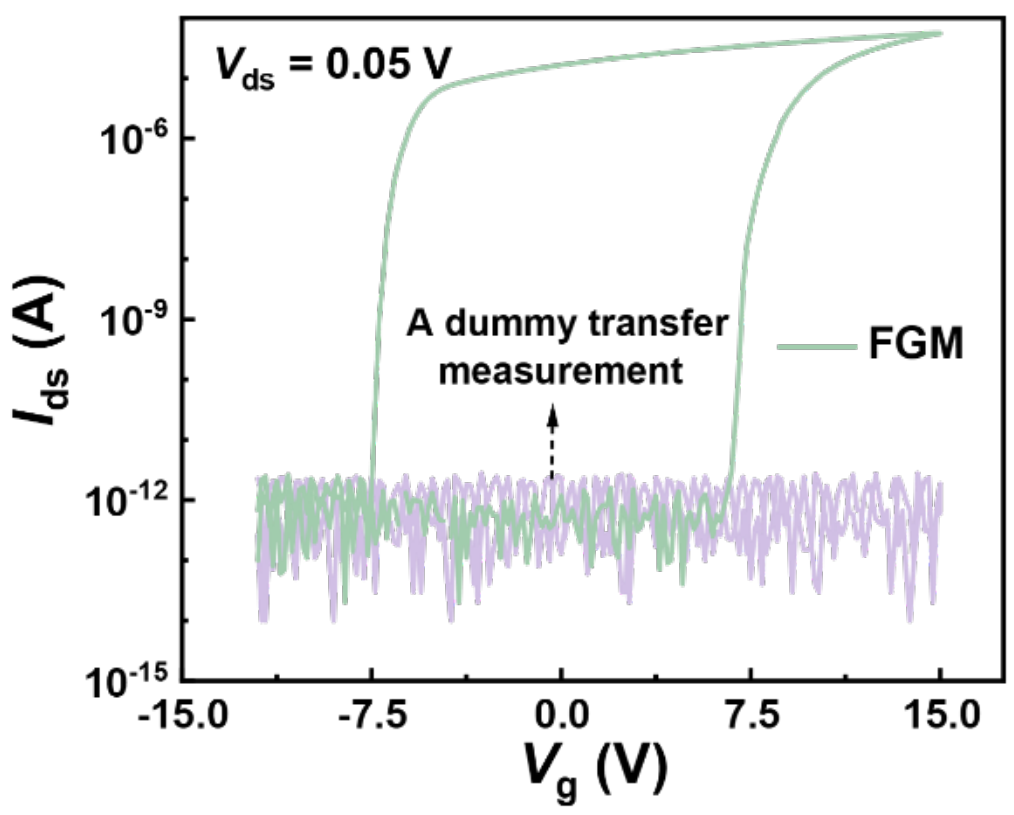

Figure R14: Sweep transfer characteristics of the FGM device and dummy state controlled by gate pulse voltage from -10 V to +10 V with type-II modulation

5. The authors should describe whether it is necessary to reset the device's conductivity state during the transition between multiple operating modes.

Response: We thank the reviewer for this insightful comment. A consistent initial resistivity state of the FGM devices is important for ensuring reliable operation. In our experiments, we applied an initialization step before each illumination operation to guarantee a uniform starting point, achieved by applying a negative pulse (e.g., −12 V, 0.1 s) to reset the device into a high-resistivity state.

During transitions between different operating modes, we presented the switching behavior without applying this initialization step in Figure R15. The results show that the device can achieve smooth and stable switching between modes, with well-maintained electrical characteristics. Similar behavior is observed in the integrated Micro-LED system, indicating good interface compatibility and robust operation under practical conditions.

Therefore, in controlled measurements (e.g., evaluating the effects of pulse width and voltage on memory performance), we employ initialization to ensure consistency and comparability. However, in practical applications, initialization is not strictly required for every mode

transition. Depending on the operation requirements, the system can either: (i) perform a global reset to ensure uniform brightness across all pixels (e.g., switching from local illumination to full illumination), or (ii) directly switch between modes without reset while maintaining stable operation.

We have specifically marked this point in the revised manuscript (page 6, line 169) to help readers better understand the switching mechanism of the device. The context as follows:

Notably, initialization (reset the device's conductivity state) is not strictly required for every mode transition, and there is negligible interference between Type-I and Type-II modes.

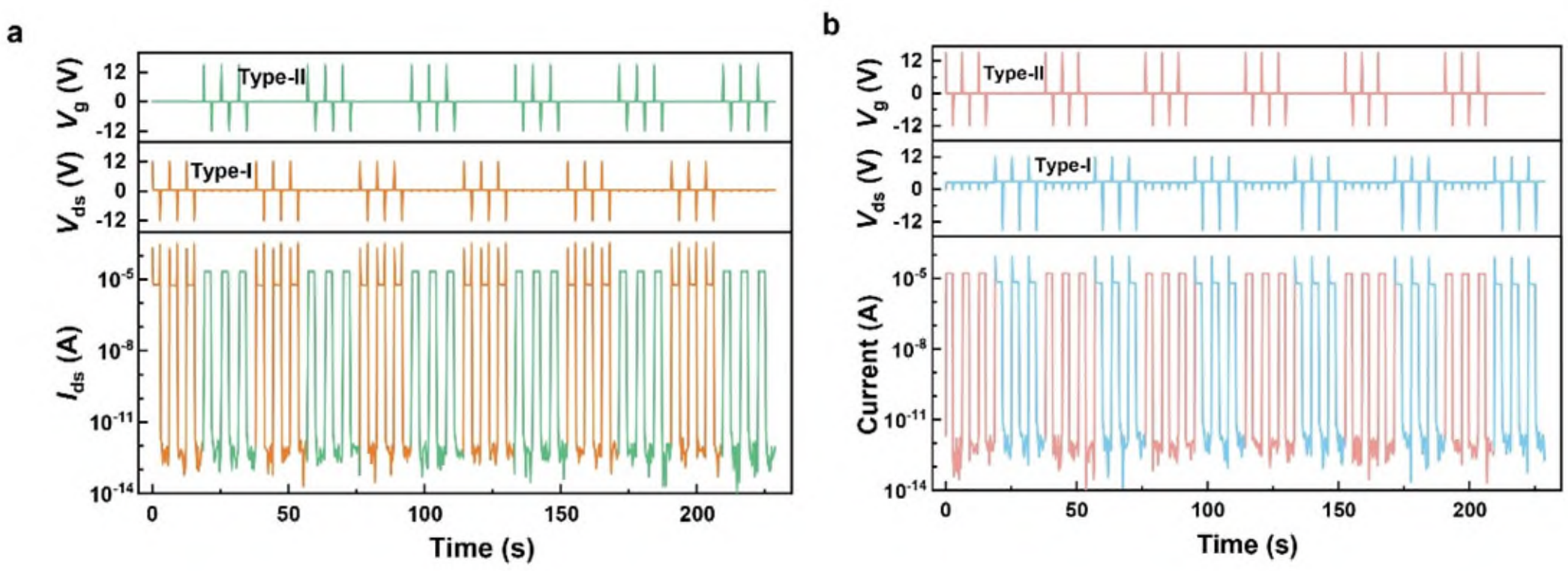

Figure R15: a,b, The switching performance of the device between the type-I and type-II modulation modes. Standalone FGM device (a), Micro-LED integrated FGM device (b).

6. The authors should describe the possibility of the interference between type-I and type-II modes.

Response: We thank the reviewer for this insightful comment. The potential interference between type-I and type-II modes has been carefully evaluated.

In our device, the two modes operate through distinct charge injection pathways and electrodes: type-I mode relies on drain-induced charge injection for illumination, while type-II mode utilizes gate-controlled modulation for single-pixel operation. This separation effectively suppresses direct electrical interference between the two modes.

Experimentally, both modes exhibit stable switching characteristics and high on/off ratios during multimodal operation (Figure R15). Importantly, the two modes also **play complementary roles**: type-I mode enables global control over the entire device array, which can be used for uniform initialization or large-area illumination, while type-II mode allows independent control of individual pixels, enabling fine tuning or performance compensation for selected devices during operation. As a result, the two modes can be flexibly configured to operate either independently or cooperatively, depending on application requirements.

These results demonstrate that interference between type-I and type-II modes is negligible, while their independent controllability and cooperative functionality offer significant advantages for integrated display–illumination systems. We have added this annotation in the revised manuscript (page 6, line 170), the context as follows:

Notably, initialization (reset the device's conductivity state) is not strictly required for every mode transition, and there is negligible interference between Type-I and Type-II modes.

7. The authors should describe how the Micro-LEDs experience heat buildups under high-brightness operating conditions.

Response: We thank the reviewer for this valuable comment. Heat buildup under high-brightness operation is a critical issue for Micro-LEDs, as insufficient thermal management can lead to junction temperature rise, resulting in luminance degradation and reduced device reliability.

To address this, the device is designed with a bottom metal bonding layer, which serves as an efficient heat dissipation pathway to conduct heat away from the active region.

To evaluate the thermal stability, Figure R15a displays long-term continuous operation tests carried out under different brightness conditions. When operated at a luminance of 6.81×10^6 cd/m^2 (3 V bias), the device exhibits only ~5.76% luminance degradation after 3.6×10^3 s of continuous operation. And there are almost no degradations under lower brightness conditions (3×10^6 cd/m^2 (2.6 V bias), 0.2×10^6 cd/m^2 (2.3 V bias)).

These results indicate that the device can sustain stable operation under high-brightness conditions without significant irreversible degradation, demonstrating effective thermal management enabled by the device structure, where heat generated from the chip is timely conducted away via the bottom bonding metal (Figure R16b), ensuring reliable operation. In the future, we will further optimize thermal management and focus on Micro-LED reliability under high-temperature and high-humidity environments.

We have added this discussion in the revised manuscript (page 7, line 198), Supplementary Note 3 (page 20, line 238) and Supplementary Figure 19 (page 20, line 242).

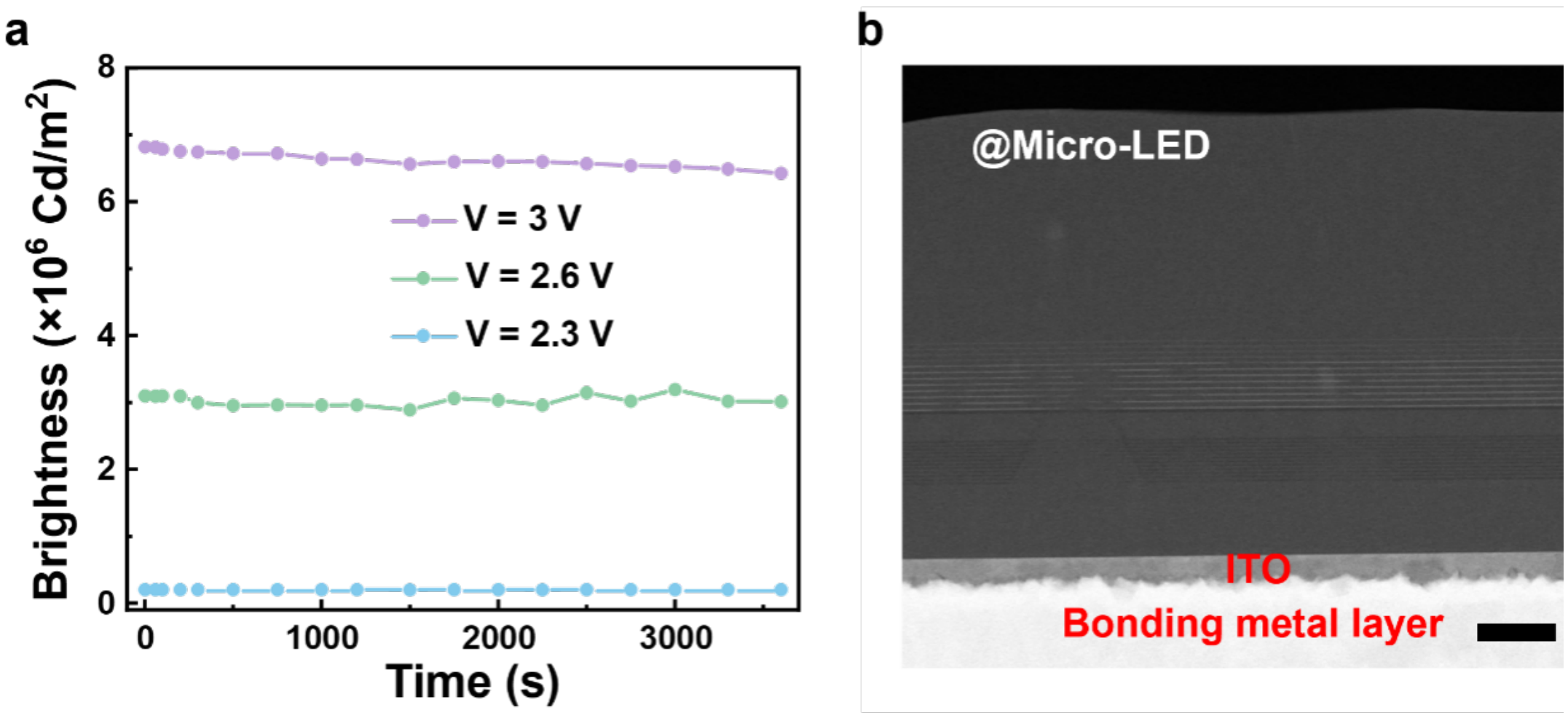

Figure R16: a, The variation of Micro-LED brightness lit up under different conditions. **b,** The cross-section of Micro-LED epitaxy layers. Scale bars, 500 nm.

8. The authors should describe at least a table about comparison of the low power consumption and stability characteristics of the device studied in this work with those of the devices published in other literatures.

Response: We thank the reviewer for this valuable suggestion. We agree that a quantitative comparison of the power consumption and stability characteristics between our device and similar devices from the literature will significantly improve the clarity and impact of our work. Owing to the Micro-LED integrated with non-validated floating gate memory for energy-efficiency display system was first presented in our work. Hence, table 1 summary the Micro-LED / Micro-OLED devices integrated with in-memory transistors, memristors or ferroelectric transistors to reduce energy consumption for static display, and traditional thin film transistors. Key metrics of low-power operation (e.g., operating voltage, leakage current, on/off current ratio, switch power consumption per operation) and stability (e.g., retention time, endurance) of our device with those of recently reported devices were presented in Table 1.

Compared to Micro-LED integrated with in-memory transistors, although the lower leakage current was achieved, its operation voltage of 35 V was much higher than that of our device, resulting in higher switch energy per operation. Furthermore, the static retention ability was limited by a retention time of 60 seconds, and the endurance was poor.

Compared to Micro-LED integrated with advanced memristor, despite their low operating voltage and good stability, the current in the program state can be extremely large due to their two-terminal structure, leading to a power consumption as high as the nJ level—nearly four orders of magnitude higher than the energy consumption per operation of our device.

Compared to Micro-LED integrated with ferroelectric transistors, they offer advantages in terms of leakage current and endurance; however, their retention capability is poor, with a maximum retention time of only 100 seconds—much lower than that of our developed device.

Compared to Micro-LED integrated with traditional thin film transistors, which require a continuous power supply, the energy consumption from gate would be up to 10^3~10^5 pJ if continuously lit up for five minutes. And our developed integrated device would exhibit unparalleled advantages in power consumption owing to its low leakage current, excellent retention capability, and superior endurance, no additional gate power is required after a single programming operation (11.43 pJ and 3.07 pJ).

In summary, our developed integrated device offers advantages in terms of energy consumption and stability over novel static display systems, and exhibits significantly lower power consumption compared to traditional thin-film transistor driving technology.

We have added this table in the Supplementary information (page 33).

Tabel R1 Summary of the power consumption and stability in integrated Micro-LED devices with various driver technology.

Display type	Driver type	Voltage	Leakage current	On/off current ratio	Switch energy	Retention	Endurance	Reference
Micro-LED	In-memory transistors	-35	$>10^{-12}$	$<10^{7}$	17.5 pJ	60	1000	1
Micro-LED	Memristor	4.3	10^{-8}	$<10^{6}$	~ 10^{6} pJ	$>10^{4}$	> 480	2
Micro-LED	Memristor	0.17	10^{-9}	$<10^{7}$	2×10^{5} pJ	$>10^{4}$	10000	3
Micro-LED	Fe-MFFT	8	10^{-14}	4×10^{8}	Sub-pJ	50	10^{8}	4
Micro-LED	Fe-TFT	6	$>10^{-13}$	$<10^{8}$	Sub-pJ	100	10^{8}	5
Micro-OLED	Fe-OLET	60	$>10^{-10}$	$<10^{7}$	>300 pJ	10^{4}	>100	6
Micro-LED	MoS_2- TFT	6.2	6.41×10^{-13}	$<10^{8}$	1192 pJ (for 5 min)	NA	NA	7
Micro-LED	Poly-Si TFT	8	5×10^{-11}	$<10^{8}$	1.2×10^{5} pJ (for 5 min)	NA	NA	8
Micro-LED	ITO-based TFT	8	5.33×10^{-12}	$\sim10^{7}$	11560 pJ (for 5 min)	NA	NA	9
Micro-LED	ITO-FGM	15	7.61×10^{-12} A	$>1.8\times10^{8}$	11.43 pJ	6000	>4000	This work
Micro-LED	ITO-FGM	8	3.84×10^{-12} A	$>1.2\times10^{8}$	3.07 pJ	6000	>8000	This work

Reviewer #3 (Remarks to the Author):

This manuscript reports a micro-LED platform that integrates display and lighting functions using nonvolatile operation. The demonstration of monolithic integration at the 4-inch wafer scale with high yield is a notable strength and indicates a mature fabrication process with good scalability. Nevertheless, several key aspects of the device operation and the claimed conceptual novelty are not sufficiently clarified or supported by quantitative evidence. Addressing these issues would substantially improve the technical rigor and overall impact of the work. The following major and minor comments are provided to help strengthen the manuscript.

Response: We sincerely thank the reviewer for the careful evaluation and constructive comments. We are encouraged that the reviewer recognizes the significance of our work, particularly the monolithic integration of display and lighting functions at the 4-inch wafer scale with high yield, which reflects the scalability and maturity of the fabrication process.

We also appreciate the reviewer's insightful suggestions regarding the clarification of device operation and the need for more quantitative analysis to support the conceptual novelty. In response, we have carefully revised the manuscript to provide a more detailed description of the operating mechanisms, strengthened the quantitative characterization, and clarified the key distinctions and advantages of the proposed approach.

All specific comments have been addressed point-by-point below, and the corresponding revisions have been incorporated into the manuscript. We believe these improvements have significantly enhanced the technical rigor and clarity of the work.

Major comments

1. Type-I operation mechanism and charge injection process

In the type-I operation mode, program/erase is achieved via drain–source biasing, but the physical role of the tunneling dielectric (Al_2O_3) is not clearly explained. It is unclear whether the gate electrode is electrically floating and how a sufficient electric field is established across the tunneling dielectric using drain bias alone to induce hole injection into the charge trapping layer. Furthermore, although the charge injection mechanism is described as Fowler–Nordheim tunneling, the reported Al_2O_3 thickness (~18 nm) is relatively large compared to conventional FN-based floating-gate memories. Clarification is needed as to whether FN tunneling is indeed dominant or whether other mechanisms, such as trap-assisted tunneling, may contribute. A clearer description of the charge injection pathways, electric field distribution, and the distinct roles of the gate and dielectric in type-I and type-II modes is required. Energy band diagrams

and quantitative discussion of the electric field conditions would be highly beneficial.

Response: We thank the reviewer for this insightful comment regarding the type-I operation mechanism and the role of the tunneling dielectric.

In type-I mode, as shown in Figure R17, sweep transfer characteristics of the FGM device were conducted controlled by drain pulse voltage from -12 V to +12 V under gate floating and grounded, respectively, showcasing no obviously distinction, and indicating program and erase are achieved via a drain–source bias applied to the FGM device, while the gate electrode is electrically floating. The floating gate forms a capacitive coupling with both the drain and source, allowing a sufficient local electric field to be established across the Al_2O_3 tunneling dielectric. This field enables holes to tunnel into the charge trapping layer, primarily via Fowler–Nordheim (FN) tunneling.

Although the Al_2O_3 thickness (~18 nm) is relatively large, FN tunneling remains the dominant mechanism due to the high local electric field induced by the drain bias on the floating gate. We also validated this mechanism by fabricating devices with a thinner 10 nm Al_2O_3 tunneling layer, which showed a lower program/erase voltage while exhibiting the same memory behavior (Figure R18), consistent with FN-dominated injection. Similar two-terminal storage behavior has also been reported in literature (Nature Communications, 2016, 7:12725; Adv. Mater. 2017, 29, 1703363), confirming the physical plausibility of the mechanism. And Figure R18b depict that leakage currents between the gate and source are both below 10^{-11} A, which is lower than typical value with trap-assisted tunneling. Furthermore, for the FGM devices with two different tunneling layer thicknesses, Figure R19 depict double-sweep transfer characteristics of the FGM device under type-II modulation. Negligible hysteresis is observed within the small voltage scanning range, further confirming that our developed FGM devices have very low defect states and that the tunneling mechanism is predominantly FN tunneling.

To clarify the charge injection pathways and the distinct roles of the electrodes, we have added energy band diagrams and schematics illustrating the electric field distribution and injection mechanism under both type-I and type-II operation (revised Figures R20 and R21). In type-I mode, during program state upon a pulse voltage of +12 V applied on to drain electrode (Figure R20-I), a large potential difference and hence a strong electric field was established between drain and floating gate (ITO). This induces band bending in the Al_2O_3 tunneling layer, allowing holes to tunnel through the narrow barrier region into the ITO floating gate layer with FN tunneling. At the state of read upon program operation (Figure R20-II), the holes stored in the floating gate layer due to the energy band of Al_2O_3 return to its normal state after withdrawing program voltage, which would generate electrons in the ITO channel and cause the conduction band of the material to lie lower than the potential of the metal electrode. And after a small reading voltage of 0.1 V applied, the channel turns on, exhibiting a large on-current (>10 μA). The drain bias drives charge injection while the gate remains floating (FG), enabling global

pixel illumination. During erase state upon a pulse voltage of -12 V applied on to drain electrode (Figure R20-III), similarly, electrodes would be tunneled through the narrow barrier region into the ITO floating gate layer. The stored electrodes further generate holes the channel region and cause the conduction band of the material to bend upward, creating a potential barrier between the metal electrode and ITO channel (Figure R20- IV). When a read voltage of 0.1 V applied, the channel turns off, exhibiting a low off-state current (~5×10^{-13} A). Such the drain bias drives charge injection while the gate remains floating, enabling global pixel illumination. In type-II mode, as shown in Figure R21, the program/erase pulse voltage is applied onto the gate electrode, where holes would be also tunneled through the narrow barrier region into the ITO floating gate layer with FN tunneling. Similarly, after a small reading voltage of 0.1 V applied, the channel turns on (upon program operation), or off (upon erase operation). The voltage applied onto drain can be significantly reduced, leading to lower current during program and erase operation. And the gate is actively biased to selectively program or erase individual pixels, providing independent pixel control. The Al_2O_3 layer serves as the tunneling barrier, regulating the injection rate and preventing leakage, ensuring stable operation under high-current conditions.

These experimental results, together with the energy band diagrams and mechanistic schematics, provide a clear and quantitative understanding of the physical processes governing both type-I and type-II operation. We have marked this reminder in the revised manuscript (page 5, line 129 and line 145), and a detailed energy band diagrams would be presented in the Supporting Figure 4 (page 8, line 119) and Figure5 (page 10, line 129).

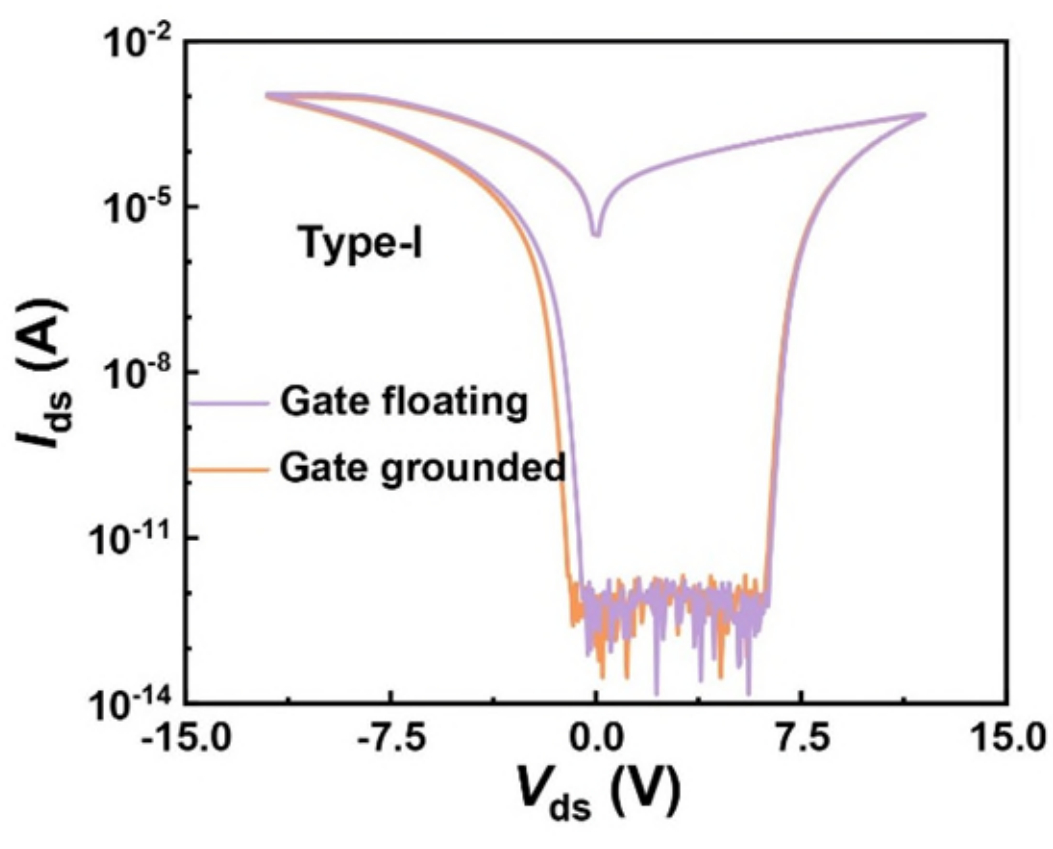

Figure R17: Sweep transfer characteristics of the FGM device controlled by drain pulse voltage from -12 V to +12 V with type-I modulation under gate floating and grounded.

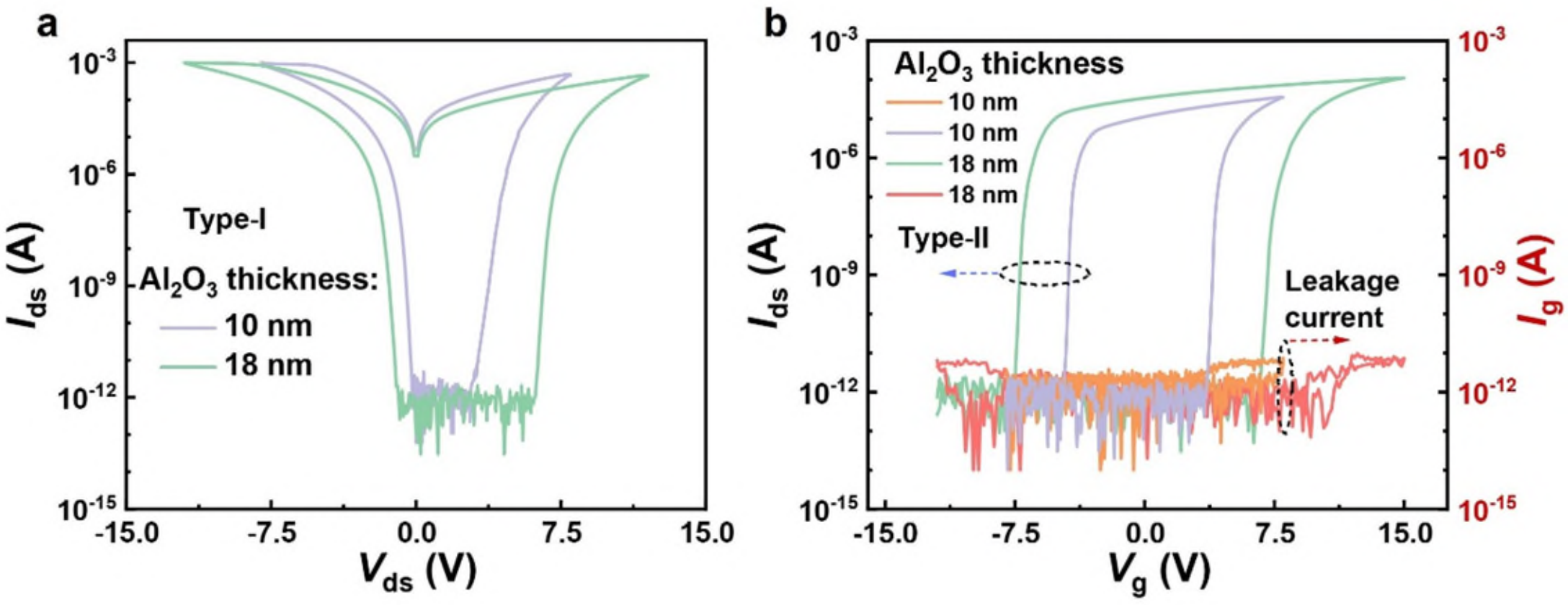

Figure R18: Sweep transfer characteristics of the FGM device controlled by drain pulse voltage from -12 V to +12 V with type-I modulation under gate floating and grounded.

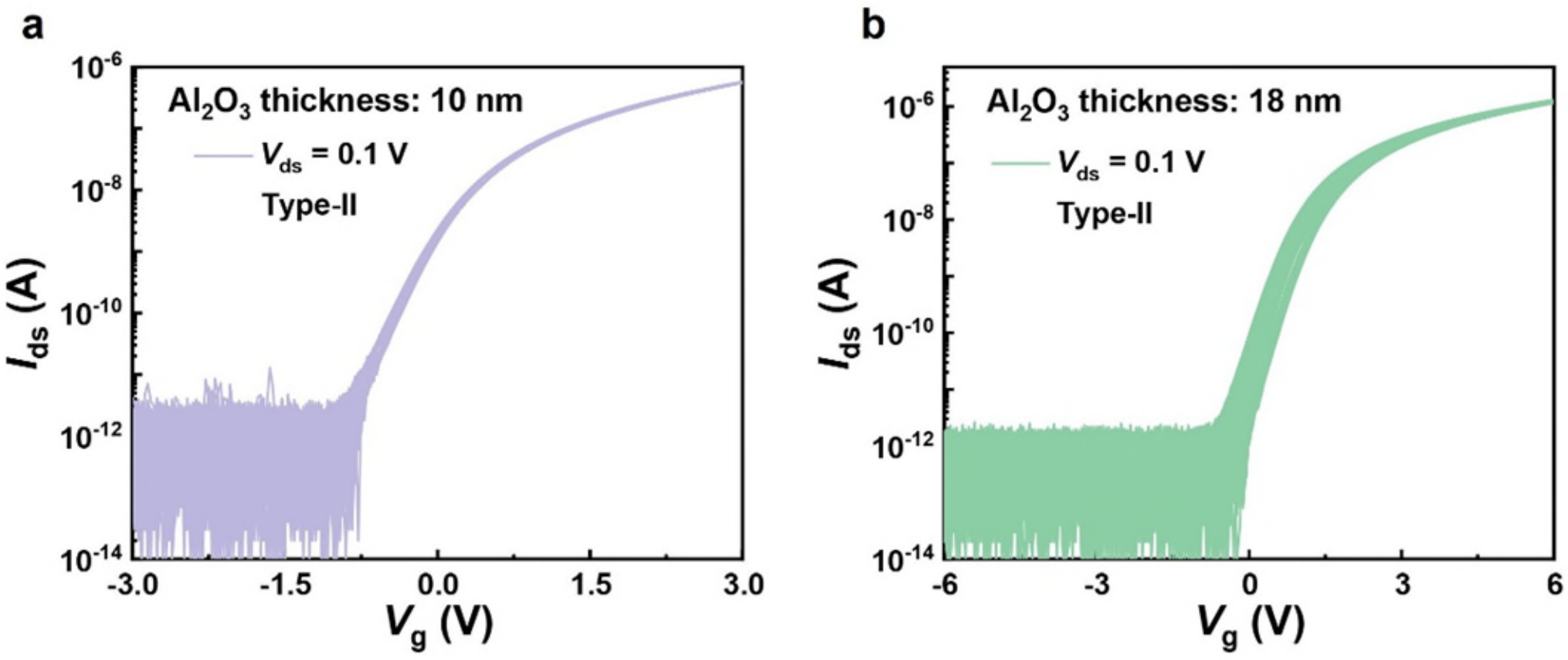

Figure R19: a,b, Sweep transfer characteristics of the FGM device with type-II modulation, controlled by gate pulse voltage from -3 V to +3 V for thinner Al_2O_3 thickness FGM (a), and from -6 V to 6 V for thicker Al_2O_3 thickness FGM (b).

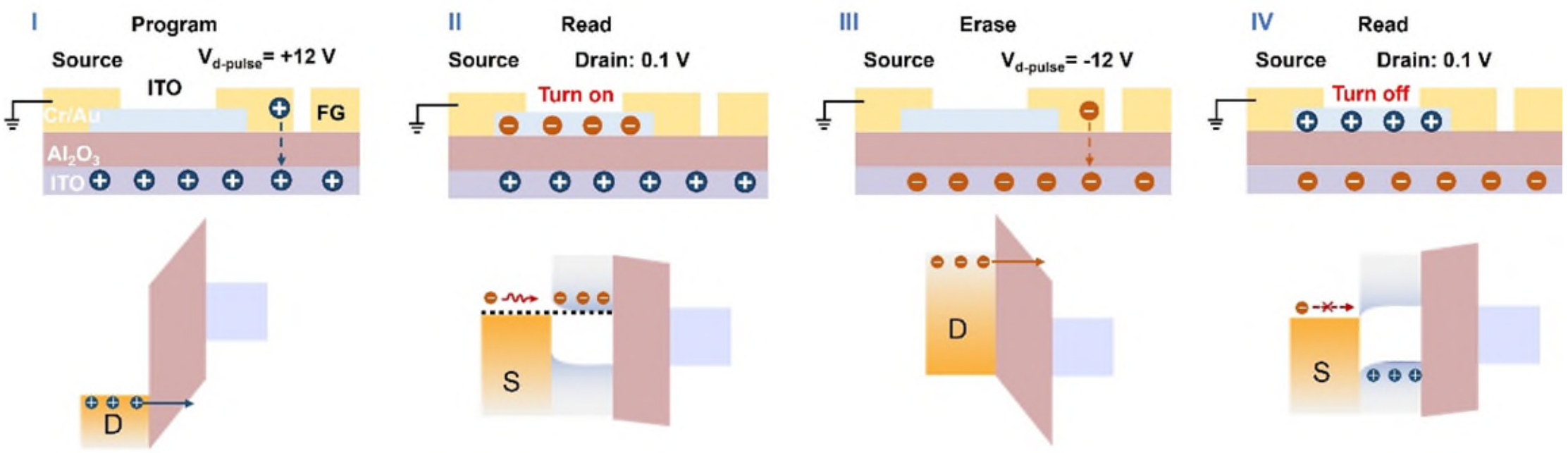

Figure R20: Schematic illustration of memory operation and band diagram under type-I operation mode. At the state of program (I), read upon program (II), erase (III), and read upon erase (IV).

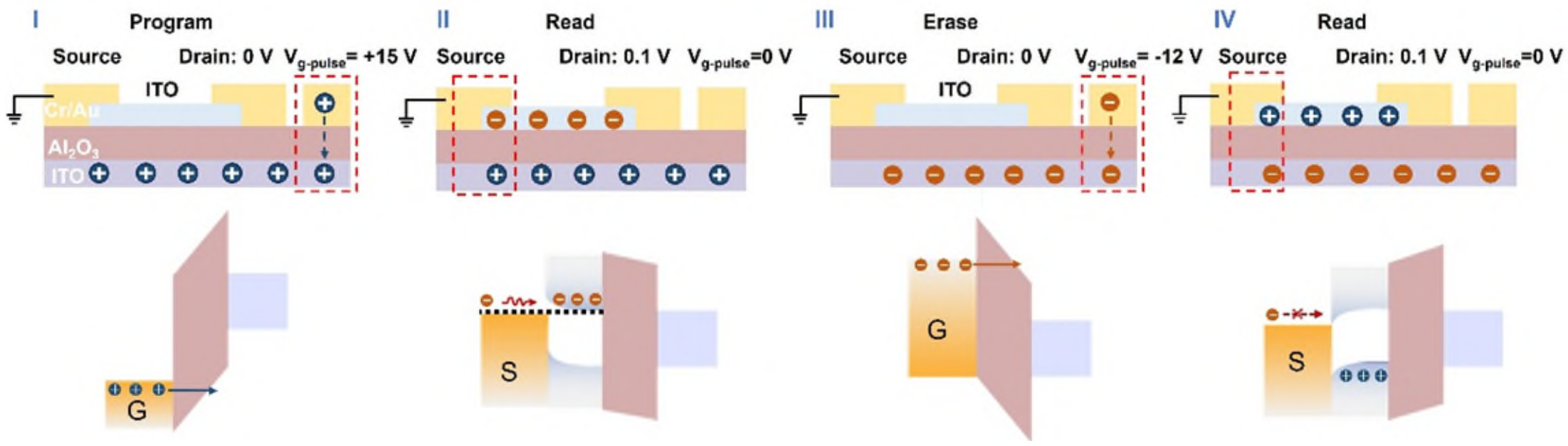

Figure R21: Schematic illustration of memory operation and band diagram under type-II operation mode. At the state of program (I), read upon program (II), erase (III), and read upon erase (IV).

2. Evaluation of nonvolatile operation

Although nonvolatile operation is a central claim of this work, its evaluation remains limited. The basis for selecting a retention time of 2000s is not defined, for example in terms of an acceptable percentage loss in optical output. It is also unclear whether longer-term retention measurements are feasible. Moreover, retention behavior is evaluated primarily using optical microscopy images, which provide only qualitative information. Quantitative metrics, such as the percentage change in optical output or luminance over time, should be provided to more convincingly substantiate the nonvolatile operation claim.

Response: Thanks for the reviewer's insightful comment. The nonvolatile operation of the Micro-LED–FGM integrated system is indeed a central claim, and we have expanded our evaluation with more quantitative metrics.

Retention time selection and measurement. In our experiments, a retention time of 2000 s was initially chosen as a practical timescale to evaluate stable optical output under continuous operation. To further assess nonvolatility, measurements were extended up to 6000 s (~1.7 hours). Both type-I and type-II modes were evaluated. During this period, the on-state current in type-I mode gradually decreased by about 10.1 %, while the off-state current remained at approximately 10^{-13} A, maintaining a high on/off ratio above 10^7. Similarly, for type-II mode, the off-state current remains stable at 10^{-13} A and the on/off ratio consistently above 10^8.

These results demonstrate that the FGM devices exhibit robust nonvolatile operation in both modes, with only minor variations over long-term operation. And it would be provided in the revised manuscript (page 6, line 159) and Supplementary Figure 12 (page15, line 192).

Retention performance of the integrated system. To further evaluate the nonvolatile operation in the fully integrated display–illumination system, we complemented the retention tests by recording the actual luminance of programmed Micro-LED pixels at selected time points (program condition: $V_{\text{g-pulse}}$ = 15 V, pulse width of 1 s). Over the retention period (3600 s), the measured luminance decreased by only about 9.4 %, exhibiting an excellent retention performance.

These results are consistent provide additional quantitative support for the stable nonvolatile operation of the FGM-driven Micro-LED arrays.

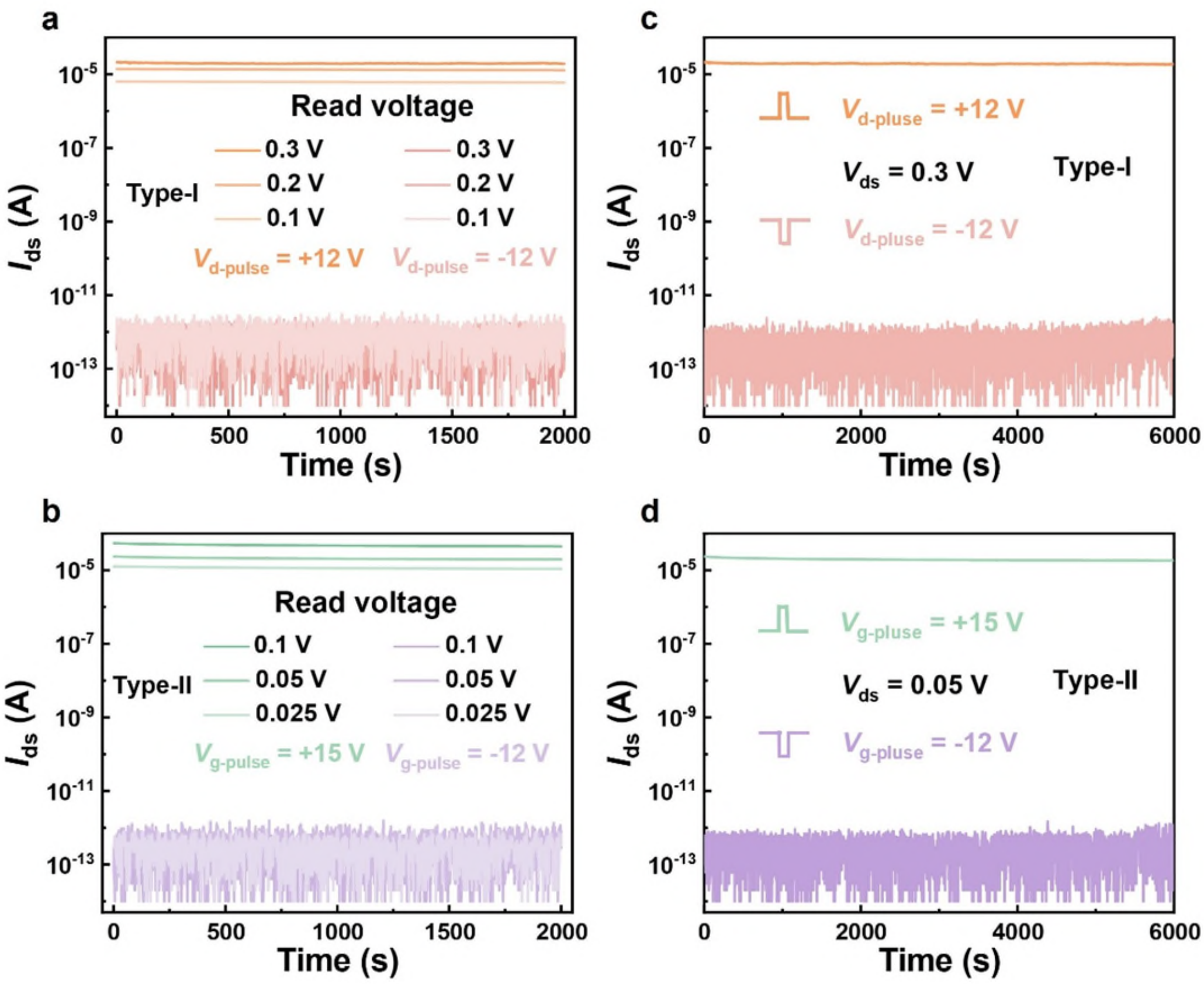

Figure R22: Retention performance of FGM devices. a,b, Within a 2000-second range under different reading voltages: type-I mode (**a**) and type-II mode (**b**), respectively. **c,d,** Long -term range under reading voltage of 0.3 V for type-I mode (**c**) and 0.05 V with type-II mode (**d**).

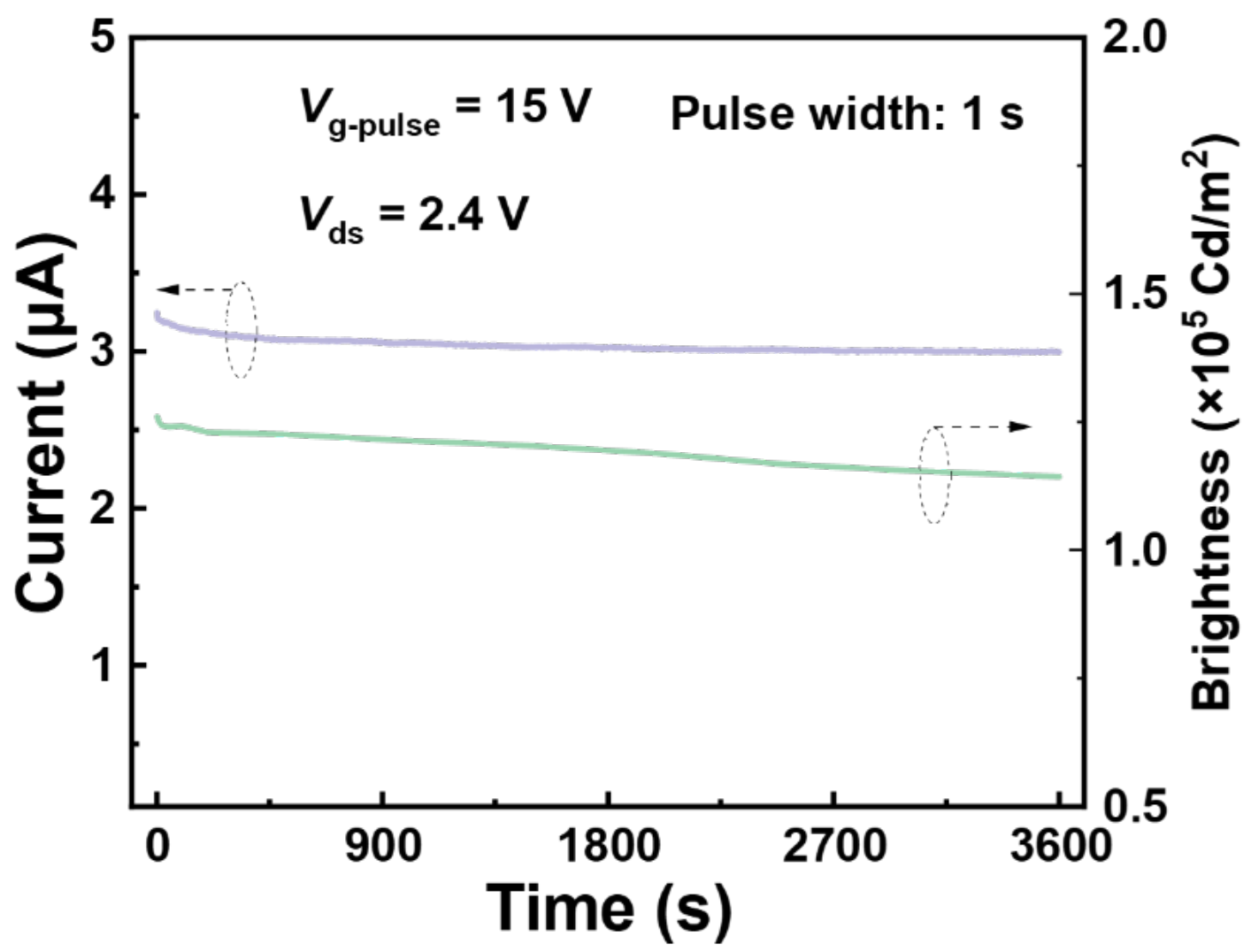

Figure R22: Retention performance of integrated devices. The variation of current and brightness with a reading voltage of 2.4 V upon a program operation ($V_{g\text{-}pulse}$ = 15 V, with width of 1 s).

3. Statistical support for device uniformity

The authors claim excellent uniformity; however, device-to-device and chip-to-chip statistical analyses are limited. In Figure 2g, the on/off ratio varies by up to a factor of ~4, yet the acceptability of this variation for display operation is not discussed. Providing I–V characteristics for all measured devices and, if available, chip-to-chip or position-dependent data across the 4-inch wafer would better support the scalability and uniformity claims.

Response: We thank the reviewer for this valuable comment regarding device uniformity and statistical analysis. The results were provided in revised Supplementary Figure xx.

To better support the scalability and uniformity of the proposed system, we have supplemented comprehensive statistical measurements across the 4-inch wafer. Specifically, ten devices were randomly selected from each of nine shots (Figure R23a), yielding a total of 90 devices. The transfer characteristics in both type-I and type-II modes are summarized in Figure R23b–R23c, showing consistent switching behavior with a high yield of 97.8%.

Statistical analysis (Figure R23d–R23e) indicates that the average on/off ratios are 1.63×10^8 (type-I) and 1.71×10^7 (type-II), with threshold voltage (V_{th}) variations confined within ~1.5 V across all devices. This level of uniformity is comparable to or better than previously reported works (Nano Lett. 2023, 23, 9333−9339, Nature Electronics, 2023, 6(12): 991-998, Nature Electronics, 2024, 7(10): 868-875).

Regarding the variation of the on/off ratio (up to a factor of ~4), we note that this does not

compromise display performance. The on/off ratios remain sufficiently high to ensure clear optical contrast, and the Micro-LED brightness is primarily governed by the programmed current level. Furthermore, spatial mapping of the programmed current across the wafer shows that the maximum-to-minimum current ratio is less than 2. For devices with slightly higher current, reducing the programming voltage (e.g., to 13 V) can further effectively improve uniformity, decreasing the standard deviation.

These results demonstrate that the observed variation is both acceptable for display operation and tunable through programming conditions. In addition, the dual-mode operation provides further flexibility, where type-II mode can be used for pixel-level adjustment if needed.

Overall, the statistical analysis and wafer-scale mapping confirm the excellent uniformity, high yield, and scalability of the proposed platform for large-area integrated display–illumination applications. We have also added discussion in the revised manuscript (page 6, line 178). And detailed uniformity performance would be presented in the Supporting Figure 15 (page 18, line 216) and Supplementary Figure 16 (page 18, line 224).

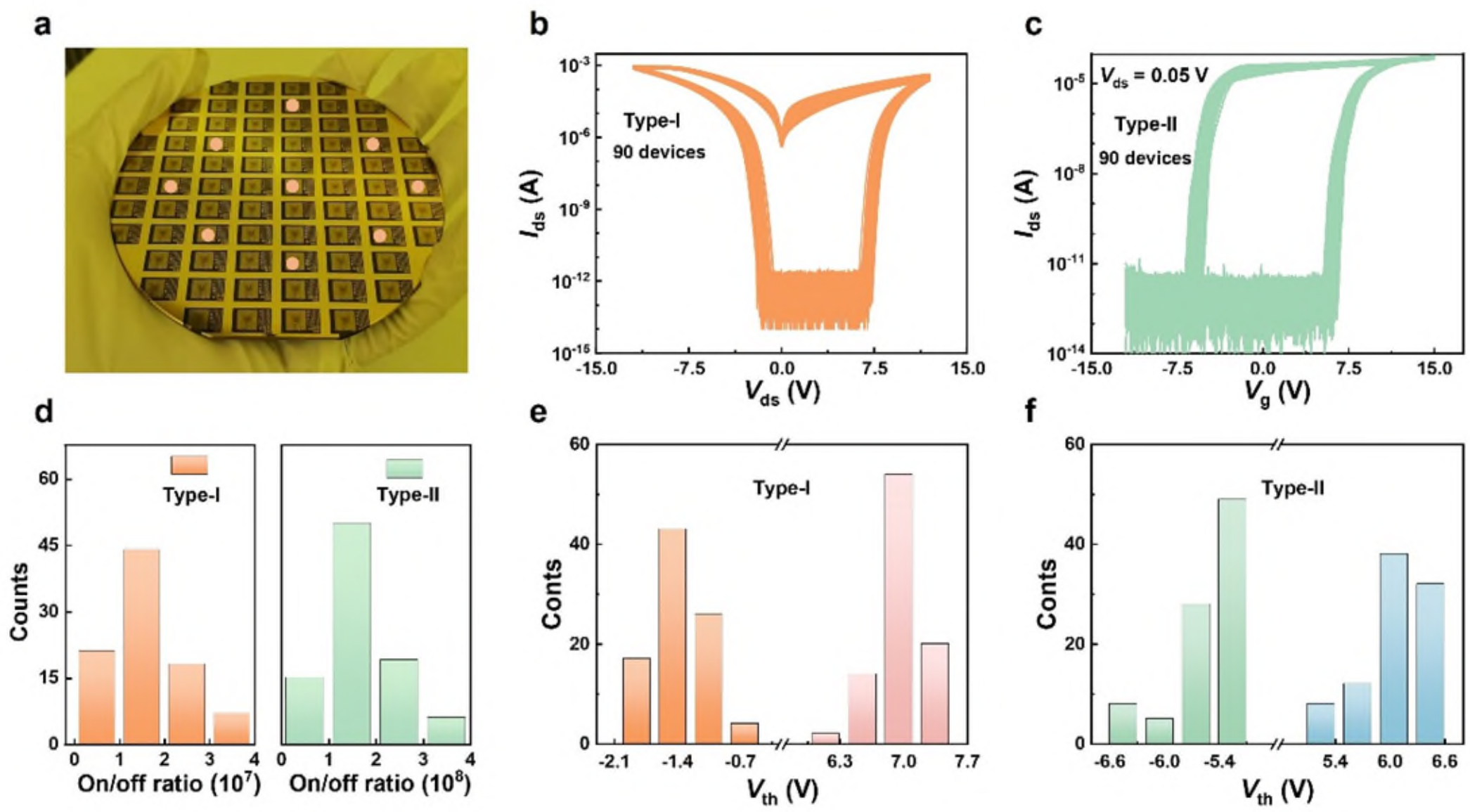

Figure R23: a, Schematic diagram of point selection on a 4-inch wafer. **b,c,** Summary of transfer characteristic curves obtained from 90 devices with type-I mode (a) and type-II mode (b) respectively. d, Statistical of current on/off ratios under two modes. **e,f,** Statistical of positive and negative threshold voltage under two modes with type-I mode (e) and type-II mode (f) respectively.

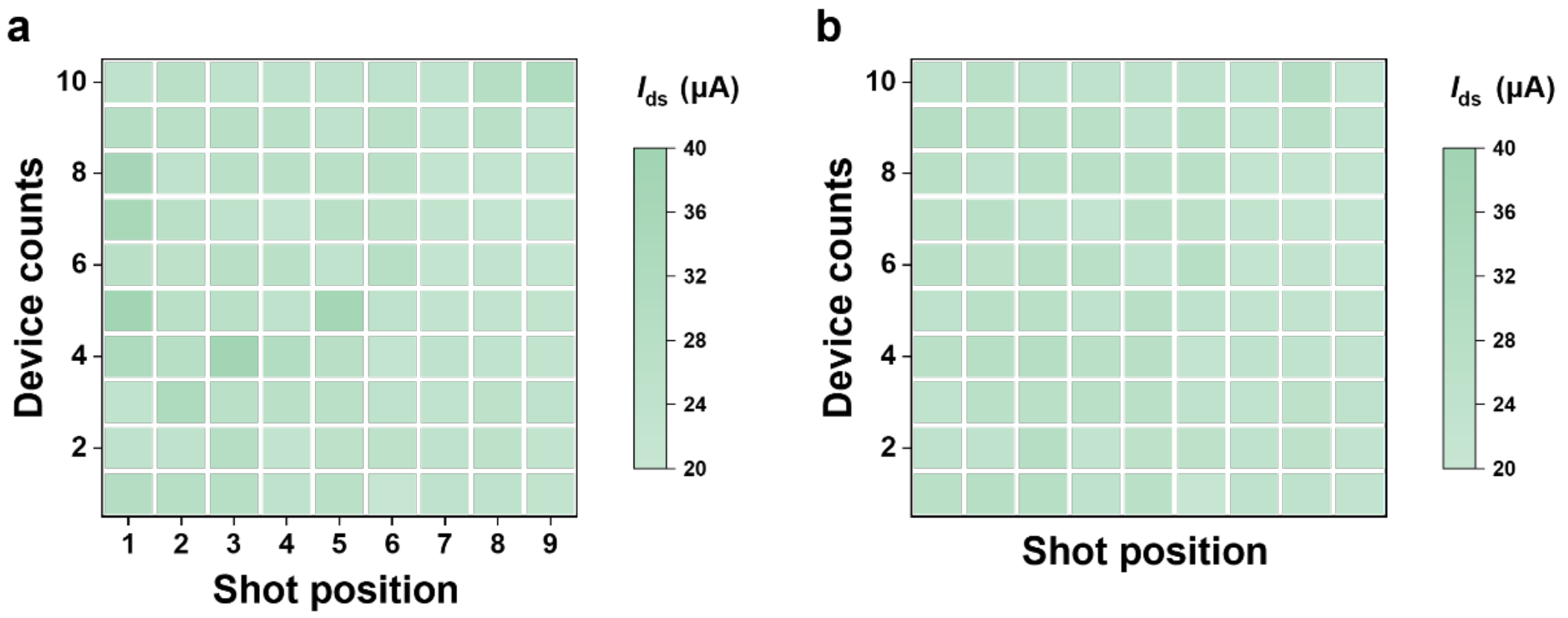

Figure R24: a, Current distribution mapping of different devices upon program operation (+15 V, 0.1 s). **b,** Spatial current distribution of high-current devices after a decrease in the programming voltage (+13 V, 0.1 s).

4. Comparison with existing FGM-based devices

The energy and performance comparisons focus primarily on TFT-based drivers, which limits evaluation of the proposed FGM architecture itself. Even in the supplementary comparison among FGM-based devices, the presented metrics do not clearly demonstrate a distinct performance advantage of the proposed device. As improved retention enabled by the FGM is a central claim of this work, the lack of clear superiority over previously reported FGM-based drivers weakens the case for novelty. A more critical and quantitative comparison with existing FGM-based devices, based on key metrics such as switching energy, retention, leakage current, and program/erase conditions, is necessary to convincingly establish the relative merits and technological significance of the proposed approach.

Response: We thank the reviewer for this insightful comment regarding the comparison with existing FGM-based devices.

First, we would like to clarify that the primary comparison with TFT-based drivers is motivated by the **target application in Micro-LED display systems**, where TFTs represent the current mainstream driving technology. In this context, our FGM-based architecture enables **nonvolatile operation without continuous bias**, offering a fundamental advantage in reducing power consumption, which is not achievable in conventional TFT-based drivers.

At the system level, the energy consumption differs fundamentally: TFT-based drivers require a continuous bias to maintain the display state, whereas FGM-based drivers rely only on pulse programming and consume virtually no static power during retention. Based on experimental measurements, the switching energy of our FGM-integrated pixel is as low as 11.41 pJ, while

the total energy consumption for maintaining illumination over 5 minutes is significantly lower than that of TFT-based drivers (e.g., ~10^3–10^5 pJ for reported TFT technologies), demonstrating a clear advantage for low-refresh-rate or static display scenarios.

To address the reviewer's concern, we have further systematically compared the intrinsic performance of our FGM device with previously reported FGM-based devices (Table R2) in terms of key metrics:

(1) **Switching energy and speed:** Our device achieves a minimum switching time of 10 μs, corresponding to a switching energy of ~1.14 fJ per event, and even decreased to 0.372 fJ (the FGMs with 10-nm Al_2O_3 tunneling layer).This performance is comparable to prototype floating-gate memory devices based on mechanically exfoliated two-dimensional materials (e.g., MoS_2 FGMs with sub-fJ switching energy but limited scalability, Nature Nanotechnology, 2022, 17, 500–506), and is lower than that of typical large-area metal-oxide FGMs, which generally exhibit switching energies in the fJ–pJ range (Adv. Mater., 2024, 36, 2308502), while maintaining full compatibility with wafer-scale fabrication.

(2) **Retention performance:** Long-term retention exceeding 6000 s is demonstrated in both type-I and type-II modes, with stable programmed states and high on/off ratios (>10^7). In comparison, previously reported large-area FGMs typically exhibit retention in the range of 10^3–10^4 s with on/off ratios of 10^5–10^7 (Nat. Commun., 2016, 7, 12725; Adv. Mater., 2017, 29, 1703363; Adv. Funct. Mater., 2016, 26, 5701–5707; Adv. Mater. 36, 2308502 (2024)). Our device therefore achieves comparable or improved retention performance while additionally supporting dual-mode operation.

(3) **Leakage current:** A low leakage current of ~7.6 pA is achieved, which is close to the measurement limit of our instrumentation, and approaches the best reported levels in advanced FGM devices (e.g., ~4 pA in optimized oxide-based FGMs, Adv. Mater., 2024, 36, 2308502), while being significantly lower than typical values (10–100 pA) reported in two-dimensional or oxide semiconductor FGMs. This ultra-low leakage current is critical for enabling stable and low-power nonvolatile operation.

(4) **Program/erase conditions:** The device supports dual-port programming, with type-I mode enabling drain-driven global programming (~12 V) and type-II mode enabling gate-controlled selective programming (~15 V), while pulse widths can be reduced to as short as 10 μs. In comparison, previously reported FGMs typically require programming voltages in the range of ~10–20 V with pulse widths spanning from microseconds to milliseconds depending on material systems (Adv. Mater., 2024, 36, 2308502; Nat. Commun., 2016, 7, 12725). Our device provides comparable programming conditions while introducing enhanced operational flexibility through dual-mode control.

Importantly, while individual metrics are comparable to state-of-the-art FGM devices, the key

novelty of this work lies in the device–circuit co-design: A single FGM per pixel, combined with dual-mode operation, enables both nonvolatile image retention and dynamic pattern switching, which is not achievable in previously reported FGM-based drivers. This architecture also allows the system to operate with zero static power consumption during retention, while maintaining compatibility with large-area fabrication, thereby providing a unique solution for energy-efficient Micro-LED display–illumination systems.

In the revised Supplementary information (page 32), Table S1 has been updated to better highlight the superiority of our developed FGMs device.

Tabel R2 Summary of recent advances in various floating-gate memory devices.

Device structure	P/E Voltage (V)	Reading Voltage (V)	Reading current (A)	On/off ratio	Leakage current (pA)	Switching Energy (fJ)	Retention	Endurance (cycles)	Large-scale fabrication	Ref
MoS_2 / h-BN /Gra	+8 / -8	0.5	3.9×10^{-6}	1×10^{8}	/	14.4	>1000 s	/	no	10
MoS_2 / h-BN / Gra	+35 / -35	0.05	5×10^{-6}	$\approx 5 \times 10^{6}$	~4	0.0028	5300 s	1390	no	11
MoS_2 / h-BN / Te	+15 / -15	0.1	0.1 μA/μm	$\approx 10^{8}$	$>10^{-6}$ μA/μm	0.075	4000 s	4000	no	12
MoS_2 / h-BN / MLG	+25 / -25	0.05	$\approx 3 \times 10^{-6}$	1×10^{8}	> 100	0.0604	10^{4} s	4200	no	13
MoS_2 / h-BN / Gra	+20 / -20	0.1	$\approx 5 \times 10^{-6}$	$\approx 10^{5}$	48.2	771.2	>1500 s	1000	no	14
BP / Al_2O_3 / BP	+10 / -10	-0.05	$\approx 3 \times 10^{-6}$	$\approx 10^{6}$	≈ 20	~ 2	>100 s	5000	no	15
DPPDTT / Al_2O_3 / Au NPs	+100 / -100	60	$\approx 10^{-3}$	$\approx 10^{3}$	$\approx 10^{5}$	10^{8}	/	/	Yes (solution)	16
InSe / h-BN / MLG	+30 / −30	-1	$\approx 10^{-5}$	$\approx 10^{8}$	> 100	> 0.06	3000 s	860	no	17
ZnO / Al_2O_3 / ZnO	+15 / -15	0.1	$\approx 10^{-6}$	$\approx 10^{7}$	~ 6	~9	10^{4} s	10^{4}	Yes (ALD)	18
IGZO / Al_2O_3 / ZnO	+20 / -20	0.1	$\approx 10^{-6}$	$\approx 10^{5}$	6.62	3.972	1800 s	/	Yes (ALD)	19
ITO / Al_2O_3 / ITO	+15 /-12	0.05	4.3×10^{-5}	1.8×10^{8}	7.61	1.14	6000 s	>4000	Yes (sputter)	This work
ITO / Al_2O_3 / ITO	+8 /-8	0.05	2.43×10^{-5}	1.2×10^{8}	3.84	0.372	6000 s	>8000	Yes (sputter)	This work

5. Use of the term “multi-modal”

The term “multi-modal” is typically used to describe systems that operate across distinct modalities or sensory domains. In the present manuscript, the term is used to denote the coexistence of lighting and display functions, which both belong to the optical domain. As such, the use of “multi-modal” may be conceptually inaccurate. The authors should reconsider this terminology or clearly justify its usage in the context of the proposed device.

Response: We thank the reviewer for this insightful comment. We agree that, in a broad context, the term “multi-modal” typically refers to systems operating across distinct sensory modalities, and its usage here may lead to ambiguity.

In our work, the intention was to describe the integration of two distinct operational functionalities—display and illumination—within a single device platform, which are traditionally implemented using separate systems. To improve conceptual clarity and avoid potential misunderstanding, we will replace “multi-modal” with “dual-function” throughout the revised manuscript. We believe this revision more accurately reflects the nature of the proposed system and addresses the reviewer’s concern.

In the revised manuscript, the title has been replaced to “Dual-function Floating-Gate Memory Driver for Energy-Efficient Integrated Display–Illumination System”.

Minor comments

1. The operating principle illustrated “on the right side of Figure 1a” (lines 97–98) requires clearer explanation.

Response: Thank you for your comment. We apologize for the confusion caused by the text. The phrase “The operating principle of the integrated driving circuit is illustrated on the right side of Figure 1a” was a typographical error. The right side of Figure 1a actually shows the detailed device structure, while the operating mechanism is illustrated in Supplementary Figure S3. We have corrected this in the revised manuscript for clarity.
In the revised manuscript (page 4, line 97), the sentence is revised to “The detailed device structure of the integrated driving circuit is illustrated on the right side of Figure 1a”.

2. The pulse width used for retention measurements in Figure 2e should be specified.

Response: Thank you for pointing out the missing parameter for retention measurement, the pulse width of 1 s was employed, and we have added it in the revised manuscript.

In the revised manuscript (page 18, line 481), the context of "Pulse width: 1 s." has been added.

3. Figure 3g should include a legend.

Thank you for pointing out the mistake in the Figure 3g.

We have added a legend to Figure 3g in the revised manuscript to clearly indicate the output characteristics of FGM device with different gate pulse voltage.

4. The descriptions of Fig. 4b and Fig. 4c (line 262) appear to be interchanged.

Response: Thank you for pointing out the typo in Line 262.

We have corrected the figure references in line 276 and Line 280so that the descriptions of Fig. 4b and Fig. 4c now correctly correspond to the figures in the revised manuscript.

5. The term "IO layer" in the Materials and Methods section (line 298) should be corrected or defined.

Response: Thank you for pointing this out. In the original text, "IO layer" is a typo. It should be corrected to "ITO layer," referring to the 110-nm-thick indium tin oxide used to form an ohmic contact with the p-GaN layer, ensuring proper device operation.

In the revised manuscript (page 11, line 313), we have corrected the sentence of "Then 110-nm thickness ITO layer was deposited".

6. The origin and role of the ITO layer observed in Supplementary Figure 1a TEM image should be clarified.

Response: Thanks for your insightful comment. The 110-nm-thick ITO layer, as observed in Supplementary Figure 1a, was deposited on the epi-wafer via sputtering and subsequently annealed at 500 °C for 3 minutes. This process facilitates a high-quality ohmic contact with the p-GaN layer, which is essential for efficient carrier injection and stable device operation (Light: Science & Applications, 2024, 13:284).

We have clarified this in the Materials and Methods section (in the revised manuscript page 11, line 320) and in the caption of Supplementary Figure 1 (page 4, line 59).

Reference:

1. Wang, F. et al. Micro-LED/van der Waals heterointegration for in-pixel processing display architecture. Nat. Commun. 17 (2026).
2. Yuk, S. H. et al. Transparent Active-Matrix Micro-LED Array Driven by Vertically Stacked TiO2/HfO2-Based Memristors. *Adv. Mater. Technol.* **11**, e01709 (20226).
3. Lee, H. J. et al. Monolithic integration of sub-volt memristor-driven pixels for capacitorless active-matrix micro-LED displays. *Int. J. Extrem. Manuf.* **8**, 025501 (2026).
4. Huang, T. et al. Flexible active-matrix micro-LED display with 1T-1FeMFET architecture featuring scaling-limit-free design. Nat. Commun. (2026).
5. Jin, T. et al. Demonstration of programmable light intensity of a micro-LED with a Hf-based ferroelectric ITZO TFT for Mura-free displays. *Nanoscale Adv.* **5**, 1316-1322 (2023).
6. Xu, M. et al. Nonvolatile Memory Organic Light-Emitting Transistors. *Adv. Mater.* **35**, 2307701-2307703 (2023).
7. Hwangbo, S., Hu, L., Hoang, A. T., Choi, J. Y. & Ahn, J.-H. Wafer-scale monolithic integration of full-colour micro-LED display using MoS2 transistor. *Nat. Nanotechnol*. **17**, 500-506 (2022).
8. Hwang, K. et al. 1.6-Inch Transparent Micro-Display with Pixel Circuit Integrated microLED Chip Array by Misalignment-Free Transfer. *Adv. Mater*. **37**, 2416015 (2025).
9. Wang, Y. et al. Atomically Thin ITO Transistor Matrix for Monolithic Integration of Ultrabright Micro-LED Displays. *Nano Lett*. **25**, 18091-18099 (2025).
10. Sun, X. et al. Reconfigurable logic-in-memory architectures based on a two-dimensional van der Waals heterostructure device. *Nat. Electron.* **5**, 752-760 (2022).
11. Liu, L. et al. Ultrafast non-volatile flash memory based on van der Waals heterostructures. *Nat. Nanotechnol*. **16**, 874-881 (2021).
12. Zha, J. et al. A 2D heterostructure-based multifunctional floating gate memory device for multimodal reservoir computing. *Adv. Mater.* **36**, 2308502 (2024).
13. Wang, H. et al. Ultrafast non-volatile floating-gate memory based on all-2D materials. *Adv. Mater*. **36**, 2311652 (2024).
14. Li, W. et al. Demonstration of nonvolatile storage and synaptic functions in all-two-dimensional floating-gate transistors based on MoS2 channels. *ACS Appl. Electron. Mater*.**5**, 4354-4362 (2023).
15. Xiong, X., Wang, X., Hu, Q., Li, X. & Wu, Y. Flexible synaptic floating gate devices with dual electrical modulation based on ambipolar black phosphorus. *Iscience* **25** (2022).
16. Chen, Y. et al. Nanofloating gate modulated synaptic organic light-emitting transistors for reconfigurable displays. *Sci. Adv.* **8**, eabq4824 (2022).
17. Wang, H. et al. Ultrafast-Programmable 2D Homojunctions Based on van der Waals Heterostructures on a Silicon Substrate. Adv. Mater. 35, 2301067 (2023).
18. Zhang, N. et al. Transparent floating gate memory based on ZnO thin film transistor with

controllable memory window. *IEEE Journal of the Electron Devices Society* **10**, 275-280 (2022).

19. Ryoo, H.-J. & Yoon, S.-M. Channel conductance modulation of dual-gate charge-trap oxide synapse TFT using In-Ga-Zn-O channel and ZnO trap layers. *IEEE Electron Device Lett.* **41**, 1661-1664 (2020).

Reviewer #1 (Remarks to the Author):

The authors have made substantial and satisfactory revisions to address the concerns raised during the review process. The manuscript now meets the high standards of Nature Communications in terms of technical quality and clarity of presentation. Therefore, I recommend that the revised manuscript be accepted for publication in its current form.

Response: We sincerely thank the reviewer for the positive recognition of our revisions to the manuscript and for the recommendation for publication.

Reviewer #2 (Remarks to the Author):

This manuscript has been improved according to the reviewers' comments. However, minor revision is required for publication. The main comments are as follows:

1. A whole experimental data should be clearly described in Figure R12b.

Response: We sincerely thank the reviewer for this constructive suggestion. Complete data can better demonstrate the reliability of the floating gate memory (FGM) during cyclic measurement. Meanwhile, additional detailed discussion has been provided as below:

Endurance performance is demonstrated through repeated program/erase cycles in Type-I mode (Figure R1b, original Figure R12b), showcasing a stable on/off current ratio about 10^7 without obviously degradation during 4000 cycles endurance operation. Furthermore, the variation of on-state current was only ±1.41 μA, which posed virtually no visual impact on Micro-LED lighting and display applications, exhibiting excellent stability and repeatability.

In the revised Supplementary Information, the corresponding data figures have been updated in Supplementary Figure 10.

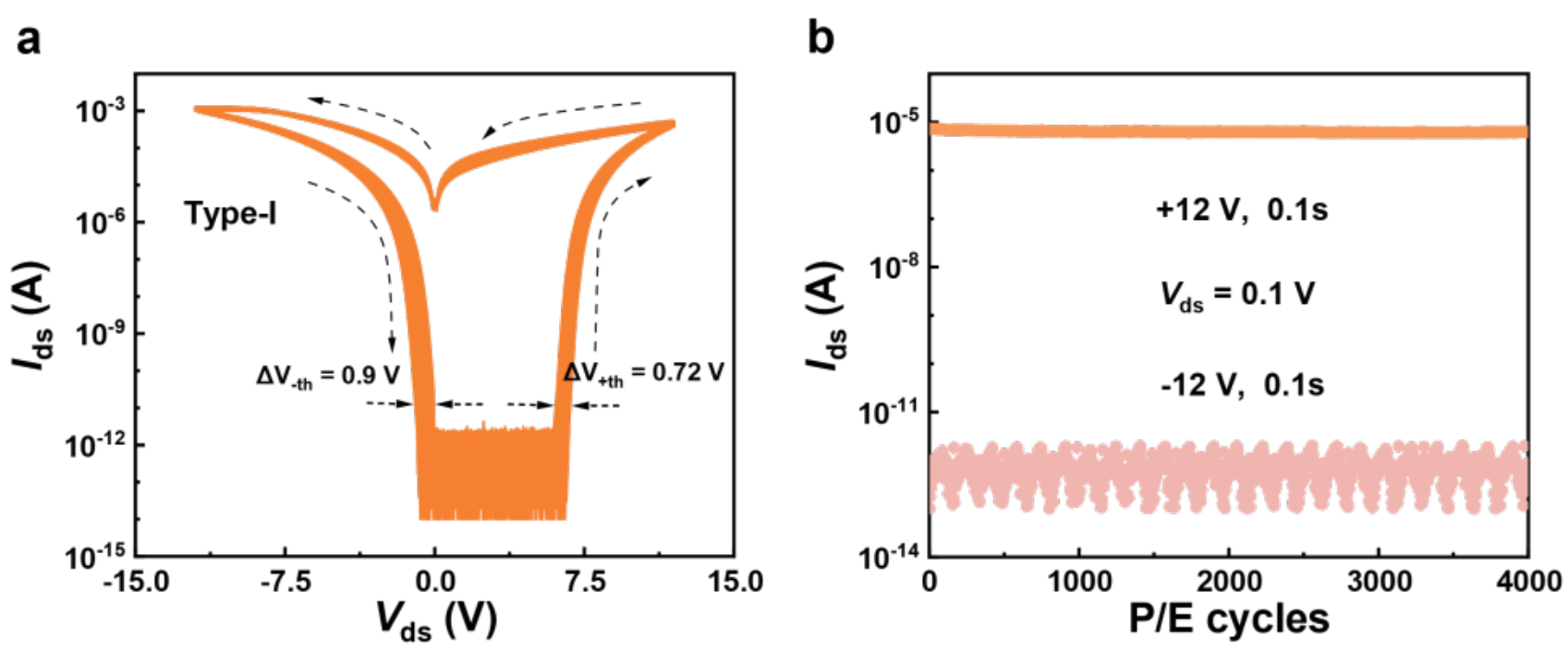

Figure R1 (Original Figure R12): Electrical stability and endurance of the FGM device in type-I mode. a, Transfer characteristics of the same FGM device measured continuously over 200 cycles. **b,** Endurance performance under alternating voltage pulses (V_g = +12 V/–12 V, pulse width = 0.1 s), with I_{ds} read at V_{ds} = 0.05 V and V_g = 0 V.

Reviewer #3 (Remarks to the Author):

I appreciate the authors' efforts in revising the manuscript and providing additional explanations, figures, and supporting data. The added energy-band diagrams, device-uniformity data, and revised terminology significantly improve the clarity of the manuscript. However, there are still several critical points regarding the physical mechanism and fair metric comparisons that must be addressed before publication.

Response: We are pleased that our responses have overall addressed the reviewer's main concerns and sincerely appreciate the reviewer's recognition of our work. For the remaining points, we have made the corresponding revisions as detailed below:

[Major Comments]

1. While the revised explanations and energy-band diagrams help clarify the intended Type-I and Type-II operations, the Type-I programming mechanism under a drain-only bias with a floating gate remains non-intuitive, particularly given the relatively thick 18 nm Al_2O_3 tunneling layer. The authors cite previous two-terminal memory demonstrations (Nat. Commun. 2016, 7, 12725; Adv. Mater. 2017, 29, 1703363) which utilized highly conductive graphene floating electrodes to effectively establish an electric field across the tunneling dielectric. In the present work, however, both the top ITO channel and the bottom floating ITO layer appear to be fabricated via the same process, implying similar conductivity. Because the off-state current of the ITO channel is extremely low, the channel is nearly insulating in this state. Consequently, it is not immediately clear how the drain bias generates a sufficiently strong vertical electric field across the 18 nm Al_2O_3 layer, rather than predominantly dropping laterally along the top ITO channel. Please

Response: We sincerely thank the reviewer for this professional and insightful comment regarding the Type-I programming mechanism modulated with drain electrode.

As shown in Figure R2, the charge-trap layer of ITO was deposited first via sputter, and patterned by photolithography and wet etching ($FeCl_3$/HCl solution). Next, an 18-nm Al_2O_3 dielectric layer was deposited by atomic layer deposition (ALD) under vacuum conditions at 250 °C. Then a 6-nm semiconductor material (ITO) onto the charge-trap layer by sputtering. Although the top ITO channel and the bottom floating ITO layer were fabricated via the same process. However, the properties of ITO will transition from semiconducting to metallic upon the deposition of the Al_2O_3 dielectric layer via ALD under vacuum and high-temperature conditions. This is similar to an annealing treatment onto ITO, causing oxygen atoms escaped from the lattice and form oxygen vacancies, thereby greatly increasing the electrical conductivity of the ITO material.

To demonstrate the change in electrical conductivity of ITO before and after Al_2O_3 deposition

by ALD, transfer characteristic curves were conducted with the ITO-based TFT devices (Figure R3a). The results exhibit a superior on/off ratio (over 10^8) and excellent gate controllability (Figure R3c), confirming the semiconducting property of the ITO material. Subsequently, an 18-nm Al_2O_3 layer was performed on the device under the same conditions as described in the main text, followed by electrode etching to open contact windows (Figure R3b). The transfer characteristic curves show that the device loses its gate control capability, with the current remaining largely unchanged with varying gate voltage, indicating metallic behavior (Figure R3d).

Upon Al_2O_3 deposition via ALD, the ITO floating-gate layer in this work exhibits metallic properties, enabling the formation of a sufficient vertical electric field modulated by the drain electrode in the two-terminal memory mode, similar to that reported in the literature (Nat. Commun. 2016, 7, 12725; Adv. Mater. 2017, 29, 1703363), which have been cited as Ref- 43/44 to provide theoretical support in the manuscript. To this end, a Pt-based FGM device was also fabricated, with a structure consistent with that of the ITO-based FGM device described in the manuscript (Figure R4a), and optical micrograph is shown in the Figure R4b. The I-V curves of the Pt/ITO-based FGM devices were measured and compared, demonstrating similar memory characteristics (Figure R4c), which experimentally confirm that our developed ITO/Al_2O_3/ITO FGM devices can realize two-terminal memory functionality.

Aid to facilitate readers' understanding, we have indicated the changes in the conductive state of the ITO floating-gate layer during the fabrication process in revised manuscript (Page 4, Line 108; Page 4, Line 130) and Supplementary Figure 2.

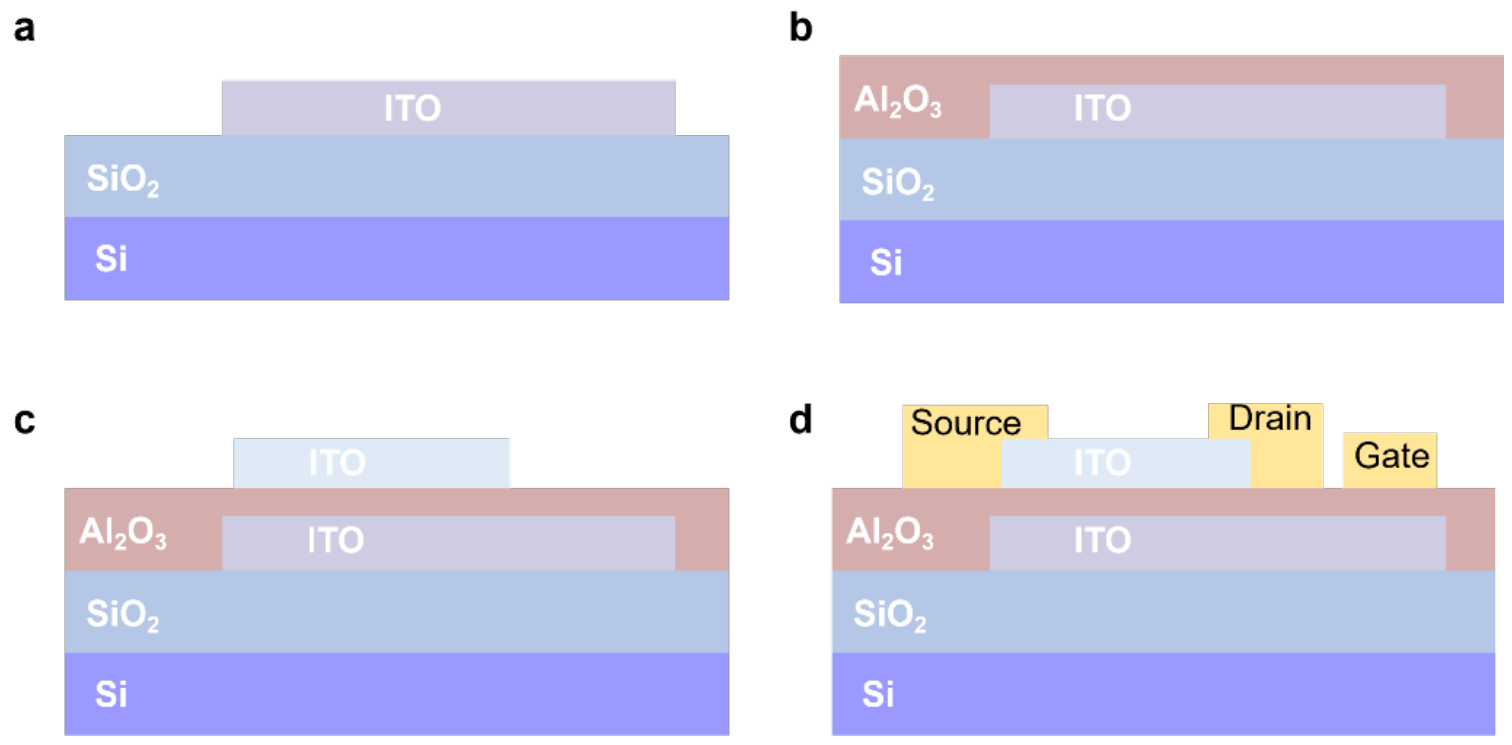

Figure R2: process flow diagram of floating-gate memory fabrication. a, Deposition of ~6-nm ITO as the charge-trap layer by sputtering, patterned by photolithography and wet etching ($FeCl_3$/HCl solution). **b,** Deposition of ~18-nm Al_2O_3 dielectric layer by atomic layer deposition (ALD). **c,** Vertical stacking of 6-nm semiconductor material onto the charge-trap layer by sputtering. **d,** Deposition of D/S/G electrodes layers of 5 nm/50 nm Cr/Au by electron beam evaporation (EBE).

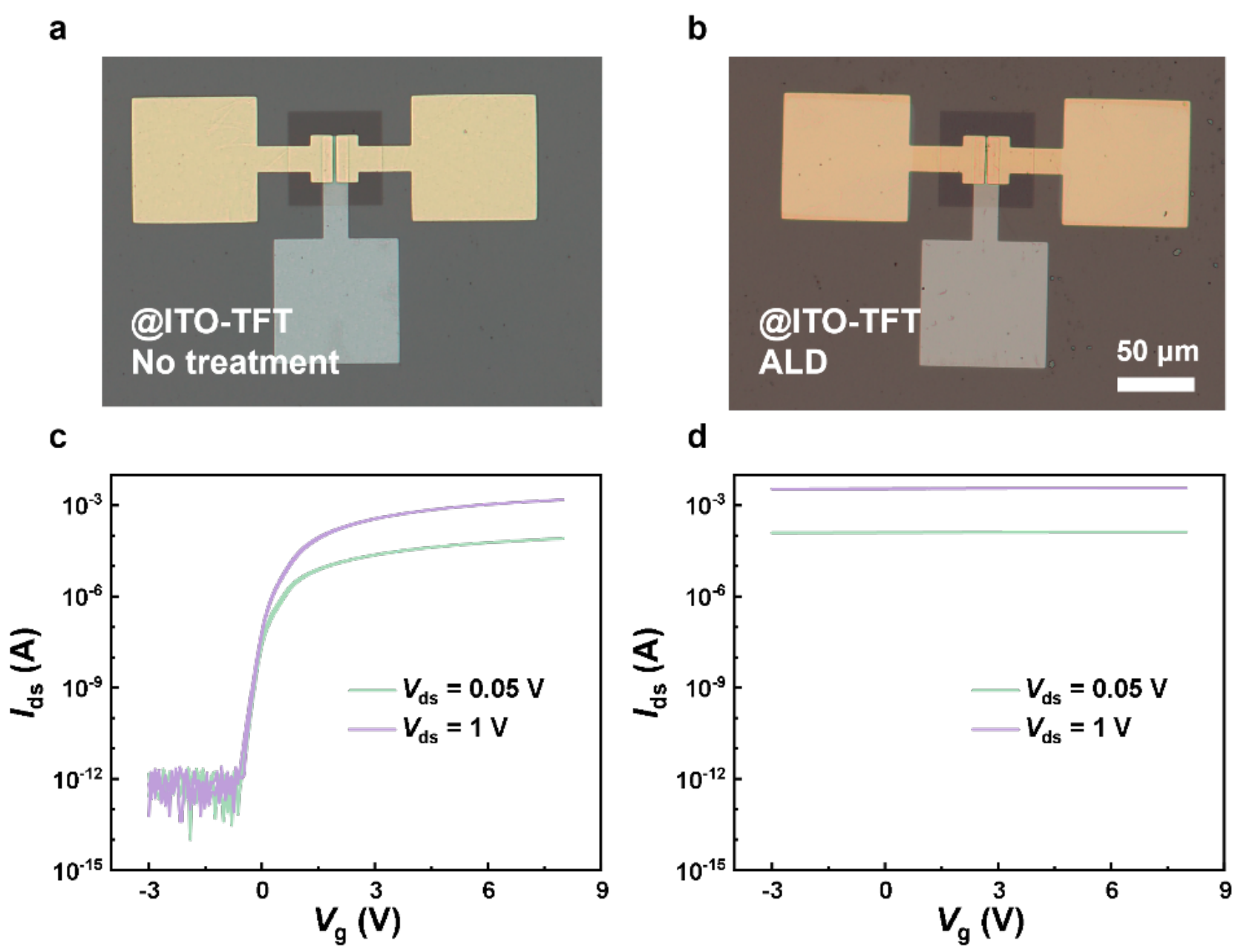

Figure R3: Comparison of ITO-based thin-film transistors with and without ALD treatment. a,b, Optical micrographs without (a) and with ALD treatment (b). **c,d,** Transfer performance without (c) and with ALD treatment (d).

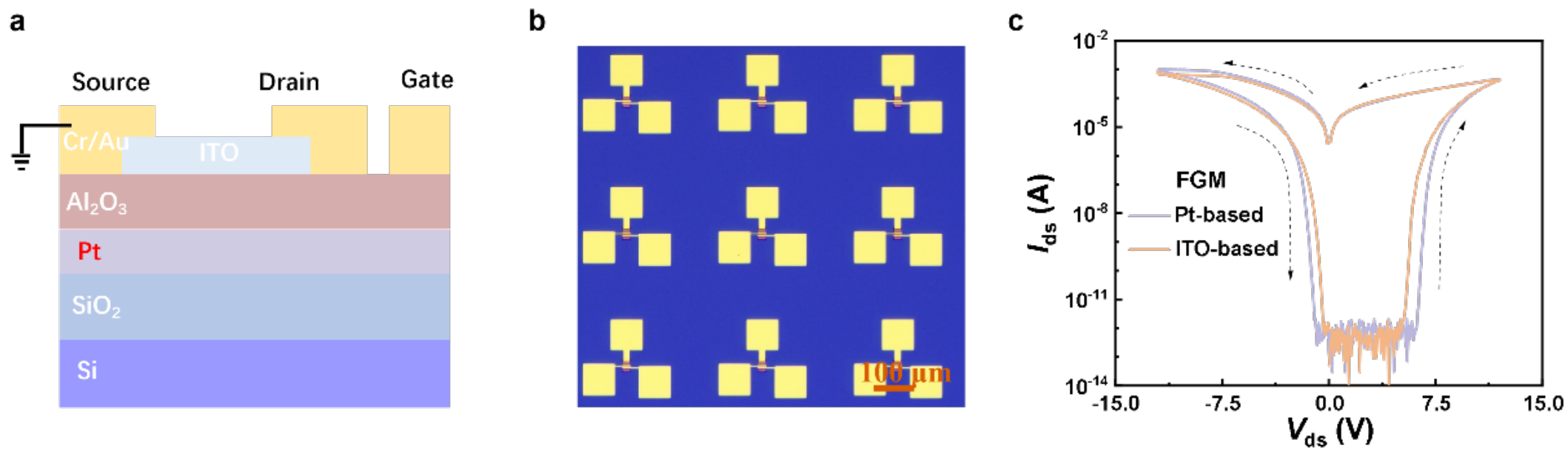

Figure R4: Structure and electrical properties of Pt-based floating-gate memory. a, Schematic diagram of the floating-gate memory, consistent with the architecture in the main text. **b,** Optical micrographs of the Pt-based FGMs. **c,** The I-V curves of Pt/ITO- based FGMs.

a) Please explicitly confirm whether the underlying body/substrate/LED stack is electrically grounded or floating during the Type-I measurement, as this strongly influences the field distribution.

Response: Thanks for your professional comment regarding the type-I operation mechanism.

As shown in Figure R5, in type-I mode, sweep transfer characteristics of the FGM device were conducted controlled by drain pulse voltage from -12 V to +12 V under underlying body (Si) floating and grounded, respectively, showcasing no obviously distinction, and indicating program and erase are achieved via a drain–source bias applied to the FGM device, while the

underlying body is electrically floating. The floating gate (conductive ITO layer) forms a capacitive coupling with both the drain and source, allowing a sufficient local electric field to be established across the Al_2O_3 tunneling dielectric. This field enables holes to tunnel into the charge trapping layer, primarily via Fowler-Nordheim (F-N) tunneling.

Furthermore, we have compared the two-terminal transfer characteristics of the FGMs on different substrates, and found that the memory curves were similar. The FGMs on the Micro-LED wafer were fabricated on an ultra-flat SiO_2 surface after chemical mechanical polishing (CMP), with a SiO_2 thickness of approximately 1.7 µm. It would that ensure the FGM were electrically isolated from the underlying metal, having no capacitive coupling effect between the ITO floating gate layer and the bottom substrate. This further demonstrates that a vertical electric field was formed between the drain and the floating gate layer under Type-I mode, enabling charge injection from the drain into the floating gate, thereby modulating the channel carriers. Meanwhile, the Micro-LED integrated ITO-based FGM yielded memory characteristics within the positive voltage range (the Micro-LED exhibits reverse-broken characteristics) through applying pulse voltages to the anode, further confirming that the charges stored in the floating gate were injected from the drain and were independent electrically with the bottom substrate.

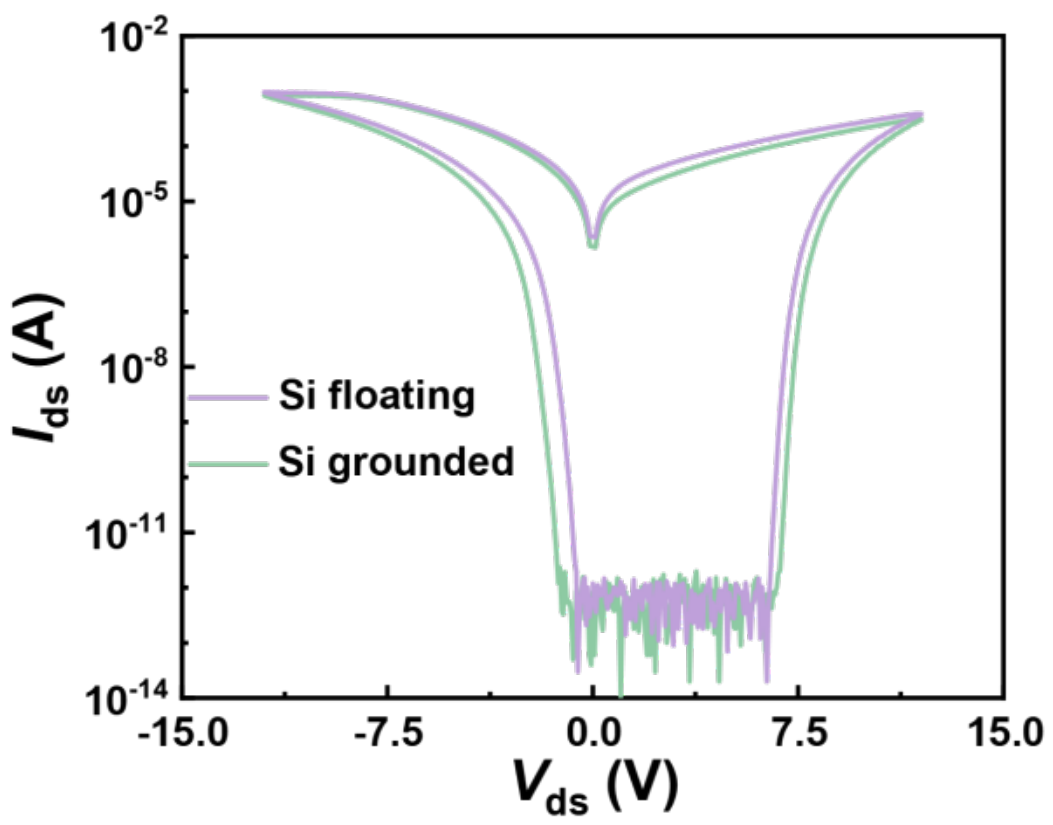

Figure R5: Sweep transfer characteristics of the FGM device controlled by drain pulse voltage from -12 V to +12 V with type-I modulation under gate floating and grounded.

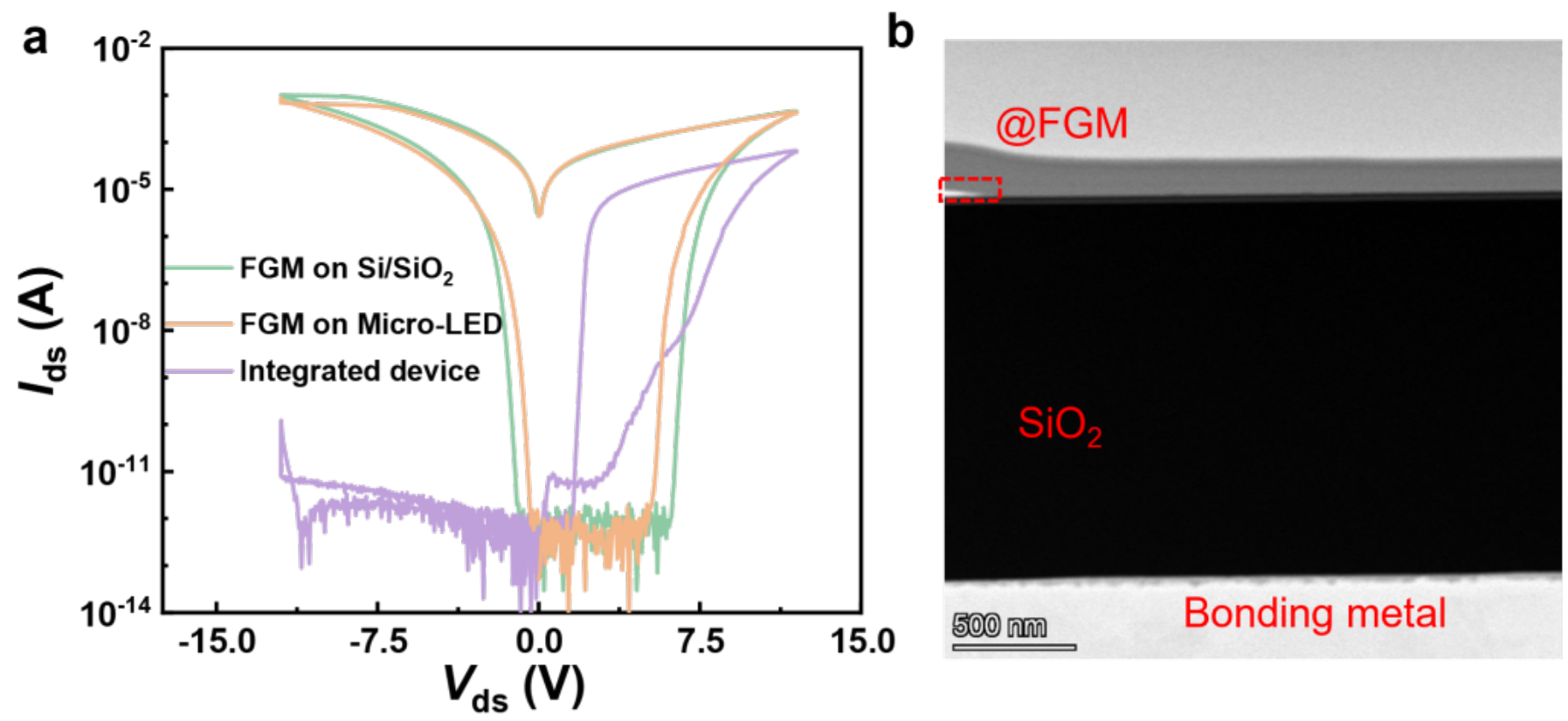

Figure R6: a, Sweep transfer characteristics of the FGM on Si/SiO_2, on Micro-LED and the integrated device, measured with drain pulse voltage from -12 V to +12 V under type-I modulation. **a,** Cross-sectional TEM image of FGM integrated on a Micro-LED wafer.

b) Please provide robust evidence demonstrating that a sufficient vertical field is established across the Al_2O_3 layer. This can be achieved by providing: An electrostatic simulation under actual Type-I bias conditions using realistic material parameters such as the specific conductivity of the ITO layers; OR Thickness-dependent tunneling-current measurements for the Al_2O_3 oxide similar to the cited Nat. Commun. work; OR another clear experimental or theoretical demonstration.

Response: We appreciate the reviewer's insightful question regarding the establishment of a sufficient vertical field across the Al_2O_3 layer under Type-I bias conditions. Below we provide robust evidence from both electrostatic simulation and experimental characterization to address this concern. COMSOL Multiphysics 6.2 was employed to solve the Poisson equation for electrostatic potential: ∇·(ε_r·∇V) = 0. The simulations were conducted under actual Type-I bias conditions, specifically program (+12 V) and erase (−12 V) states, with the complete device geometry.

Detailed electrostatic simulation parameters are provided in the supplementary information.

A sufficient vertical field established across Al_2O_3 is directly validated with two key findings from our simulations: Figure R7a and R7b depict the simulated electrostatic potential distribution of FGM at the states of program and erase, and referring to Table R1, a significant potential difference is observed between the drain and the floating gate layer. Under pulse voltages of +12 V and –12 V, the average potential difference reaches as high as 7.25 V. Although the average electric field across the 18 nm Al_2O_3 layer calculated from the capacitive divider model is 4.03 MV·cm^{-1} at $V_{d\text{-}pulse}$ = 12 V (Table R1), our 2D electrostatic simulation reveals significant field crowding at the drain electrode edge (width = 10 nm, occupying only

20 % of the total lateral dimension). This geometric field enhancement yields localized E-$_{peak}$ values of 6.12 MV·cm^{-1} within a narrow region (approximately 2–5 nm) directly beneath the drain edge (Figure R8). This peak field exceeds the well-established F-N tunneling threshold for Al_2O_3. According to previous systematic studies (J. Mater. Sci.: Mater. Electron. 2018, 29, 15496–15501.), F-N tunneling in ultra-thin (6 nm) Al_2O_3 becomes dominant when E > 5 MV·cm^{-1}. Our simulated peak fields therefore meet and surpass this criterion locally, enabling non-uniform yet physically plausible charge injection.

In fact, the original two-terminal floating-gate memory architecture (with a MoS_2 channel, h-BN tunneling layer, and graphene floating gate) reported previously (Nat. Commun. 2016, 7, 12725) similarly relies on localized tunneling at contact edges to achieve ultra-low off-state currents below 10^{-14} A and on/off ratios exceeding 10^9. The field enhancement at metal edges is fundamental to such two-terminal designs and is not an artifact of our simulation.

The injected charge, although small due to the limited tunneling area, is sufficient to produce a measurable change in the floating gate potential. This is evidenced by the observed memory window in our experimental devices, which we attribute to localized F-N tunneling consistent with the simulation results.

Moreover, the FGMs with three different tunneling dielectric layer were fabricated, Figure R9 depict the IV courses, showcasing a similar memory behavior. It is demonstrated that the FGM featuring an 18-nm-thick tunneling dielectric layer is capable of charge storage through F-N tunneling, similar to the case of FGMs with a thinner tunneling dielectric layer.

We believe these results comprehensively demonstrate that a sufficient vertical field is indeed established across the Al_2O_3 layer, enabling F-N tunneling in our device.

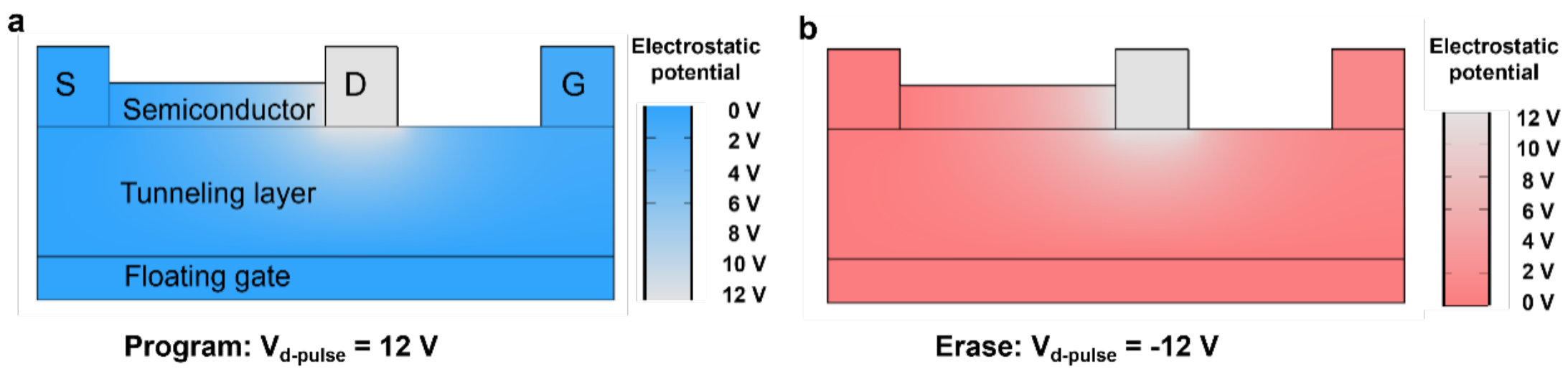

Figure R7: a,b, Simulated electrostatic potential distribution of FGM at the states of program (a) and erase (b).

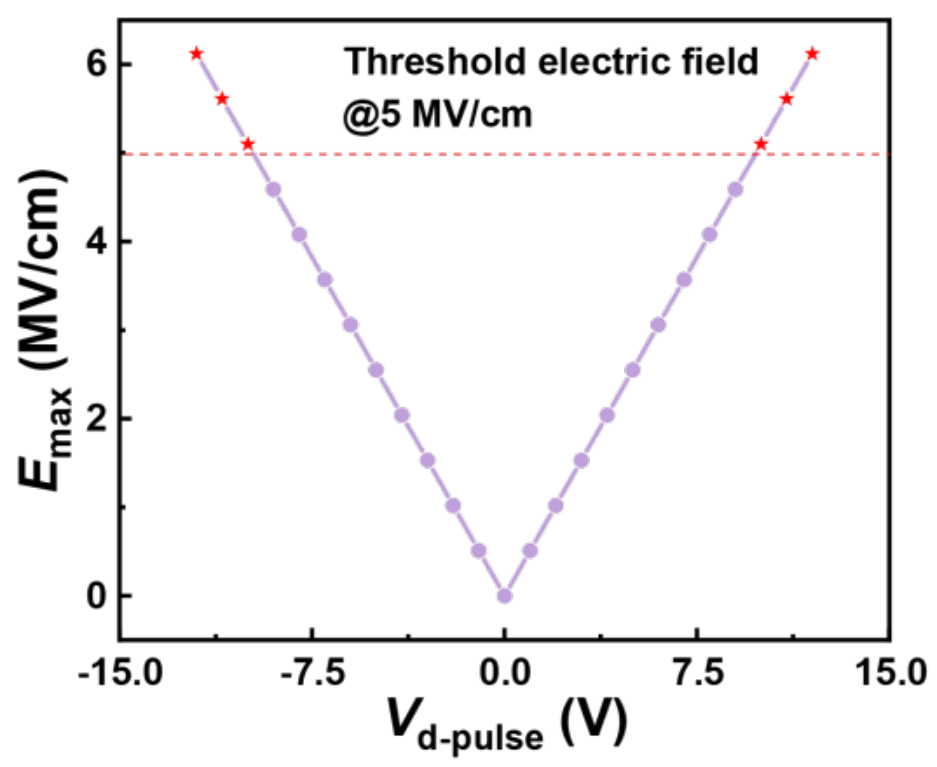

Figure R8: Peak electric field curves in the vertical direction under different drain pulse voltages.

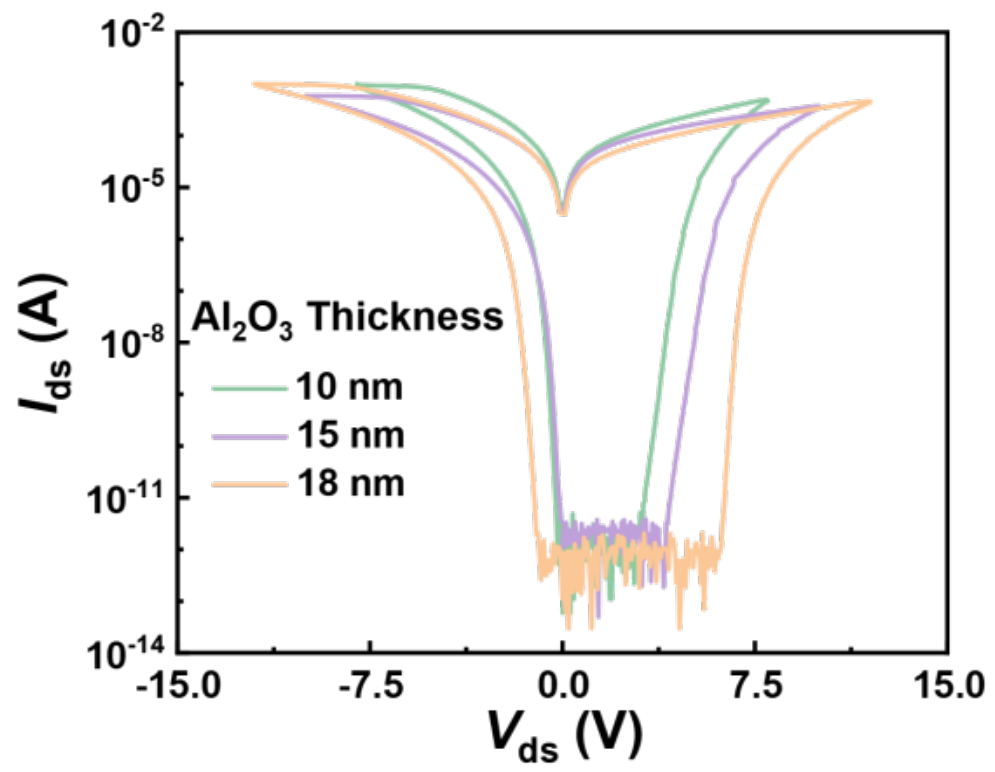

Figure R 9: Sweep transfer characteristics of the FGM with different tunneling dielectric layer.

Table R1: Summary of the simulation outputs under Type-I bias

$V_{d\text{-pulse}}$ (V)	V_{FG} (V)	E_{Avg} (MV/cm)	J_{FN} (A/m²)	E_{max} (MV/cm)	$J_{FN\text{-}Max}$ (A/m²)
-12.0	-4.7507	4.03	3.11e-20	6.12 ★	1.54e-09
-11.0	-4.3548	3.69	4.71e-23	5.61 ★	2.02e-11
-10.0	-3.9589	3.36	1.98e-26	5.10 ★	1.14e-13
-9.0	-3.5630	3.02	1.51e-30	4.59	2.07e-16
-8.0	-3.1672	2.68	1.11e-35	4.08	7.98e-20

-7.0	-2.7713	2.35	2.87e-42	3.57	3.38e-24
-6.0	-2.3754	2.01	4.98e-51	3.06	5.25e-30
-5.0	-1.9795	1.68	2.89e-63	2.55	4.13e-38
-4.0	-1.5836	1.34	1.42e-81	2.04	3.19e-50
-3.0	-1.1877	1.01	5.09e-112	1.53	2.45e-70
-2.0	-0.7918	0.67	9.26e-173	1.02	2.03e-110
-1.0	-0.3959	0.34	0.00e+00	0.51	3.30e-230
+0.0	+0.0000	0.00	0.00e+00	0.00	0.00e+00
+1.0	+0.3959	0.34	0.00e+00	0.51	3.30e-230
+2.0	+0.7918	0.67	9.26e-173	1.02	2.03e-110
+3.0	+1.1877	1.01	5.09e-112	1.53	2.45e-70
+4.0	+1.5836	1.34	1.42e-81	2.04	3.19e-50
+5.0	+1.9795	1.68	2.89e-63	2.55	4.13e-38
+6.0	+2.3754	2.01	4.98e-51	3.06	5.25e-30
+7.0	+2.7713	2.35	2.87e-42	3.57	3.38e-24
+8.0	+3.1672	2.68	1.11e-35	4.08	7.98e-20
+9.0	+3.5630	3.02	1.51e-30	4.59	2.07e-16
+10.0	+3.9589	3.36	1.98e-26	5.10 ★	1.14e-13
+11.0	+4.3548	3.69	4.71e-23	5.61 ★	2.02e-11
+12.0	+4.7507	4.03	3.11e-20	6.12 ★	1.54e-09

2. The retention metric presented in Table S2 requires revision to ensure a fair comparison with the existing literature. The retention values cited for the reference Micro-LED devices correspond to the optical/light-output retention of integrated pixels. In contrast, the 6000 s value reported for this work appears to represent standalone FGM electrical retention. These two metrics are not directly comparable.

Response: Thank you for your professional comment. To avoid any potential misunderstanding for readers, we would divide the comparison of retention into two parts: including the electrical retention characteristics of the standalone FGM and the brightness retention of the integrated device.

a) Update Table S2: Report the optical/luminance retention of the integrated 1M1D Micro-LED pixel for a fair comparison. (For example, the 200 s brightness-retention result from Figure S20, or the newly added 3600 s result from Figure R22)

Response: Thank you for pointing this out. To ensure fairness of the comparison, we divide the retention into two parts: charge retention and brightness retention in the revised Supplementary Table S2.

Table R2 (Supplementary Tabel S2): Summary of the power consumption and stability in Micro-LED devices with various driver technology

Display type	Driver type	Voltage	Leakage current	On/off current ratio	Switch energy	Retention Charge/Brightness	Endurance	Ref.
Micro-LED	In-memory transistors	-35	$>10^{-12}$	$<10^{7}$	17.5 pJ	NA / 60	1000	16
Micro-LED	Memristor	4.3	10^{-8}	$<10^{6}$	~ 10^{6} pJ	$>10^{4}$ / NA	> 480	17
Micro-LED	Memristor	0.17	10^{-9}	$<10^{7}$	2×10^{5} pJ	$>10^{4}$ / NA	10000	18
Micro-LED	Fe-MFFT	8	10^{-14}	4×10^{8}	Sub-pJ	NA / 50	10^{8}	19
Micro-LED	Fe-TFT	6	$>10^{-13}$	$<10^{8}$	Sub-pJ	NA / 100	10^{8}	20
Micro-OLED	Fe-OLET	60	$>10^{-10}$	$<10^{7}$	>300 pJ	10^{4}/ NA	>100	21
Micro-LED	MoS_2- TFT	6.2	6.41×10^{-13}	$<10^{8}$	1192 pJ (for 5 min)	NA	NA	1
Micro-LED	Poly-Si TFT	8	5×10^{-11}	$<10^{8}$	1.2×10^{5} pJ (for 5 min)	NA	NA	5
Micro-LED	ITO-based TFT	8	5.33×10^{-12}	$\sim10^{7}$	11560 pJ (for 5 min)	NA	NA	22
Micro-LED	ITO-FGM	15	7.61×10^{-12} A	$>1.8\times10^{8}$	11.43 pJ	6000 / 200	>4000	This
Micro-LED	ITO-FGM	8	3.84×10^{-12} A	$>1.2\times10^{8}$	3.07 pJ	6000 / 200	>8000	work

b) Clarify Datasets: Explain the relationship between the 200 s brightness-retention data (Figure S20) and the 3600 s data (Figure R22). Although measured over vastly different time durations, the reported brightness losses are remarkably similar, ~9.5% and ~9.4%, respectively. Please clarify if these datasets were measured from different devices, and clearly define the representative luminance-retention value to be used consistently throughout the manuscript.

Response: Thank you for your professional comment. We have simultaneously characterized the brightness retention properties of Micro-LEDs under both practical operating conditions and extreme conditions.

To avoid any misunderstanding for readers, we clarify the conditions of luminance retention more explicitly. In the Figure S20, when a pulse voltage of 15 V applied onto the gate electrode, it would achieve a ultra-high brightness of 4.26×10^6 Cd/m^2 for Micro-LED, at the reading voltage of 2.8 V. And after 200 s of retention, the brightness decay is about 9.5% owing to carrier loss under large on-state current density (~120 A/cm^2), demonstrating the retention capability under ultra-high luminance. Meanwhile, since Micro-LED near-eye display devices typically operate in practical scenarios (100,000 Cd/m^2), a reading voltage of 2.4 V was conducted upon program operation ($V_{g\text{-pulse}}$ = 15 V @ 1 s), with the Micro-LED brightness of 126020 Cd/m^2, showcasing a brightness loss rate was approximately 9.4% over 3600 s time durations, and only 2.38% over 200 s.

In the revised manuscript and Supplementary Information, we have clearly specified both the Micro-LED brightness values and the test conditions when describe the brightness retention characteristics.

[Minor Comments]

1. Correct the y-axis label in Supplementary Figures S15e and S15f from "conts" to "counts."

Response: Thank you for pointing out the typo in Supplementary Figures S15e and S15f. In the revised Supplementary Information, we have corrected the word of “counts”.

2. Supplementary Figure S16 is currently difficult to interpret. While the purpose appears to be demonstrating improved current uniformity across the wafer, the current color scale and order obscure this improvement. Please revise the plot using a clearer, more intuitive, and consistent color scale to effectively support the claim of uniform device performance.

Response: Thank you for your invaluable suggestion. We have adjusted the color scale in the figure to a double-color scheme (Figure R10), thereby enhancing contrast and enabling a more intuitive representation, which more clearly demonstrate that the uniformity of the on-state

current can be improved through control the programming voltage.

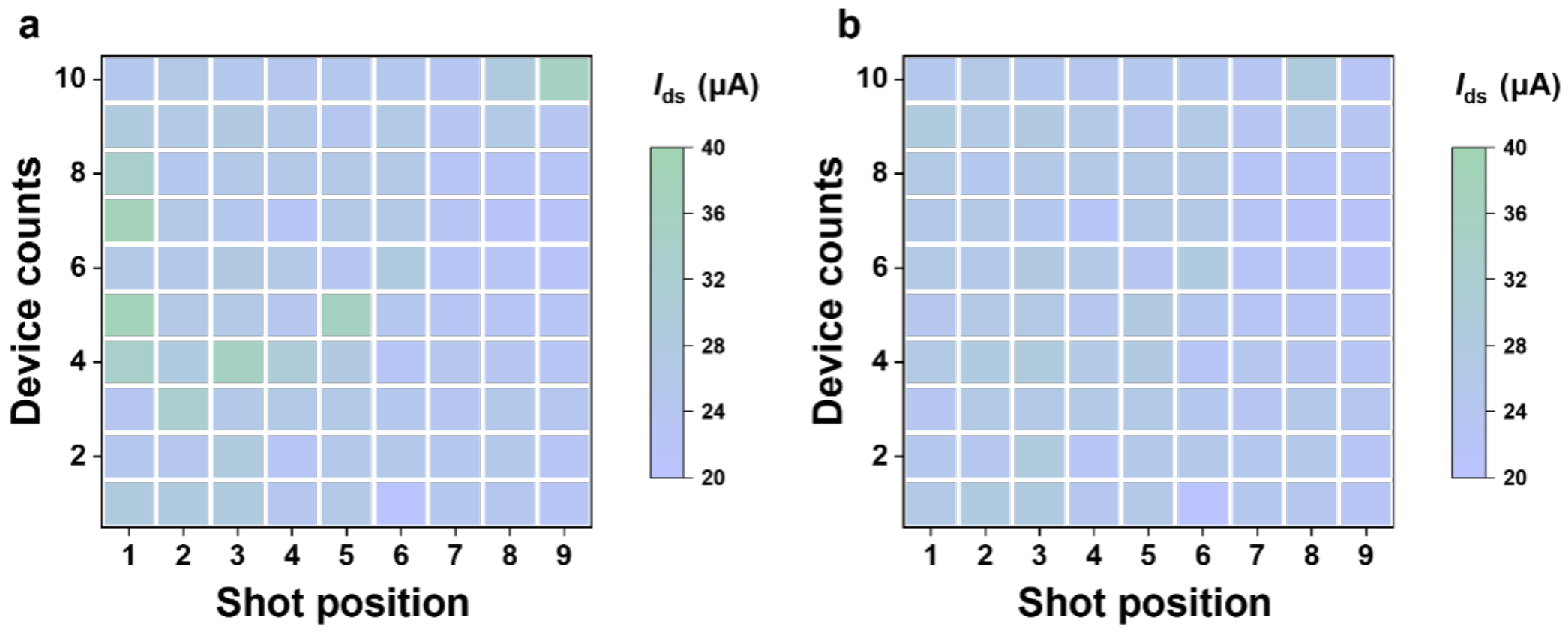

Figure R10 (Revised Supplementary Figure S16): a, Current distribution mapping of different devices upon program operation (+15 V, 0.1 s). **b,** Spatial current distribution of high-current devices after a decrease in the programming voltage (+13 V, 0.1 s).
